# Comparative genomics and genomic diversity of *Pseudomonas syringae* clade 2b-a in Australia

**DOI:** 10.1186/s12866-022-02678-9

**Published:** 2022-11-21

**Authors:** Noel Djitro, Rebecca Roach, Rachel Mann, Paul R. Campbell, Brendan Rodoni, Cherie Gambley

**Affiliations:** 1 School of Applied Systems Biology, La Trobe University, Bundoora, VIC Australia; 2 Department of Agriculture and Fisheries, Ecosciences Precinct, Dutton Park, Australia; 3 Agriculture Victoria Research, Department of Jobs, Precincts and Regions, AgriBio, Bundoora, Australia; 4 Department of Agriculture and Fisheries, Maroochy Research Facility, Nambour, Australia

**Keywords:** *Pseudomonas syringae*, *Cucurbitaceae*, Comparative genomics

## Abstract

**Background:**

A zucchini disease outbreak with unusual symptoms associated with *Pseudomonas* *syringae* clade 2b was identified in Bundaberg, Australia during autumn 2016. To investigate the genetic diversity of the 11 Australian isolates obtained from the outbreak, the genomes were compared to the publicly available *P.* *syringae* strains in phylogroup 2.

**Results:**

Average nucleotide identity refined the *P.* *syringae* clade 2b-a into four clusters (Cluster A, B, C1 and C2), an expansion from the previously identified A, B and C. Australian isolates were in Cluster A, C1 and C2. Genomic analyses highlighted several key factors that may contribute to the virulence of these isolates. Six orthologous groups, including three virulence factors, were associated with *P.* *syringae* phylogroup 2 cucurbit-infecting strains. A region of genome plasticity analysis identified a type VI secretion system pathway in clade 2b-a strains which could also contribute to virulence. Pathogenicity assays on isolates KL004-k1, KFR003-1 and 77-4C, as representative isolates of Cluster A, C1 and C2, respectively, determined that all three isolates can infect pumpkin, squash, watermelon and zucchini var. Eva with different levels of disease severity. Subsequently, type III effectors were investigated and four type III effectors (*avrRpt2*, *hopZ5*, *hopC1* and *hopH1*) were associated with host range. The *hopZ* effector family was also predicted to be associated with disease severity.

**Conclusions:**

This study refined the taxonomy of the *P.* *syringae* clade 2b-a, supported the association between effector profile and pathogenicity in cucurbits established in a previous study and provides new insight into important genomic features of these strains. This study also provided a detailed and comprehensive resource for future genomic and functional studies of these strains.

**Supplementary Information:**

The online version contains supplementary material available at 10.1186/s12866-022-02678-9.

## Background

Queensland is one of the main cucurbit production states in Australia. Approximately 173,864 tonnes of *Cucurbitaceae* product was produced in Queensland during the 2019–20 season [1]. In 2016, unusual symptoms were observed in zucchini fields in Bundaberg including twisted petioles, necrotic leaves, crown-rots and internal fruit-rots. Eleven *P.* *syringae* isolates that obtained from the outbreak were in clade 2b [2] and closely related to other *P.* *syringae* in clade 2b-a [3]. The recent detection of this unusual disease and identification of *P.* *syringae* isolates genetically distinct from previously detected isolates affecting cucurbit in Australia [2] suggest a recent introduction of these isolates. This was most likely through imported seed for production. In Australia, cucurbit seeds for commercial production are imported [4] and *P.* *syringae* is known to be transmitted through seeds [5]. Exploring the genetic diversity of the eleven Australian isolates will provide a better understanding of the differences observed in virulence and host range of each isolate and to identify improved control strategies.

*P. syringae* phylogroup 2 consists of five clades (2a, 2b, 2c, 2d and 2e) containing strains isolated from various environmental and agricultural habitats [6]. Comparative genomics of virulence factors in the *P.* *syringae* complex have shown that strains from phylogroup 2 harbor fewer type III effectors (T3Es) but can produce more phytotoxins, compared to strains from the other primary phylogroups [6–8]. Over the last few years, many *P.* *syringae* strains in phylogroup 2 associated with *Cucurbitaceae* have been isolated and their genetic diversity, pathogenicity and host-association have been studied in detail [2, 3, 9–11]. Pan-genome association analysis of *P.* *syringae* phylogroup 2 cucurbit-infecting strains identified seven genes that potentially contribute to niche adaptation to cucurbit hosts including T3Es *hopZ5*, *hopA1* and its chaperone *shcA*, type VI secreted effector *vgrG*, and three uncharacterised proteins [3]. The presence/absence of some effectors such as *avrRpt2*, *hopZ5*, *hopC1* and *hopH1* were predicted to be associated with host range susceptibility and virulence [11].

Clade 2b-a is a branch group within clade 2b [3] and consists of *P.* *syringae* isolated from *Cucurbitaceae* including pumpkin (*Cucurbita moschata*), melon (*Cucumis melo*), squash (*Cucurbita pepo*), watermelon (*Citrullus lanatus*) and zucchini (*Cucurbita pepo*) [2, 3, 9–11]. This clade emerged from genome-wide homologous recombination between clade 2a and 2b [3]. Three clusters (A, B and C) were identified in clade 2b-a with a unique effector profile on each cluster [11].

In this study, eleven *P.* *syringae* isolates obtained from a zucchini disease outbreak in Australia [2] were compared to the publicly available genomes of *P.* *syringae* phylogroup 2. As these Australian isolates were determined to be *P.* *syringae* clade 2b, their genetic diversity and effector profile in relation to previous reports [3, 11] will further inform the genetic basis of their observed phenotypes. Genome-wide association studies (GWAS) were performed to identify potential genes associated with adaptation to cucurbit hosts and unique genes in clade 2b-a. Functionally significant genomic features including genes in phytotoxins (coronatine, mangotoxin, phaseolotoxin, syringolin, syringomycin, syringopeptin and tabtoxin) and siderophores (achromobactin, pyoverdine and yersiniabactin) biosynthesis pathways, ice nucleation active (INA), T3Es and carbohydrate-active enzyme (CAZyme) domains were identified. Regions of genome plasticity (RGPs) analysis was also performed to identify genes that may have been acquired through horizontal gene transfer. Pathogenicity assays were performed with the spray inoculation method to evaluate the association between predicted T3E repertoires and host range of key *P.* *syringae* clade 2b-a isolates. Together, these analyses further clarified the taxonomy of *P.* *syringae* clade 2b-a strains and identified genomic parameters that may be associated with virulence and host range.

## Results

### Biochemical characterisation

Highly homogeneous carbon utilisation and chemical susceptibility profiles were observed between three Australian isolates (77-4C, KFR003-1 and KL004-k1) using Biolog (Additional file 1: Table S1). All three isolates strongly utilised citric acid, D-fructose, α-D-glucose, glycerol, L-lactic acid, sucrose, and other 12 substrates, however, the abilities to use acetic acid, D-galactose, D-sorbitol and another 6 substrates varied. A minor difference in the utilisation of D-fucose, α-keto-glutaric acid, formic acid and guanidine HCl was observed. All three strains were resistant to some antibiotics including lincomycin, rifamycin SV and vancomycin, and were susceptible to aztreonam, nalidixic acid, minocycline and troleandomycin.

### Pan-genome

The Australian isolates characterised in this study have a genome size between 5.85 to 6.07 Mb (median = 5.90 Mb) with a 59.01 to 59.15 (median = 59.08) %G + C content and 5,030 to 5,252 (median = 5,073) coding sequences (Additional file 1: Table S2). No evidence of plasmids was predicted from any of the Australian isolates. Roary clustered 437,849 coding sequences from 84 strains in phylogroup 2 into 17,286 orthologous groups (OGs) with a total of 3,605 and 447 OGs categorised as core (present in ≥ 99% of strains) and soft (present in 95–99% of the strains) OGs, respectively.

### Population structure analysis

**Fig. 1:**
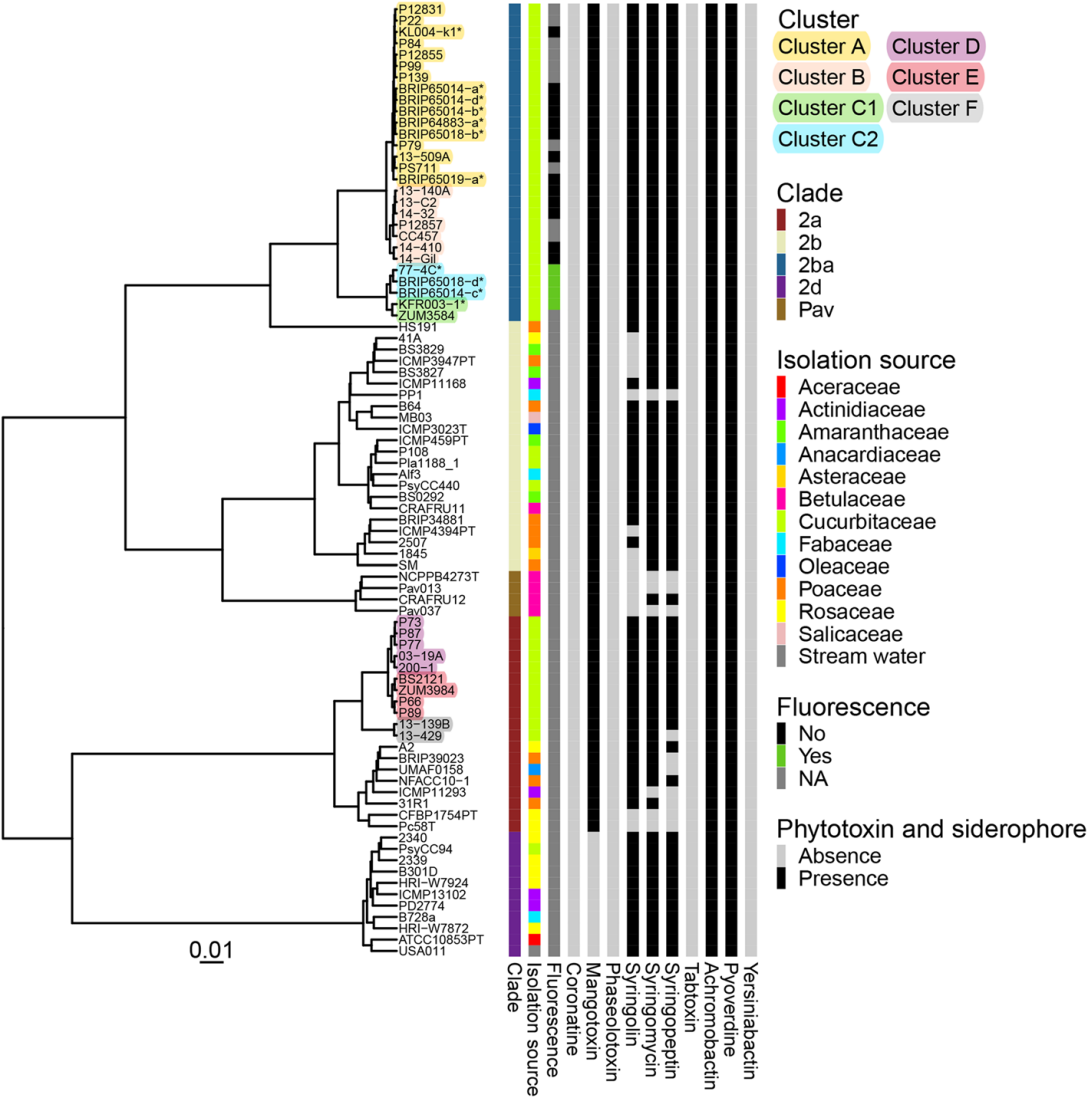
ANIb dendrogram of phylogroup 2 *P.* *syringae* and associated traits. ANIb dendrogram of phylogroup 2 *P.* *syringae* and associated traits. The associated traits include: clades (according to Lacault et al. [11]), clusters, isolation source, fluorescence phenotype, phytotoxin and siderophore profiles. The categories of these traits are noted by colour as shown in the key. NA in fluorescence: data not available. The presence/absence of phytotoxins including coronatine, mangotoxin, phaseolotoxin, syringolin, syringomycin, syringopeptin and tabtoxin and siderophores including achromobactin, pyoverdine and yersiniabactin biosynthesis pathways are depicted and categories are noted by colour as shown in the key. Australian isolates are labelled in the dendrogram with an asterisk (*)

Seven non-fluorescent and four fluorescent Australian *P.* *syringae* isolates [2] were analysed for their genome relatedness. The ANIb dendrogram showed all Australian isolates were in clade 2b-a (Fig. 1). A previous study [11] separated strains of *P.* *syringae* in clade 2b-a into three clusters (A, B and C) using ANIb analysis. The seven non-fluorescent Australian isolates were grouped in Cluster A, which mostly contains zucchini vein clearing disease-causing strains isolated from zucchini from various countries [11]. The four fluorescent isolates were grouped in Cluster C which previously only contained one strain, ZUM3584, which was isolated from squash in Italy in 2005 [3, 11]. In this study, the ANIb dendrogram separated Cluster C into two distinct clusters, namely Cluster C1 and Cluster C2 (Fig. 1). The genomes within these clusters are very closely related (> 99%) as shown by ANIb (Additional file 1: Table S3). Cluster C1 contains two strains (KFR003-1 and ZUM3584) and Cluster C2 contains three isolates from Australia, 77-4C, BRIP65014-c and BRIP65018-d.

### Cucurbit associated orthologous groups (OGs)

Scoary identified six OGs associated with *P.* *syringae* phylogroup 2 cucurbit-infecting strains (Table 1). Five of these OGs were identified in a previous study [3]: two OGs (group_2159 and group_3364) consist of gene sequences with hypothetical proteins, and three OGs with known gene functions including *hopA1*, *shcA* and type VI secretion effector *vgrG*. A new uncharacterised OG, group_2157, was identified in this study. No annotation was obtained for these three hypothetical proteins using InterProScan and Foldseek. Protein sequences of the cucurbit associated OGs in this study are available in Additional file 1: Table S4.

**Table 1:**
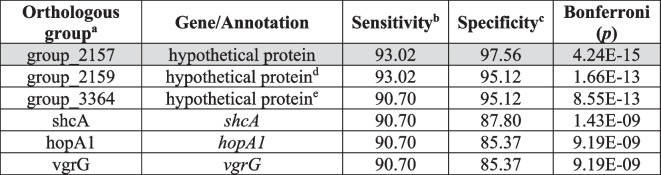
*Pseudomonas syringae* phylogroup 2 cucurbit associated orthologous groups (OGs). The significant OGs were determined by the GWAS conducted with Scoary. OGs in white have been previously identified as cucurbit associated in a study by Newberry et al. [3]. The OG in grey was newly detected in this study. The annotation shows the function or associated protein according to NCBI. Sensitivity and specificity of the OG’s association to cucurbit hosts are shown as percentages

^a^Orthologous group names were generated from Roary

^b^Sensitivity: percentage of cucurbit isolates with the gene present (true positive)

^c^Specificity: percentage of non-cucurbit isolates with the gene absent (true negative)

^d^Locus tag Ga0170668_1061120 in Newberry et al.[3]

^e^Locus tag Ga0170668_106332 in Newbery et al.[3]

### Clade 2b-a unique orthologous groups

A total of 22 OGs were identified as unique to clade 2b-a (Table 2). More than half of the unique clade 2b-a OGs are poorly characterised and thus described as hypothetical proteins. Some OGs had known function and included ATPase, restriction endonuclease subunit S, and taurine catabolism dioxygenase activity. Protein sequences for the clade 2b-a OGs are available in Additional file 1: Table S5.

**Table 2:** Orthologous groups (OGs) unique to *Pseudomonas syringae* clade 2b-a. The significant OGs were determined by the GWAS conducted with Scoary. The annotation shows the function, domain or associated protein according to NCBI, InterProScan or Foldseek. The Cluster of orthologous groups of proteins (COG) category shows the protein function of the OGs as predicted by EggNOG-mapper. The region of genome plasticity (RGP) results from Fig. 6 are included to show where these OGs are positioned in the *P.* *syringae* 77-4C

Orthologous group^a^	Annotation/domain	RGP location^b^	COG category^c^
ycf3	SMEK domain-containing protein	RGP_15	D
group_7141	SEC-C domain containing protein	RGP_15	S
group_7142	hypothetical protein	RGP_15	-
rep_2	UvrD-helicase domain containing protein	RGP_15	-
group_7144	hypothetical protein	RGP_15	S
group_7145	TauD/TfdA family dioxygenase	RGP_15	-
group_7146	hypothetical protein	RGP_15	-
ravA	ATPase	RGP_32	-
group_7115	bpX5 domain^d^	RGP_32	V
group_7116	6ND4^e^	RGP_32	-
group_7117	bpX6 domain containing protein	RGP_32	S
group_7138	Restriction endonuclease subunit S	RGP_13	-
group_7206	YrhB domain-containing protein	RGP_6	L
group_7088	ATPase	RGP_18	-
group_7089	DUFF2280 domain containing protein	RGP_18	Q
group_7091	hypothetical protein	RGP_18	-
group_7092	hypothetical protein	RGP_18	-
group_7179	Excisionase	RGP_20	S
group_7180	hypothetical protein	RGP_20	-
group_3605	ATPase	RGP_20	L
group_7174	hypothetical protein	RGP_3	-
group_7200	hypothetical protein	Not available	S

^a^Orthologous group names were generated from Roary

^b^RGP location based on the reference genome of 77-4C in Fig. 6

^c^COG category: (D: cell cycle control, cell division, chromosome partitioning; L: replication, recombination and repair; Q: secondary metabolites biosynthesis, transport and catabolism; S: function unknown; V: defence mechanism; -: not available in database)

^d^domain predicted using InterProScan

^e^protein structure was predicted using AlphaFold and homologous structures were identified using Foldseek with the RCSB PDB database

### Carbohydrate active enzymes

**Fig. 2:**
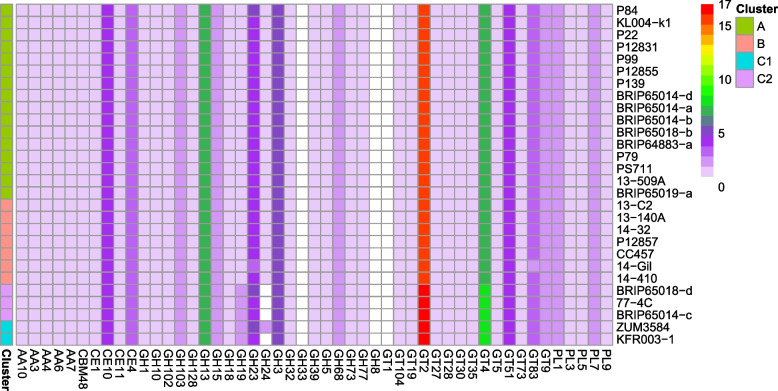
Heatmap of the carbohydrate active enzyme profile of *Pseudomonas syringae* clade 2b-a. This analysis was generated using the CAZy database. 2b-a cluster is indicated on the left of the heatmap according to the colours in the key. *P.* *syringae* strains are listed on the right and CAZyme domains are listed on the bottom. The colour of each CAZyme family indicates the number of protein domains present in the strains genome as shown in the key. The CAZy database labels refer to: glycoside hydrolase (GH), glycosyltransferases (GT), polysaccharide lyases (PL), carbohydrate esterases (CE) and auxiliary activities (AA) and carbohydrate binding module (CBM)

Each of the 11 Australian isolates and 17 publicly available strains in clade 2b-a possessed 94 to 101 functional CAZyme domains (Fig. 2). The profile of CAZyme was consistent across all isolates in the clade, with some differences identified in the number of domains present for the glycoside hydrolase (GH) and glycosyltransferase (GT) families. Cluster C1 and C2 strains have one additional domain from the GH19, GT2 and GT4 families, while isolate ZUM3584 had an additional CAZyme domain belonging to the GH24 family. The corresponding protein sequences from families with an additional domain are available in Additional file 1: Table S6.

### Siderophore, ice nucleation activity and phytotoxin profiles

*P. syringae* strains in clade 2b-a showed similar profiles for phytotoxin and siderophore biosynthesis pathways (Fig. 1). Strains in clade 2b-a were previously identified as INA negative [2, 9] and a deletion of approximately 1.8 kb in the middle of the INA gene coding sequence was observed in all strains of this clade (data not shown). Phytotoxin biosynthesis pathways for mangotoxin, syringolin, syringomycin and syringopeptin were present in the genome of all clade 2b-a strains. Siderophore biosynthesis pathways for achromobactin and pyoverdine were present in the genome of all clade 2b-a strains, but not yersiniabactin (Fig. 1).

### Fluorescence phenotype

**Fig. 3:**
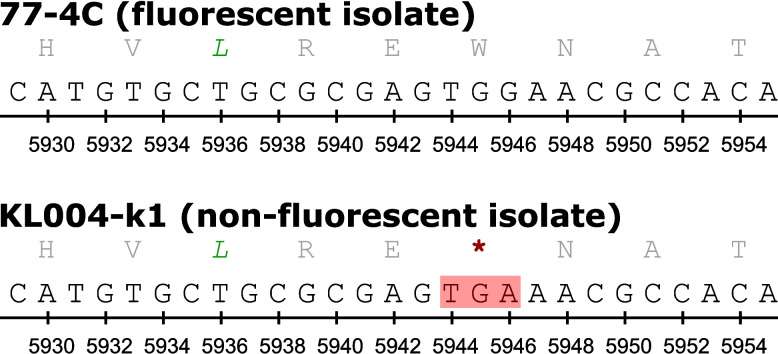
Comparison of gene sequences of non-ribosomal peptide synthetase IV between fluorescent and non-fluorescent *P.* *syringae* clade 2b-a. Single letter amino acid and corresponding DNA codon are shown. Isolates 77-4C and KL004-k1 represent the fluorescent and non-fluorescent isolates, respectively. The red highlight indicates a premature stop codon hypothesised to cause the non-fluorescence phenotype

As a difference in fluorescence phenotype was observed within *P.* *syringae* clade 2b-a strains [2, 9] and a pyoverdine mutation was previously shown to generate a non-fluorescence phenotype in *P.* *syringae* [12], genes in the pyroverdine biosynthesis pathway of each isolate with known fluorescence phenotype were compared. The alignment revealed a premature stop codon on one of the non-ribosomal peptide synthetase (NRPS) genes (Fig. 3) that corresponded to pyoverdine sidechain peptide synthetase IV in strain 1448A (locus tag PSPPH_1926). The premature stop codon was confirmed with reverse transcription-polymerase chain reaction on isolates 77-4C, KFR003-1 and KL004-k1 (data not shown).

### Type III effectors

**Fig. 4:**
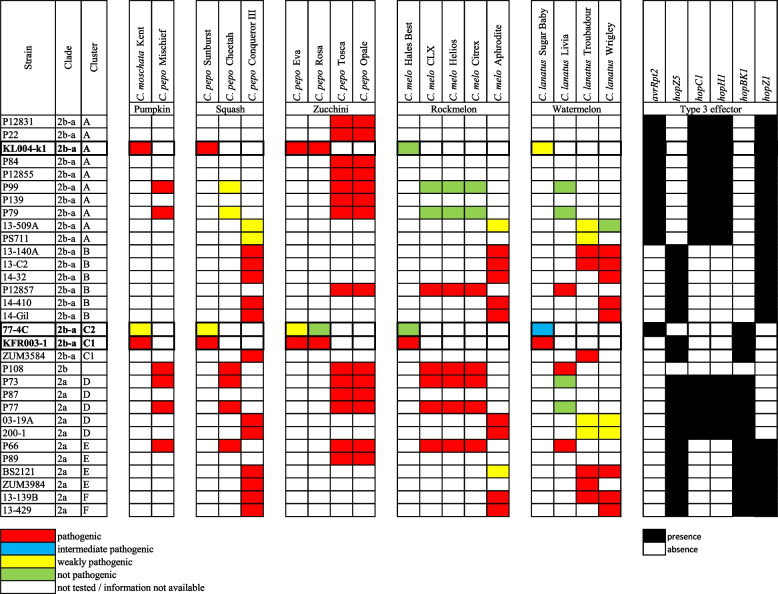
Association between host and type III effector profile of *Pseudomonas syringae* phylogroup 2 cucurbit-infecting strains. Results from this study are indicated with bold text and borders. All other results are compiled from previous studies [3, 9–11]. Strain names, clade and cluster are listed on the left of the table. Common and scientific names of hosts are listed across the top. Pathogenicity results are shown as red, blue, yellow and green as described in the key. Associated type III effectors are listed at the top left. The colour in T3E family columns indicates effector presence/absence as shown in the key where black = present and white = absent

The T3E repertoires of the seven non-fluorescent Australian isolates were identical to strains in Cluster A (Fig. 4). Strains in Cluster A were known to possess *avrRpt2* (*avrRpt2*^+^) but not *hopZ5* (*hopZ5*^−^), and the opposite was observed in Cluster B which possess *hopZ5* (*hopZ5*^+^) but not *avrRpt2* (*avrRpt2*^−^) [11]. Both Cluster A and B also possess *hopC1* (*hopC1*^+^) and *hopH1* (*hopH1*^+^). A similar presence/absence pattern of *hopZ5* and *avrRpt2* was also observed between Cluster C1 and C2. Cluster C1 was *avrRpt2*^−^-*hopZ5*^+^ while Cluster C2 was *avrRpt2*^+^-*hopZ5*^−^. Both Cluster C1 and C2 did not possess *hopC1* (*hopC1*^−^) and *hopH1* (*hopH1*^−^) which was present in Cluster A and B but do possess *hopBK1* which is absent in Cluster A and B. *hopBK1* was also present in all clade 2a strains isolated from cucurbit (Cluster D, E and F). The complete effector profile of *P.* *syringae* isolated from cucurbit in this study is available in Additional file 1: Figure S1.

### Pathogenicity assays

**Fig. 5:**
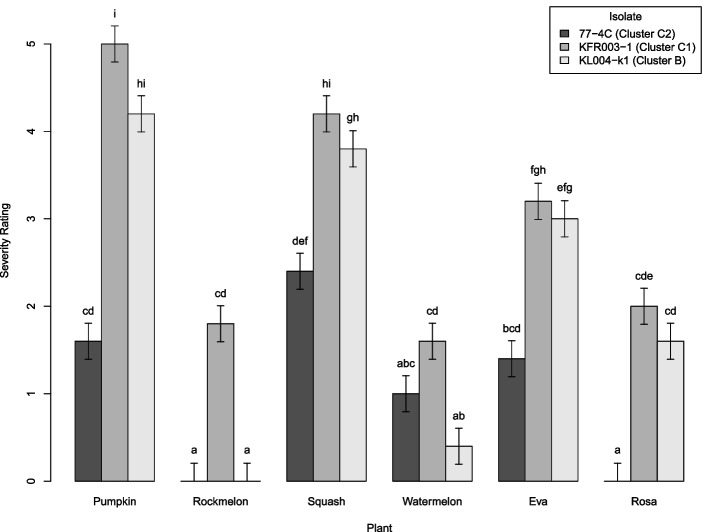
Mean disease severity rating of isolates 77-4C, KFR003-1 and KL004-k1 on cucurbit hosts. Isolates are identified by colour as shown in the key. ‘Eva’ and ‘Rosa’ are varieties of zucchini. The experiment was conducted in a glasshouse. Five replicates were used for each isolate on each plant variety. Disease severity was rated from 0–5 which 0 = no symptom, 1 = leaf spots without necrotic lesions, 2 to 5 represented < 25%, 25 to 49%, 50 to 74% and ≥ 75%, necrotic lesions covering the leaf surface, respectively. The vertical lines represent one standard error mean (0.4123) calculated with GenStat. Significant differences are shown with a letter above the bar (LSD = 1.1624, α = 0.05)

Isolates KL004-k1 (Cluster A), KFR003-1 (Cluster C1) and 77-4C (Cluster C2) were used as representative isolates from each cluster for pathogenicity assays. Spray inoculation was used in the pathogenicity assays to enable better comparisons of the relation between T3Es and host range with other studies that used the same inoculation method [3, 11]. In this study the symptoms on the leaf ranged from leaf spots to necrosis of the whole leaf (Additional file 1: Figure S2 and Figure S3). The severity rating was strongly affected by isolates (F_2,72_ = 32.12, *P* < 0.001) and plant hosts (F_5,72_ = 30.24, *P* < 0.001) (Fig. 5). There was a significant interaction effect between bacterial isolates and plant hosts (F_10,72_ = 2.5, *P* = 0.012), however, the independent variable effects of isolates and plants were around 12 times higher than the interaction effect. All three isolates were pathogenic to pumpkin, squash, watermelon and zucchini var. Eva. A previous report showed that isolate 77-4C was not able to infect zucchini variety Rosa with a crown inoculation method [2]. Within Cluster C, isolate KFR003-1 (Cluster C1) was more virulent than 77-4C (Cluster C2) on pumpkin, squash and zucchini var. Eva. In watermelon, isolate KFR003-1 was more virulent than isolate KL004-k1 while isolate 77-4C was being intermediate. Isolates KL004-k1 and77-4C did not infect rockmelon in this study.

### Regions of genome plasticity

**Fig. 6:**
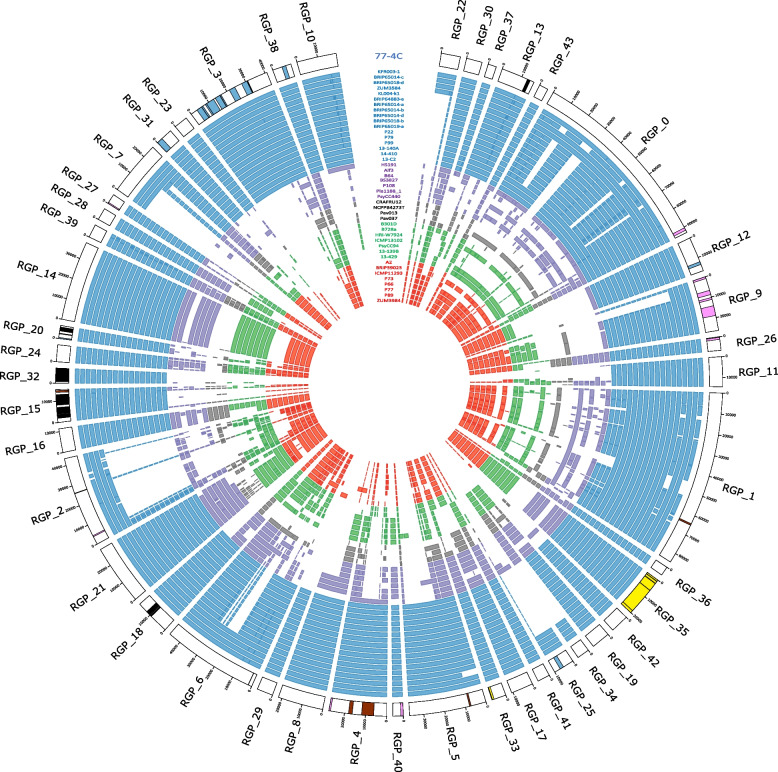
Comparison of regions of genome plasticity (RGPs) within strains of *P.* *syringae* phylogroup 2. RGPs were determined using Ppanggolin. Isolate 77-4C was used as the reference genome (outer ring with black border). Colours in the outer ring represent clade 2b-a unique OGs (black), CAZymes (pink), type III effectors (brown), phytotoxins (yellow) and type VI secretion system pathway (blue). Genomes from selected strains used in this study were mapped to the RGPs of 77-4C, shown here with the 43 coloured inner rings. Clade is denoted with colour of the strain name and rings (blue: 2b-a, purple: 2b, grey: Pav, green: 2d and orange: 2a). White sections indicate that region of the RGP is absent. Image was generated using Circos

The Australian isolates in clade 2b-a harbour 44 to 59 RGPs (Additional file 1: Table S2). Analysis of complete genome of isolate 77-4C showed 13.52% of the total genome was represented by 44 RGPs (Fig. 6; Additional file 1: Table S7). Most of the clade 2b-a unique OGs (Table 2) and some of the CAZyme, T3E and syringolin biosynthesis genes were also identified in these RGPs. For example, genes involved in syringolin biosynthesis pathway were identified mostly in RGP_35. Kyoto Encyclopedia of Genes and Genomes (KEGG) identified type VI secretion system pathways (map03070) within RGPs, mostly located in RGP_3. The complete region of RGP_3 is present in its entirety in all clade 2b-a strains but only partially present in strains from other phylogroup 2 strains such as CRAFRU11 and 31R1 (Additional file 1: Figure S4). Cucurbit associated OGs (Table 1), INA and genes in siderophore biosynthesis pathways were not detected in these RGPs.

## Discussion

In this study, we further investigate the Australian *P.* *syringae* isolates in clade 2b that were previously isolated from a zucchini disease outbreak with unusual field symptoms including twisted petioles, necrotic leaves, crown-rot and internal fruit-rot during autumn 2016 [2]. Population structure analysis using ANIb identified the Australian isolates belonged to clade 2b-a. No plasmid was predicted in the Australian isolates, which is consistent with findings from Newberry et al. [3]. The ANIb distance analysis indicates that the previously described and underrepresented Cluster C may be two clusters: Cluster C1 and C2. Further phylogenetic analysis is needed to confirm this. The theoretical role of type III effectors and other genomic elements in pathogenicity on cucurbits are discussed below to provide insight into the diversity and niche adaptation of *P.* *syringae* strains.

### Type III effectors and pathogenicity

T3Es are one of the primary *P.* *syringae* virulence factors to supress plant immunity. A previous study reported that the host range for *P.* *syringae* phylogroup 2 cucurbit-infecting strains was associated with presence/absence of *avrRpt2*-*hopZ5*, with *avrRpt2*^−^-*hopZ5*^+^ indicating a ‘broad’ host range strain (pathogenic to cucumber, melon, squash and zucchini) whereas an *avrRpt2*^+^-*hopZ5*^−^ T3E profile indicated a ‘narrow’ host range strain (pathogenic to squash and zucchini) with spray inoculation method [11]. Based on these guidelines, isolates 77-4C and KL004-k1 would be predicted to have a ‘narrow’ host range while isolate KFR003-1 would be predicted to have a ‘broad’ host range. However, the previous pathogenicity assays using crown inoculation showed that KL004-k1 was highly virulent to rockmelon [2]. Different inoculation methods can be a contributing factor to the observed differences in the host susceptibility. Therefore, the pathogenicity assays of the three isolates in this study were retested using the same spray inoculation method mentioned in [11] to allow for a more direct comparison of the pathogenicity results and T3Es profiles with this other study.

The correlation of the susceptibility of rockmelon and watermelon with the presence/absence of *avrRpt2*-*hopZ5* and *hopC1*-*hopH1* in three isolates was confirmed. In this study, rockmelon was susceptible to isolate KFR003-1 (*avrRpt2*^−^, *hopZ5*^+^) but not to isolates 77-4C and KL004-k1 (*avrRpt2*^+^, *hopZ5*^−^). Watermelon was susceptible to all three isolates with differing severity. Isolate KFR003-1 (*hopC1*^−^, *hopH1*^−^) was highly pathogenic to watermelon, while isolate KL004-k1 (*hopC1*^+^, *hopH1*^+^) showed low pathogenicity. Isolate 77-4C (*hopC1*^−^, *hopH1*^−^) had similar *hopC1*-*hopH1* profile with KFR003-1, however, was moderately pathogenic on watermelon. Differences of copy number (*hopAH1*, *hopW1*) or presence/absence of effectors between isolate 77-4C (contains *avrRpt2*, *hopAH1* and *hopW1*, but not *hopAW1* and *hopZ5*) and KFR003-1 (contains *hopAH1*, *hopAW1*, *hopW1* and *hopZ5*, but not *avrRpt2*) could explain the difference in the observed pathogenicity. Interestingly, there is no difference in the disease severity between isolate KFR003-1 (*avrRpt2*^−^, *hopZ5*^+^) and KL004-k1 (*avrRpt2*^+^*, hopZ5*^−^) on pumpkin, squash and two varieties of zucchinis, suggesting the presence/absence of *avrRpt2*-*hopZ5* only affects host range.

The disease severity of isolate 77-4C was lower than KFR003-1 and KL004-k1 in pumpkin, squash and zucchini, indicating other factors could be influencing the disease severity. The main difference in effectors for these three isolates is the presence/absence of the *hopZ* effector family, which is present in isolates KL004-k1 (*hopZ1*) and KFR003-1 (*hopZ5*) but not in isolate 77-4C (Fig. 4). Sequence alignment of these *hopZ* effector family using blastp identified 25% similarity, however, having similar structure and can be superimposed with a RMSD of 1.233 for 2152 out of 3087 atoms using PyMOL [13] (Additional file 1: Figure S5). These findings suggest that the absence of *hopZ* family in *P.* *syringae* can reduce pathogenicity. Deletion of *hopZ5* reduced the pathogenicity of *P.* *syringae* pv. *actinidiae* M228 in *Actinidia chinensis* (kiwifruit) var. *chinensis* ‘Hongyang’ [14] and *hopZ1* was known to promote infection of *P. syringae* pv. *glycinea* BR1 in soybean [15]. *P.* *syringae* phylogroup 2 strain P108 does not encode genes from the *hopZ* family, however, it is still pathogenic to pumpkin, squash, watermelon and zucchini [11]. Because of this difference of effector profile in strain P108 compared to other strains of *P.* *syringae* in phylogroup 2, this implies that different effector combinations could result in pathogenicity in cucurbit (Additional file 1: Figure S1). Gene knock-out analyses of *hopZ1* in KL004-k1 and *hopZ5* in KFR003-1 are required to further investigate the role of this effector family in pathogenicity and host range of *P.* *syringae* clade 2b-a isolates.

### Genome-wide association studies

GWAS was performed to identify genes associated with *P.* *syringae* phylogroup 2 cucurbit-infecting strains and conserved OGs in clade 2b-a. A total of six OGs were associated with *P.* *syringae* phylogroup 2 cucurbit-infecting strains. Three of these six OGs were virulence factors including *hopA1*, *shcA* and *vgrG*, suggesting these OGs may be involved in niche adaptation. Functional analysis of *hopA1* and *shcA* indicated the effectors encoded by these genes have a significant role in virulence, host specificity, motility and biofilm formation in *P.* *cichorii* [16]. In *Agrobacterium tumefaciens* the *vgrG* gene encodes a spike protein for type VI effector translocation [17]. The other three OGs identified in this study were uncharacterised, including the newly identified group_2157 OG. Two OGs, *hopZ5* and a hypothetical protein (Ga0170668_1056171), were previously identified to be associated with cucurbit adaptation in previous study by Newberry et al. [3] but not in this study as a more diverse set of *P. syringae* genomes were used. In the current study, GWAS also identified a total of 22 unique clade 2b-a OGs (Table 2). Most of the genes identified in the analyses are poorly characterised and require functional studies to understand their importance in the adaptation to cucurbit host and the emergence of clade 2b-a strains.

### Regions of genome plasticity

Some of the virulence factors and associated genes that were identified by GWAS in this study, such as clade 2b-a unique OGs, CAZymes, T3Es, and the syringolin biosynthesis genes were identified in RGPs. Additionally, the complete type VI secretion system pathway that is present in all strains in clade 2b-a was identified within RGPs (Fig. 6, Additional file 1: Figure S4), suggesting that these genes were acquired through horizontal gene transfer. The type VI secretion system is associated with pathogenicity and deletions in this system has significantly reduced the pathogenicity in *P.* *syringae* pv. *actinidiae* M228 in kiwifruit [18]. The role of the type VI secretion system in *P.* *syringae* is not yet well understood, however, these results suggests that it is a promising avenue of future work. The identified RGPs could also contribute to the adaptation and virulence of *P.* *syringae* clade 2b-a strains. In depth analysis of RGP including mobile genetic elements, genomic islands and bacteriophages inside RGPs is required to understand the source and evolution of the *P.* *syringae* genome.

### Siderophores

An identical siderophore profile was identified in clade 2b-a strains based on our identification criteria. However, a difference in fluorescence phenotype within Australian isolates in Cluster A and Clusters C1-C2 was noted in a previous study [2]. Pyoverdine is the siderophore that confers a fluorescence phenotype in *P.* *syringae* [12] and an immature pyoverdine product in a *P.* *syringae* mutant will result in a non-fluorescence phenotype [19]. A premature stop codon in one of the NRPS genes in the pyoverdine biosynthesis pathway for Australian isolates in Cluster A (represented by KL004-k1 in Fig. 3) was hypothesised to be the cause of the non-fluorescence phenotype. This hypothesis is supported by a deletion of the NRPS gene in the *P.* *syringae* pv. *actinidiae* pyoverdine biosynthesis pathway using single-plasmid CRISPR-Cas9 resulting in a non-fluorescence phenotype [20]. This difference in phenotype complicates the identification process of *P.* *syringae* since *P.* *syringae* was originally described as a fluorescent phytopathogen [21] and this differential phenotype is still used in diagnostics today.

### Phytotoxins

*P. syringae* phylogroup 2 was known to contain more phytotoxin biosynthesis genes than other phylogroups [7]. An identical phytotoxin profile was observed between strains within clade 2b-a, including the presence of mangotoxin, syringolin, syringomycin and syringopeptin biosynthesis pathways. This suggests the presence of phytotoxins does not affect the host susceptibility to *P.* *syringae*. Mangotoxin is an antimetabolite that inhibits ornithine acetyl transferase and could cause apical necrosis [22], syringomycin and syringopeptin can form pores in the plant plasma membrane [23] and syringolin is a proteasome inhibitor [24]. Coronatine, phaseolotoxin and tabtoxin were not reported as typical phylogroup 2 phytotoxins [25, 26] and this is supported by the phytotoxin profile identified in this study.

### Carbohydrate active enzymes

The CAZyme profile showed differences in GT2, GT4, GH19 and GH24 CAZymes within clade 2b-a. No KEGG ortholog or pathway was found for the gene that corresponded to the domain difference in the GT2 family. Genes that correspond to the domain differences in GH19 and GH24 were associated with chitinase and lysozyme activities, respectively [27], which are reported not to contribute to plant cell-wall degradation [28]. KEGG identified that genes corresponding to the difference in GT4 family were associated with biofilm formation and an ortholog to *pslI* (map02025). A previous study showed an altered psl-like polysaccharide in *P. syringae* UMAF0518 could affect the adhesion and motility of bacteria on the mango leaf surface [29]. This suggests the different number of biofilm genes between clusters could affect the adaptation of *P.* *syringae* to cucurbit hosts.

## Conclusions

This study clarifies the taxonomy and provides insight into genes that could contribute to the virulence and host range of *P.* *syringae* clade 2b-a. The *P.* *syringae* clade 2b-a was separated into 4 clusters (Cluster A, B, C1 and C2) based on ANIb. Differences in effector profile were observed within the four clusters of clade 2b-a. The presence/absence patterns of *avrRpt2* and *hopZ5* that were observed between Cluster A and B were also observed between Cluster C1 and C2. *hopC1* and *hopH1* were present in Cluster A and B but not in Cluster C1 and C2. The pathogenicity of isolates 77-4C, KFR003-1 and KL004-k1 to several cucurbit hosts were associated with the presence/absence of T3Es including *avrRpt2*, *hopZ5*, *hopC1* and *hopH1*. This study predicted the absence of the *hopZ* effector family in *P.* *syringae* could contribute to disease severity. GWAS in this study revealed a new uncharacterised OG, group_2157, that could contribute to cucurbit adaptation and 22 OGs unique to clade 2b-a. The CAZyme profile identified one additional domain of GH24 family in Cluster C1 and C2 which was associated with biofilm formation. Analysis of genes in the pyoverdine biosynthesis pathway revealed the non-fluorescent Australian isolates had a premature stop codon in one of the NRPS genes that we predict has resulted in a non-fluorescence phenotype. Analysis of *P.* *syringae* phylogroup 2 RGPs identified the presence of the type VI secretion system in clade 2b-a strains that could contribute to virulence. Further exploration into RGPs and functional studies are required to provide more understanding of the virulence, adaptation and evolution of *P.* *syringae* to cucurbit hosts.

## Methods

### Australian isolates

Eleven Australian *P.* *syringae* isolates which were previously isolated from a zucchini outbreak in Bundaberg, Queensland, Australia, [2] were used in this study. DNA extraction, sequencing and trimming were described previously [2]. To make a complete reference strain genome for clade 2b-a, long-reads of isolate 77-4C was generated using MinION (Oxford Nanopore Technologies) according to the manufacturer’s instructions. Trimmed reads were assembled using Unicycler version 0.4.8 [30] implementing SPAdes version 3.13.0 [31]. Contigs that were less than 200 bp in length were removed using Seqkit version 0.13.2 [32] for GenBank submission. Genome assembly statistics were obtained using QUAST version 5.0.2 [33] with default parameters. The presence of plasmids in the Australian isolates was analysed using SPAdes version 3.13.0 [31].

### Biochemical characterisation

Bacteria were cultured from a -80℃ glycerol stock onto KB agar and incubated at 27℃ for 24 h. The various carbon source utilisation and chemical sensitive assays were determined using a Biolog GenIII MicroPlate (Biolog, Inc) according to the manufacturer’s protocol as follows. A single colony of *P.* *syringae* was streaked onto BUG agar and incubated at 27℃ for 24 h. A single colony from BUG agar was taken using a sterile inoculator swab and inoculated into IF-A inoculating fluid. Each well of the Biolog plate was filled with 100 μl inoculating fluid and incubated at 27℃ for 48 h. Reactions were considered positive and negative when the OD_600_ value above 50% and below 25%, respectively. Reactions were considered borderline when the OD_600_ was between these two values.

### Population structure analysis

A total of 73 *P.* *syringae* genomes in phylogroup 2 publicly available from the National Center for Biotechnology Information (NCBI) were used to compare the diversity of Australian isolates. Clade 2c was not included as it consists of non-pathogenic strains [6]. Detailed information of all strains used in this study is provided in Additional file 1: Table S8. The degree of genome relatedness of isolates in this study was measured by calculating average nucleotide identity (ANI) using pyani version 0.2.11 [34] with ANIb as previously described [11]. A Newick tree was created from ANIb pairwise distance matrix using using RStudio (https://www.rstudio.com/) with ape package version 5.5 [35].

### Pan-genome and GWAS analysis

Each genome in this study was annotated using Prokka version 1.14.6 [36] with default parameters for annotation consistency. The pan-genome was characterised using Roary version 3.13.0 [37] with the -s option to prevent paralog splitting and a minimum percentage identity of 90%. Orthologous groups (OGs) of genes associated with *P.* *syringae* phylogroup 2 cucurbit-infecting strains and unique to clade 2b-a were identified using Scoary version 1.6.16 [38]. Cucurbit associated OGs were identified with ≥ 85% for sensitivity and specificity and Bonferroni *p* ≤ 10^–5^ thresholds. A Newick tree from ANIb was used as an input tree for Scoary. Cluster of orthologous groups of proteins (COG) category was assigned using EggNOG-mapper version 2.1.3 [39] with default parameters, based on the EggNOG 5.0 database [40]. Protein sequence of genes identified in this analysis were compared to the NCBI database for annotation. When the hypothetical protein annotation was obtained using NCBI, possible domains of the hypothetical protein were annotated using InterProScan version 5.57–90.0 [41]. When no annotation was obtained from NCBI and InterProScan, protein structure was predicted using AlphaFold version 2.2.3 [42] and homologous structures were identified using Foldseek version 3.915ef7d [43] with the RCSB PDB database [44].

### Regions of genome plasticity

Some genes that contribute to virulence, adaptation or evolution in bacteria may be located in regions of genome plasticity (RGPs) [45]. RGPs of *P.* *syringae* strains in this study were predicted using PpanGGOLiN version 1.1.136 [46] with the panRGP workflow [47] to identify genomic regions that were possibly acquired through horizontal gene transfer. Isolate 77-4C is the only complete genome available in clade 2b-a and was generated in this study for use as the reference genome. Genes identified in RGPs of isolate 77-4C was reinterpreted using Kyoto Encyclopedia of Genes and Genomes (KEGG) [48–50] to find possible molecular interactions. Genomes of representative strains were mapped to the RGPs of 77-4C using blastn [51]. The presence of clade 2b-a unique OGs, cucurbit associated OGs, INA, T3E, phytotoxins and siderophores genes inside the RGPs were annotated. The RGPs of isolate 77-4C and genome alignment of representative isolates were visualised using Circos version 0.96–8 [52].

### Identification of functionally significant genomic features

Genes for INA, type III effectors (T3E) and genes in the biosynthesis pathways of phytotoxins (coronatine, mangotoxin, phaseolotoxin, syringolin, syringomycin, syringopeptin and tabtoxin) and siderophores (achromobactin, pyoverdine and yersiniabactin) were investigated. The *P.* *syringae* T3Es protein sequences were previously described [8] and the protein sequences for INA, phytotoxins and siderophores are available in Additional file 1: Table S9–11. The genes were investigated by querying protein sequences from each isolate against the database using blastp [51] with parameters set at an e-value < 10^–5^, percentage identity > 80% and 90% of alignment length covering the longer sequence to avoid short sequence mismatch. Phytotoxins were identified and validated using the method described here [7]. Siderophores were considered present if half of the biosynthesis genes were found in the genome. Proteins that corresponded to carbohydrate active enzymes (CAZymes) [27] were identified using dbCAN2 version 2.0.11 [53] with the hmmer method.

### Pathogenicity assays

Three isolates including 77-4C, KFR003-1 and KL004-k1 and six plant varieties including pumpkin (*Cucurbita moschata* var. Kent Special Hybrid 864), rockmelon (*Cucumis melo* var. Hales Best), squash (*Cucurbita pepo* var. Sunburst), watermelon (*Citrullus lanatus* var. Sugar Baby) and two zucchini varieties (*Cucurbita pepo* var. Eva and Rosa) were used in these assays. A preliminary small scale experiment using spray inoculation [11] was conducted in a growth cabinet with two varieties each of zucchini, rockmelon and watermelon (data in Additional file 1: Figure S6). The main, larger experiment was then done in a glasshouse using the same method with all six plants. In both growth cabinet and glasshouse experiments, five replicates were used for each isolate on each plant variety. Bacterial suspensions (approximately 1 × 10^8^ CFU/ml) in distilled water were prepared from cultured bacteria on King’s B agar at 27 °C for 24 h. Seedlings were germinated at the second true leaf stage and maintained in glasshouse conditions. The spray inoculation method previously described [11] was used to evaluate the association between T3E and host range with modification as follows: plantlets were moved into a controlled glasshouse with a temperature of 20℃, 80% relative humidity and 12 h photoperiod one day before inoculation. Plantlets were inoculated by spraying the bacterial suspension on the abaxial side of the first true leaf until runoff and maintained for 7 days. Disease severity was scored on day 7 post-inoculation visually [2]. A visual rating from 0 to 5 was used for scoring the disease severity in which 0 = no symptom, 1 = leaf spots without necrotic lesions, 2 to 5 represented < 25%, 25 to 49%, 50 to 74% and ≥ 75%, necrotic lesions covering the leaf surface, respectively (Additional file 1: Figure S2). Data was analysed using GenStat [54] with two way analysis of variance and Fisher’s protected least significant difference (LSD) to indicate significant differences.

## Supplementary Information

**Additional file 1:** **Table S1.** Biolog results of isolate 77-4C, KFR003-1 and KL004-k1. **Table S2.** Assembly statistics of *Pseudomonas syringae* strains used in this study. Isolates from this study are indicated with bold text. **Table S3.** Average percent identity within *P. syringae* clade 2b-a clusters. **Table S4.** Protein sequence of cucurbit associated orthologous groups identified using Scoary. **Table S5.** Protein sequence of *P. syringae *clade unique 2b-a orthologous groups (OGs) identified using Scoary. **Table S6.** Protein sequence that corresponded to the difference in carbohydrate active enzymes (CAZYmes) family. **Table S7.** Regions of genome plasticity position in isolate 77-4C. Coding sequences were predicted using Prokka [2]. **Table S8.** *Pseudomonas syringae* strains used in this study. Isolates from this study are indicated with bold text. **Table S9.** Genes included in the phytotoxin database manually created for this study. **Table S10.** Genes in the siderophore database manually created for this study. **Table S11.** Ice nucleation protein sequences. **Figure S1.** Type III effector profile of *Pseudomonas syringae* phylogroup 2 isolated from *Cucurbitaceae*. Colour indicates copies of effector in the genome as shown in the key. Cluster and phylogroup are also indicated by colours described in the key. **Figure S2.** Disease severity rating scale. 0 = no symptom, 1 = leaf spot, 2 = necrotic lesions covering <25% of the leaf surface, 3 = necrotic lesions covering from 25 to 49% of the leaf surface, 4 = necrotic lesions covering from 50 to 74% and 5 = necrotic lesions covering ≥75% of the leaf surface. **Figure S3.** Representative of leaf symptoms in *Cucurbitaceae* hosts with spray inoculation of isolates 77-4C, KFR003-1 and KL004-k1. **Figure S4.** Comparison of RGP_3 DNA sequence in phylogroup 2. The lines indicate the presence of the region in the corresponding strain. **Figure S5.** Structural comparison of KL004-k1 hopZ1 (green) and KFR003-1 hopZ5 (blue). These protein structures were predicted using AlphaFold and compared using PyMOL. **Figure S6.** Mean disease severity rating of isolates 77-4C, KFR003-1 and KL004-k1 on cucurbit hosts. Isolates are identified by colour as shown in the key. ‘Eva’ and ‘Rosa’ are varieties of zucchini. The experiment was conducted in a growth cabinet. Five replicates were used for each isolate on each plant variety. Disease severity was rated from 0-5 which 0 = no symptom, 1 = leaf spots without necrotic lesions, 2 to 5 represented <25%, 25 to 49%, 50 to 74% and ≥75%, necrotic lesions covering the leaf surface, respectively. The vertical lines represent one standard error mean (0.376) calculated with GenStat. Significant differences are shown with a letter above the bar (LSD = 1.07, α = 0.05).

## Data Availability

The data generated in this article is available in the NCBI BioProject repository, accession number PRJNA779166.
