# Supplementary material for: Comparative genomics and genomic diversity of Pseudomonas syringae clade 2b-a in Australia
Source: BMC Microbiol. 2022 Nov 21;22:278. doi: 10.1186/s12866-022-02678-9 (PMC9677677; doi:10.1186/s12866-022-02678-9)
Supplement: Supplementary file 1 — Additional file 1: Table S1. Biolog results of isolate 77-4C, KFR003-1 and KL004-k1. Table S2. Assembly statistics of Pseudomonas syringae strains used in this study. Isolates from this study are indicated with bold text. Table S3. Average percent identity within P. syringae clade 2b-a clusters. Table S4. Protein sequence of cucurbit associated orthologous groups identified using Scoary. Table S5. Protein sequence of P. syringae clade unique 2b-a orthologous groups (OGs) identified using Scoary. Table S6. Protein sequence that corresponded to the difference in carbohydrate active enzymes (CAZYmes) family. Table S7. Regions of genome plasticity position in isolate 77-4C. Coding sequences were predicted using Prokka [2]. Table S8. Pseudomonas syringae strains used in this study. Isolates from this study are indicated with bold text. Table S9. Genes included in the phytotoxin database manually created for this study. Table S10. Genes in the siderophore database manually created for this study. Table S11. Ice nucleation protein sequences. Figure S1. Type III effector profile of Pseudomonas syringae phylogroup 2 isolated from Cucurbitaceae. Colour indicates copies of effector in the genome as shown in the key. Cluster and phylogroup are also indicated by colours described in the key. Figure S2. Disease severity rating scale. 0 = no symptom, 1 = leaf spot, 2 = necrotic lesions covering <25% of the leaf surface, 3 = necrotic lesions covering from 25 to 49% of the leaf surface, 4 = necrotic lesions covering from 50 to 74% and 5 = necrotic lesions covering ≥75% of the leaf surface. Figure S3. Representative of leaf symptoms in Cucurbitaceae hosts with spray inoculation of isolates 77-4C, KFR003-1 and KL004-k1. Figure S4. Comparison of RGP_3 DNA sequence in phylogroup 2. The lines indicate the presence of the region in the corresponding strain. Figure S5. Structural comparison of KL004-k1 hopZ1 (green) and KFR003-1 hopZ5 (blue). These protein structures were predicted using A [file 12866_2022_2678_MOESM1_ESM.docx]

**Table S1** Biolog results of isolate 77-4C, KFR003-1 and KL004-k1

| **Substrate** | **77-4C** | **KFR003-1** | **KL004-k1** |
| --- | --- | --- | --- |
| Negative Control | 0 | 0 | 0 |
| D-Raffinose | 0.027 | 0.01 | -0.009 |
| α-D-Glucose | 0.726 | 0.679 | 0.725 |
| D-Sorbitol | 0.693 | 0.594 | 0.353 |
| Gelatin | -0.026 | -0.011 | -0.004 |
| Pectin | 0.13 | 0.126 | 0.112 |
| p-Hydroxy-Phenylacetic Acid | -0.041 | -0.035 | -0.018 |
| Tween 40 | 0.132 | 0.155 | 0.129 |
| Dextrin | -0.013 | 0.004 | 0.007 |
| α-D-Lactose | -0.024 | -0.03 | -0.046 |
| D-Mannose | 0.75 | 0.729 | 0.404 |
| D-Mannitol | 0.75 | 0.849 | 0.561 |
| Glycyl-L-Proline | 0.019 | 0.063 | 0.066 |
| D-Galacturonic Acid | 0.789 | 0.959 | 0.759 |
| Methyl Pyruvate | 0.22 | 0.15 | 0.247 |
| γ-Amino-Butryric Acid | 1.013 | 0.472 | 0.591 |
| D-Maltose | -0.034 | 0.023 | -0.034 |
| D-Melibiose | -0.007 | -0.026 | -0.023 |
| D-Fructose | 0.424 | 0.557 | 0.346 |
| D-Arabitol | 0.63 | 0.744 | 0.431 |
| L-Alanine | 0.804 | 0.772 | 0.593 |
| L-Galactonic Acid Lactone | 0.417 | 0.417 | 0.496 |
| D-Lactic Acid Methyl Ester | -0.009 | -0.013 | 0.002 |
| β-Hydroxy-Butyric Acid | -0.018 | 0.009 | -0.016 |
| D-Trehalose | 0.009 | 0.034 | -0.021 |
| β-Methyl-D-Glucoside | 0.012 | -0.018 | -0.028 |
| D-Galactose | 0.698 | 0.651 | 0.374 |
| myo-Inositol | 0.858 | 0.934 | 0.806 |
| L-Arginine | 0.104 | 0.023 | 0.032 |
| D-Gluconic Acid | 0.95 | 1.095 | 0.956 |
| L-Lactic Acid | 0.892 | 0.653 | 0.665 |
| β-Hydroxy-D,L-Butyric Acid | -0.034 | -0.002 | 0.003 |
| D-Cellobiose | 0.009 | 0.061 | 0.01 |
| D-Salicin | 0.002 | -0.001 | 0.006 |
| 3-Methyl Glucose | 0.014 | -0.011 | 0.001 |
| Glycerol | 0.759 | 0.79 | 0.709 |
| L-Aspartic Acid | 0.906 | 0.854 | 0.872 |
| D-Glucuronic Acid | 0.954 | 1.028 | 0.937 |
| Citric Acid | 0.924 | 0.916 | 0.905 |
| α-Keto-Butyric Acid | -0.02 | -0.009 | -0.009 |
| Gentiobiose | 0.015 | 0.016 | -0.006 |
| N-Acetyl-D-Glucosamine | 0.019 | 0.005 | 0.011 |
| D-Fucose | 0.315 | 0.236 | 0.282 |
| D-Glucose-6-PO4 | 0.063 | 0.024 | 0.019 |
| L-Glutamic Acid | 1.038 | 0.978 | 1.002 |
| Glucuronamide | 0.145 | 0.113 | 0.154 |
| α-Keto-Glutaric Acid | 0.235 | 0.536 | 0.248 |
| Acetoacetic Acid | 0.031 | 0.011 | -0.025 |
| Sucrose | 0.877 | 0.8 | 0.754 |
| N-Acetyl-β-D-Mannosamine | -0.005 | -0.014 | -0.021 |
| L-Fucose | 0.075 | 0.027 | 0.026 |
| D-Fructose-6-PO4 | 0.062 | 0.02 | 0.032 |
| L-Histidine | 0.144 | 0.094 | 0.101 |
| Mucic Acid | 0.534 | 0.514 | 0.945 |
| D-Malic Acid | 0.853 | 0.887 | 0.681 |
| Propionic Acid | -0.044 | 0.053 | -0.05 |
| D-Turanose | 0.008 | -0.008 | -0.035 |
| N-Acetyl-D-Galactosamine | 0.019 | -0.024 | -0.028 |
| L-Rhamnose | 0.04 | -0.012 | -0.011 |
| D-Aspartic Acid | 0 | -0.032 | -0.025 |
| L-Pyroglutamic Acid | 0.059 | 0.018 | 0.02 |
| Quinic Acid | 1.05 | 1.133 | 1.011 |
| L-Malic Acid | 0.699 | 0.79 | 0.838 |
| Acetic Acid | 0.65 | 0.631 | 0.52 |
| Stachyose | 0.019 | 0.014 | -0.021 |
| N-Acetyl Neuraminic Acid | -0.007 | -0.023 | -0.046 |
| Inosine | 0.252 | 0.168 | 0.082 |
| D-Serine | 0.044 | 0.089 | 0.012 |
| L-Serine | 0.986 | 1.009 | 0.94 |
| D-Saccharic Acid | 0.906 | 0.98 | 0.93 |
| Bromo-Succinic Acid | 0.129 | 0.123 | 0.12 |
| Formic Acid | 0.284 | 0.353 | 0.267 |
| Positive Control | 1.151 | 1.221 | 1.196 |
| 1% NaCl | 1.014 | 0.974 | 0.908 |
| 1% Sodium Lactate | 1.207 | 1.276 | 1.324 |
| Troleandomycin | 0.049 | 0.118 | -0.072 |
| Lincomycin | 1.25 | 1.197 | 1.206 |
| Vancomycin | 1.319 | 1.287 | 1.258 |
| Nalidixic Acid | -0.067 | -0.052 | -0.066 |
| Aztreonam | -0.033 | -0.035 | -0.053 |
| pH 6 | 1.131 | 1.236 | 1.024 |
| 4% NaCl | 0.683 | 0.308 | 0.574 |
| Fusidic Acid | -0.066 | -0.061 | -0.05 |
| Rifamycin SV | 1.262 | 1.271 | 1.202 |
| Guanidine HCl | -0.077 | -0.066 | 0.337 |
| Tetrazolium Violet | 2.918 | 2.947 | 2.995 |
| Lithium Chloride | -0.097 | -0.092 | -0.09 |
| Sodium Butyrate | -0.103 | -0.095 | -0.098 |
| pH 5 | 1.276 | 1.129 | 0.856 |
| 8% NaCl | -0.007 | -0.011 | -0.001 |
| D-Serine | 2.633 | 2.756 | 2.657 |
| Minocycline | -0.089 | -0.08 | -0.086 |
| Niaproof 4 | 0.927 | 1.009 | 0.712 |
| Tetrazolium Blue | 2.575 | 2.791 | 2.688 |
| Potassium Tellurite | 1.293 | 1.292 | 1.148 |
| Sodium Bromate | -0.058 | -0.086 | -0.07 |

Red highlight: positive

Yellow highlight: borderline

No highlight: negative

**Table S2** Assembly statistics of *Pseudomonas syringae* strains used in this study

. Isolates from this study are indicated with bold text*.*

| **Strain** | **Phylogroup** | **Number of Contigs** | **Length (bases)** | **Number of coding sequence** | **%GC** | **Number of RGP** |
| --- | --- | --- | --- | --- | --- | --- |
| 03-19A | 2a | 74 | 6,151,226 | 5,345 | 59.14 | 54 |
| 13-139B | 2a | 116 | 6,391,564 | 5,634 | 59.11 | 60 |
| 13-429 | 2a | 114 | 6,277,920 | 5,504 | 59.12 | 52 |
| 200-1 | 2a | 125 | 6,157,703 | 5,412 | 59.11 | 55 |
| 31R1 | 2a | 1 | 5,874,625 | 5,040 | 59.31 | 37 |
| A2 | 2a | 23 | 5,901,107 | 5,139 | 59.23 | 51 |
| BRIP39023 | 2a | 34 | 5,938,198 | 5,132 | 59.23 | 48 |
| BS2121 | 2a | 112 | 6,245,995 | 5,473 | 59.08 | 54 |
| CFBP1754PT | 2a | 182 | 6,112,377 | 5,531 | 58.97 | 76 |
| ICMP11293 | 2a | 60 | 6,136,051 | 5,452 | 58.86 | 56 |
| NFACC10-1 | 2a | 21 | 5,827,168 | 4,989 | 59.37 | 47 |
| P66 | 2a | 27 | 6,185,625 | 5,334 | 59.08 | 48 |
| P73 | 2a | 48 | 6,140,398 | 5,314 | 59.13 | 45 |
| P77 | 2a | 45 | 6,133,764 | 5,296 | 59.14 | 42 |
| P87 | 2a | 48 | 6,141,769 | 5,303 | 59.13 | 45 |
| P89 | 2a | 101 | 6,222,478 | 5,424 | 59.08 | 54 |
| Pc58T | 2a | 7 | 6,345,256 | 5,757 | 58.78 | 54 |
| UMAF0158 | 2a | 2 | 5,850,990 | 5,049 | 59.28 | 42 |
| ZUM3984 | 2a | 87 | 6,255,982 | 5,455 | 59.06 | 51 |
| 1845 | 2b | 91 | 5,768,876 | 4,956 | 59.22 | 44 |
| 2507 | 2b | 97 | 5,944,881 | 5,139 | 59.1 | 54 |
| 41A | 2b | 24 | 5,983,849 | 5,136 | 59.11 | 54 |
| Alf3 | 2b | 29 | 5,812,264 | 4,986 | 59.18 | 45 |
| B64 | 2b | 1 | 5,930,035 | 5,079 | 59.12 | 42 |
| BRIP34881 | 2b | 96 | 6,017,073 | 5,214 | 58.9 | 58 |
| BS0292 | 2b | 75 | 6,227,170 | 5,434 | 58.84 | 63 |
| BS3827 | 2b | 28 | 6,076,522 | 5,256 | 59.01 | 49 |
| BS3829 | 2b | 38 | 5,969,077 | 5,193 | 59.05 | 54 |
| CRAFRU11 | 2b | 179 | 5,859,300 | 5,079 | 59.09 | 56 |
| HS191 | 2b | 2 | 6,002,759 | 5,159 | 58.96 | 47 |
| ICMP11168 | 2b | 97 | 5,966,977 | 5,211 | 59.13 | 61 |
| ICMP3023T | 2b | 179 | 6,073,080 | 5,300 | 58.97 | 58 |
| ICMP3947PT | 2b | 118 | 5,850,429 | 5,094 | 59.17 | 53 |
| ICMP4394PT | 2b | 248 | 6,012,246 | 5,294 | 58.99 | 62 |
| ICMP459PT | 2b | 149 | 5,977,364 | 5,237 | 58.77 | 58 |
| MB03 | 2b | 76 | 5,777,225 | 5,058 | 59.03 | 60 |
| P108 | 2b | 39 | 5,773,911 | 4,859 | 59.2 | 44 |
| Pla1188_1 | 2b | 117 | 6,048,138 | 5,320 | 58.91 | 57 |
| PP1 | 2b | 4 | 6,039,562 | 5,302 | 58.76 | 46 |
| PsyCC440 | 2b | 354 | 5,725,117 | 5,055 | 59.14 | 53 |
| SM | 2b | 2 | 6,124,102 | 5,355 | 59.02 | 46 |
| 13-140A | 2ba | 140 | 5,940,788 | 5,100 | 59.07 | 46 |
| 13-509A | 2ba | 145 | 5,911,528 | 5,101 | 59.06 | 52 |
| 13-C2 | 2ba | 122 | 5,935,634 | 5,088 | 59.06 | 49 |
| 14-32 | 2ba | 145 | 5,938,269 | 5,091 | 59.07 | 50 |
| 14-410 | 2ba | 179 | 5,940,226 | 5,167 | 59.07 | 52 |
| 14-Gil | 2ba | 234 | 5,930,843 | 5,179 | 59.07 | 56 |
| **77-4C** | **2ba** | **1** | **6,012,225** | **5,150** | **59.02** | **44** |
| **BRIP64883-a** | **2ba** | **70** | **5,885,794** | **5,056** | **59.08** | **46** |
| **BRIP65014-a** | **2ba** | **71** | **5,897,156** | **5,073** | **59.08** | **46** |
| **BRIP65014-b** | **2ba** | **70** | **5,897,341** | **5,067** | **59.08** | **46** |
| **BRIP65014-c** | **2ba** | **88** | **5,848,116** | **5,030** | **59.15** | **49** |
| **BRIP65014-d** | **2ba** | **66** | **5,897,085** | **5,073** | **59.07** | **46** |
| **BRIP65018-b** | **2ba** | **66** | **5,897,104** | **5,071** | **59.08** | **46** |
| **BRIP65018-d** | **2ba** | **128** | **6,070,384** | **5,252** | **59.01** | **54** |
| **BRIP65019-a** | **2ba** | **72** | **5,897,932** | **5,075** | **59.08** | **48** |
| CC457 | 2ba | 356 | 5,842,695 | 5,151 | 59.05 | 54 |
| **KFR003-1** | **2ba** | **128** | **5,911,433** | **5,086** | **59.1** | **59** |
| **KL004-k1** | **2ba** | **93** | **5,892,237** | **5,070** | **59.07** | **48** |
| P12831 | 2ba | 73 | 5,909,027 | 5,078 | 59.07 | 47 |
| P12855 | 2ba | 67 | 5,905,972 | 5,054 | 59.07 | 47 |
| P12857 | 2ba | 67 | 5,895,860 | 5,039 | 59.06 | 48 |
| P139 | 2ba | 66 | 5,909,662 | 5,065 | 59.07 | 49 |
| P22 | 2ba | 74 | 5,908,103 | 5,088 | 59.07 | 50 |
| P79 | 2ba | 71 | 5,910,782 | 5,086 | 59.07 | 48 |
| P84 | 2ba | 87 | 5,964,719 | 5,176 | 59.02 | 52 |
| P99 | 2ba | 50 | 5,908,799 | 5,059 | 59.07 | 44 |
| PS711 | 2ba | 129 | 5,904,410 | 5,132 | 59.06 | 53 |
| ZUM3584 | 2ba | 163 | 5,986,645 | 5,214 | 59.11 | 60 |
| 2339 | 2d | 68 | 6,122,357 | 5,232 | 59.22 | 57 |
| 2340 | 2d | 96 | 6,181,159 | 5,370 | 59.13 | 64 |
| ATCC10853PT | 2d | 196 | 6,285,198 | 5,588 | 59.15 | 75 |
| B301D | 2d | 1 | 6,094,819 | 5,240 | 59.16 | 46 |
| B728a | 2d | 1 | 6,093,698 | 5,200 | 59.23 | 47 |
| HRI-W7872 | 2d | 105 | 5,899,795 | 5,049 | 59.35 | 49 |
| HRI-W7924 | 2d | 130 | 6,239,649 | 5,442 | 59.11 | 63 |
| ICMP13102 | 2d | 158 | 5,954,747 | 5,187 | 59.32 | 55 |
| PD2774 | 2d | 180 | 6,357,389 | 5,608 | 59.17 | 62 |
| PsyCC94 | 2d | 566 | 6,149,858 | 5,513 | 59.26 | 77 |
| USA011 | 2d | 3 | 6,112,257 | 5,206 | 59.15 | 55 |
| CRAFRU12 | Pav | 246 | 5,933,130 | 5,164 | 59.37 | 68 |
| NCPPB4273T | Pav | 93 | 6,096,328 | 5,344 | 59.21 | 62 |
| Pav013 | Pav | 37 | 6,062,183 | 5,362 | 59.12 | 58 |
| Pav037 | Pav | 39 | 5,881,053 | 5,174 | 59.24 | 60 |

RGP: Regions of genome plasticity

**Table S3** Average percent identity within *P. syringae* clade 2b-a clusters

|  | Cluster A | Cluster B | Cluster C2 | Cluster C1 |
| --- | --- | --- | --- | --- |
| Cluster A | 99.98304 | 99.85726 | 99.14775 | 99.0493 |
| Cluster B | 99.85143 | 99.9449 | 99.06139 | 99.08196 |
| Cluster C2 | 99.08789 | 99.01051 | 99.95718 | 99.88573 |
| Cluster C1 | 99.00131 | 99.06555 | 99.90198 | 99.92497 |

**Table S4** Protein sequence of cucurbit associated orthologous groups identified using Scoary.

| **Orthologous group** | **Protein sequence** |
| --- | --- |
| group_2157^a^ | MSTASFRFGVMVGTALRECIRAVNQKPRPSAVQLVTAQCRRMPEPAMTHDECENYCSVPAIVRRKGINLHDWYEANTQEAQTKPVKKPRKSRASKVPEASKKQNAESSVKDPVMGSLDQLIA |
|  | MSSASFRFGMMVGTALRECIRAVNQKPRPSAVKVVSAQCRMMPEPAMTHDECENYCSVPAIVRRKGINLRDWYEANTQEAQTKQVKKARKSRASKVPETSKKRNAEPAVKAPVMGSLDQLIA |
| group_2159^b^ | MSITINHLPSTLLKLPVVLTPSAWKESVHLEQPSHIAEVGTRLGEVVLEAYRELHLQPDELQIDFGIYRFLPNGDRSGRHWLELRLHRMDAMNGNSYLCISLRAEQPLNLF |
|  | MSITINHLPSTLLKLPVVLTPSAWKESVHLEQPSHIAEIGTRLGEVVLEAYRELHLQPDEVQIDFGIYRFLPNGDRSGRHWLELKLHRIDAINGSPYLCISLRAEQPLNLF |
| group_3364^c^ | MTFKRAILLPITISSFNRRYGNADVKVEVSNGGDLDGSISYAYFQPLSEQKGKPPTKEGSTRP |
| shcA | MSSLFYKTLLDDFSRCMEMPALAFDDKGACNMIIDKAFALTLLRDDTHQRLLLIGLLEPHEDLPLQRLLAGALNPLVNAGPGIGWDEQSGLYHAYQSIPREKVSVEMLKLEIAGLVEWMKCWREART |
| hopA1 | MNPIHARFSSVEALRHSNVDIQAIKSEGQLEVNGKRYEIRAAADGSIAVLRPDQQSKADKFFKGAAHLIGGQSQRAQIAQVLNEKAAAVPRLDRMLGRRFDLEKGGSSAVGAAIKAADSRLTSKQTFASFQQWAEKAEALGRDTEIGIYMIYKRDTPDTTPMNAAEQEHYLETLQALDNKKNLIIRPQIHDDREEEELDLGRYIAEDRNARTGFFRMVPKDQRAPETNSGRLTIGVEPKYGAQLALAMATLMDKHKSVTQGKVVGPAKYGQQTDSAILYINGDLAKAVKLGEKLKKLSGIPPEGFVEHTPLSMQSTGLGLSYAESVEGQPSSHGQARTHVIMDALKGQGPMENRLKMALAERGYDPENPALRARN |
| vgrG | MIFNALEQLTQQNSRLITFDSPLPPEQELRLENFSGREAISELFAFEATLISSDARIELKKLVGKKVSLGLTLADGNVRHLNAHVSDFVHLGADGGVASYHARLVPWVWLLSRRSDSRVFQDKTVEEVVREIFSYYQSLAVFEFRLTRPLKKMSYCTQYMESDLNFILRLLEAEGLFFTFQHSQDEHCMMIMDDSRDLPALPAQPVIRYHRASVTEEADSITTWSSSRNLQPTQLAMKSFDYKQPRDPQLVQMKSIAKQGEVGEFETFDYEGVYGYTDLEDGQQKARNRLEAMEVLGKQFSGSSNCRVMEPGYFFELTQHYDHDQDSQEDRQFLLLSVNHSGQNNYLSHGEPGYQNSFECIRRKIPYRHRLSTPRAAIRGPQTAIIVGPPGEEIYTDEMGRVKVQFHWDRYGQFDDKSSCWVRVAQSGASGGFGSIQIPRVGDEVVVVFLDGNPDRPLIMGSLYNSQNTPPWSLPANKTQSGFLTRSMKGHGGTANFFRFEDKAGAEQVIMHAERNMDTEIEFDETHKVGNNRLMTIDGMQTEIIKKDAVMNVQEGSLTIQVDNQFIQVNAKQHIILQVGESSITLTPDGIEIKGNAITTVSKGTTQITGAAVRVND |

Sequence based on isolate 77-4C

^a^ two coding sequences in isolate 77-4C

^d^ Locus tag Ga0170668_1061120 in [1]

^e^ Locus tag Ga0170668_106332 in [1]

**Table S5** Protein sequence of *P. syringae* clade unique 2b-a orthologous groups (OGs) identified using Scoary.

| **OGs** | **Protein sequence** |
| --- | --- |
| ycf3 | MLERKALLDEIATHFAVLKFFIETNTASGFVDTNIVSEDFVASILNVSHKWNLANTNHTTTNAKSIDLLDESNRIGVQVSSRVDSKKINSTINSLIENEHRTKIGELFFFSLKQKQTRYKVDAECPGIKFTTKNVLDFNQVIRKTKALSTGVDDLRQMRDVVIKNFSEIPRRIIEKATTISNWLSHFDDSIVDKKLEELLGRSFQTGETLKAQVILLIEKNELIQAKELLDSLANTSRKMAAQDFIDIANLYTLLGSDQADIYYSHAASLDPESVKEANLYAIGLMLRGKLPEAEKVFKACLEKEDITLKEREAVLGNLGFLYKNNGRFSESIDAFEKALVISDSIDHKIGRIKHINGLGSCYLNLENLELSDQYFSQAKSLLDDTLKTSKDDSEKKELRSIKSNLLTNIAIRLKHMAEISGDESLFHQALEFLDQAVDLAEMMGDSRSLLRHYGNIANIYQGLKNLEKSREYNVKAYELSIQEEDKRSQVASLLNIGLVDKDEGDLLSAKSNFKLALKLENNVYPKIRANLLASSALVHKALGEHSDAQEHYDQAEKLYKELGLQDSLYSLEVRFSQVCAAGS |
| group_7141 | MNPTHRSEAEVFKELINLTARPGYAHAIAGICHRDNFVSFQGEHKPSDLDHIFDRKRLNRNEISTVLGLMMRQPLDLSEVDEATLRGYASRTDQLLGELHSALNGQVIVELAPQAQKGEVVEDFWQGPMMKEPIFYATESAYSFQYRDFFAEKHAPDDAWLERVMGFSSAQALRVANAMCSLMNERANNGARLTGEPQLGQMPTGSVLELFEFRAHEVAHCSGQELDVVEAVFKALTFSGDNGLFREVGDFNQVAATPLLPTGRDSVLLFSHHMIYEALYESPFFWMNQDKDYKQQASDNRGAFTELFSLRRLASVFGRSNVHTNVNLFEGKRIVAEADVLVIFGDRIIIVQAKAKKLTLAARKGSDGHIKADFAAAIQKAHDQGAECATAIRSGSCRLEDDQGQAVVLPKQVKEIYPFCVVSDHYPALAFQASQYLKYSADEVVRPPLVMDVFLLDVLAEMLDTPLRFLSYVRMRSESSSKLMAGHELTTLAYHLRCNLWLDDQFNMVMLEDSIAADLDTAMVVRREGLPGQRVPEGILTRMKGTYYERLIEQIERRPNPAILELGFTLLSMSGETCDAVHQAIERLTNMTKIDGKRHDFTIGMTEHGAGICFHCNPAPSQEAIRTLEVHCAKRKYTHQTPEWYGVSVGVNGEIQFGITLNFPWEKSTPLDDLTREMKPSSSVRQGLKAMERLFVPKKHGRNDACPCGSGLKYKKCCL |
| group_7142 | MRTALHRDTLETLDAQGYEDAYGKPVDNNGKERVRPPALCDICRQDVFLRAEHSKLRTPNFVHFEDSAPCPVKAFNAQAYRALNPEQGNAADAQRLREGFFATWRYHWREFDRIIGYASVFDFVSVLKYADRHGIWRYRDMGVQDVLPVLLTLMDFPPLPKGKRHLRDYGLRFLYVGTVASTNDYWNLPAHERRLLKVVYEFPGQARTFQSERISAKHPVDFEPDYEAARFDLDVDNQPTAHPYVVNTMAREFKV |
| rep_2 | MNPFDRARIQAINARAKLIAACGEQYPTSQQLIDVAEATLGIAIEWVSPDDYALGGADALLQRSMRTILIRDDVRQEAKAYLLAHELGHWYLDSPQPAPGTSTVSFIADPDDEADTTAATSNPSSTSAGSAVVEGYGAWERNELQANVFARELLLPRTAARALWSTGWRSRLIATHFLLPLEVVRQQLADALLLPDVPPVPAAPAHPPSDAQRLAAEAPEKFVNVVAGPGTGKTTTLVHRVAHLIASGVPANRILVMTFTNRAAQELVERLTAANVPNASQVWAGTFHSFGLELLRKHHLLFGLTSQIKIADALQQVRMMLCAVRDFFRQGGAGHDIPEAFDAEPDPYSGTLNNFSCRFWQPA |
| group_7144 | MKRNVNLPSPSLNAPLRAWSDYLGWVSATADFQVVGGVCRCTVTMPEGQILEVQAATPDLLAKQVMADLTANMVVPPTMDRSTTSTLQDAPKQPKQHQKQQQQCEPGQDEPGPVIPEGLQTQASPQEVSMRSHYEPALPRASQAPRSASVSTTKGTLQTVGVYVPVAMHEVLKAVAEEQGKKFSVLVREMVERGYERFEDAIEERSGRKVLDGYEQKVASYDGQSEQWMARLDRKLSLELKLTAKEYGRSASQIVGGLLAEELSHCPQAQAVSTTAQAVATTAARVIADPDEVVRAQAAIAGLVGPKANELANHLGLPGKRRLVNEVLVGIVRAPGVLMRDVAEYFQVSPEAMNEAVSLNFRQLAAPSFKAPNGQPRVLTEPVSWEEAVKALHLPHDEEAQLLGLGD |
| group_7145 | MHDNSLESCLAALDNPVDLGAYLLPQVIAALTAVDVDLYDVQRSAWTNPLIHLRAVFDIHDLPDTPTAFIPVPDVASTVAARASAVVCLAALNSDTISYGSENDGHLFVNLVVLPGDGDFADKSKSNMRGHTDGVSFPVRGQPHEFDQRVAPSPDFVCLSGLRNPNSTATTVMPLSAVLQKLTDEEIEELTKPQYILRPQKTFKVGLRKLLGKDSPLLEVMVDIQLLFETQDGHWIRYSQSAADTDFDSELANQAIKSFEQACQECSASVVIAPGDILLVNNRVGLHGRGVVGGEAGGDSRWLLRTYGLDTRELRAEQRYPDSTFKLYP |
| group_7146 | MESPSTPLALLPEQNLKPLEPMPASTELSPTSPEAHQAQQPRQAPQPHQTPHPQPEGASALSLALHEGLMYQRTLPREERTTHLGVFAYERLDAITRVIEQHLPSLKPDLSTLLNLLTVASQTTLTPEQAIELRGIYNRTHEKVFPVNEKYLYWCHQFHGLSEDHSLRSISWDHLKRLEEESYADDELKALVQELKLSFGRVKDVSDSQGFRQVFEYYGEALVYSKLSKHFKTKRIVARQDSMPDFVCVLPNGKTFYVELKSLDLVQAPLRSDQMHEDAMNQNIEIEDQILQGNKVAMAEREIAPFKPPFDDGSYDPRSLLLVINTLMKKARGAFKESQFAQGPTFAFMLCDRLMIPGGNHSVAPQYFERGGDAVVSGALWNACFAKMGWPVFRMPDFKGAPGLEGHMPEHGLFVDEYVKFPTGAVVFSEFGWQEDTLMGLYDSTWRPNSDWTVEDTEGVIHVLCTAYNDERNSYGQSVAMT |
| ravA | MSASLDLAASQIRHAVRAVTEGLVGREQLAELIVLAAVAQEHILVVGPPGTAKSAVVRRVAQSMGGRYFEYLLGRFTEPSELFGTVDLQKLREGTVETDISGMLPEADIVFLDEVFLGSTAILNTLLGVLNERRFRRGHTQIQCPLRVCVAAANGVPDDESLAAFGDRFLLHLFVDAVPDNQLEAMLAGGWQSEQRPVPQLLGLTQLDKLGLALKDVDLSLARPALAQAIRRLREAGIQLSDRRIVKSQRLIAAAALLRGQLEASEADLWPLLYVLPTRETQQHGREVLQDLFTQCSNSHLFSAVEEATLQPMARLHRLIETAEDYLARSEPPASLLLEALLREIDANFNSQTIPQRLHEARGQVAHLLTTRA |
| group_7115 | MTNSVTAAAIDWNWRVRREPGQPQAAVAWGEVAARLHARLLRMPEDQTAQLQATANCDVLIVTGNADDLPWVEGVDYACTEPAAPGLWLPTSWEPDAPLDLMGQALLGRFARTPLLLWHAPKVVVPLDRCLPLTARHLQRIQNRWAGR |
| group_7116 | MQLPQSLRPWRDWLQWFPPEQLPLLADLFARLNPLLGPLRGMQQGGVPEPDGLGDLHRRGPYERLLASEWLLADELPDEFLRRAVVGEHLFLAPQYRTHQANRMIVVLFDAGPLQLGGPRLVHLALLILLARRASEAGAELRWGSLQNAPQLHEFKDAAHLKQLLDARTYQTVTDEHWHTWRAWLSEQNYDSGERWLVGQQLPATNARSCTHRVQIQRSLDGSSLMVALQDAGTRQVALPNPDERLALQLIKGEFDIARQITRTAVKTLIPRVALTLPPVIAGSGSHVALKLLDEPGLVVIKLPAPRQKKPFDVRRTLWNNRSTPLAITFPGRMPGAILSLDEQLVFWNMPGVKPVARPERERLQMPVGTATLLPMVWMHNGTYGRVFLLDTKGHLAFWVVENGKLPTQHQSGVTHSMADKVVGMAQVDRNILAYLRHDAGRLYVHRINPWISQSKGHVVGTSKGVNVEQVLFPASACWDRAFHGCAWMRLVDGQQQWQMVAPDLQTEQIDLAPGWKGLGLLIGEDEVLSMVLLSPNQQTVALYFQGEQRVLFTTNETIARISFCPMSGLVAALTKARELVVYSARDERRVLQVMCNQAQTQSEDAPHVRT |
| group_7117 | MSGPDAVIIRHPALSGYQPIQALWFPIERFSEQERARLILTHWQTDARAYRFAEGDLLRFPKAHTMLCEGLAGWPLIREGRTLCSARLSPEEMQRLAHADVWLVRGTQVNALHLNDATALTPDQWIDVSQYALLDTYDCQDVLPEQQVEPQAVQTDIREILGDSLAPVDPEREAVMQALLERQRQVAPAGVAAARHAPGHANSPQSAGPLSDGLPGFIAVAVGIGLYIIHSLGSPQLPTATSAAERAKPPRSVDPSTFDLGSVFVWTVIGAALFTLVLVAVRAWMRREGTHSAAINTPPAVTANAARPSKPPVPPRASGQPRGPARWRRWLTLLTQNSRLSALYGKRQVAYMQRMLEMFENGDLEEALRHAIPLGGGQGNAGQAFGTPNRREDLTLTQHSGPRTSMLFEADLETHLKQVYRQSFERLDREGRIEEAVFVLAELLRARQEALDYLEKHQRYQQAADLALAWGMPTSAIVRLLCLAGNWQRALQVARRDNVFADTVILLEQNWPEAAARLRLEWALALTEKGFWLQAVDVVWSLPTEHERATQWLLAAEGAGGSLAIGALVKRAALLPDTLSTYATWVEKLRNDPERVVERAALAQALLAHKTHSPALAWLAAATVRAMICDQAGPHAHLTQNQLQALVKMSKDKLLQADLPGSALPKNKLLNQSLERASAALSFTAPERGNRAICDAVPLTDGRYLMALGEAGASIVEASGKTAFHFPVPAHTIVLGHSRQVALALARRGDVWRICKLDLVNRSATDLGVLMLDAFAKNFDGTTWTVGRGSQLRVVDVDRGFETLWHVSDLPGQVAFIREDERNESVLLNTSHNFQLWHYRLPDRRLLRREPVPERRQEHSWQVYNAAGRTTELWIEHETGSDPLLMSYEGGTSKCYTLPGLIQESADPLNVYSFEQWLVVCYATGENEARWHFINRNTDRLCAILHWPLHHAQLRCVGNEWILFDHQGRLWHIDTTRSITRALSVQ |
| group_7138 | MSFPSYPTYKDSGVEWLGEVPQSWSVYSIKRTVDGCINGLWGDEPDGENDIAVIRVADFERSFSTVGLDKLTYRSITPKERQSRLIKSGDLLIEKSGGGEKTLVGCVVLFTHEFDAITSNFVARMRPLAEFDSQFLCYAFGNLYHGRVNYPSVKQVTGIQNLDAESYLQERFCFPTRVEQTQIARFLNHETARIDALIEEQQRLIELLKEKRQAVISHAVTKGLDPTVPMKDSGVKWLGEVPAHWKVSKLGHYAQILTGFPFPSASFSHDESDVRLLRGANIGVGSLKWIDTVYWSLSEGDSLSSYLMGEGQIVLGMDRPWISEGMRIARVTKEDMPCLLLQRVAAISPSAELDDEYLFCLLASELFKAYVEPDLTGVSVPHISPEQIMSFQISVPEIGEQRRISSFIRNQLDQMAALFKQASESVELLQERRSALISAAVTGKIDVRGWQPPASVQTPELEREAV |
| group_7206 | MITYEYALERAKEYLKDSEIALQLTHEGEFSEGWFF |
| group_7088 | MQIISIFNNKGGVGKTTYIYHIAHLLERAGQTVLLVDLDSQCNLSSYCLSDQELERSWRSERGNSIWNAIERVSVGMGDIRERRPTRLIKQHALGEYENLYLVPGDVMLSSYEDRLGDTWAAARGGDPMALRVQSAIYRYILWCAETIQADVVLLDLGPNLGSLNRAVLAASNYFITPVSPDLFSIRGTENLGSKLNTWRTGWDQCNTYADYIDFQLPAGRPCFLGYVKQQHNVRDNEAGMTRGWNLFGEQIEGAVRQNIVSRLEPLGQVFHWGDGLFDLGGIPNLHSLVPYSQEARKPIFDLRSADGLTGAHISRARNSAEYFEPMVDVLLQVLD |
| group_7089 | MATLSPEIKTFTARALACFDSPSQVAEVVKVNFGVTVSRQQVDIRAPTKRSSKGLAKRWVTLFEDTRASFRETMVEVPVANRAYRLHALGRMLEEAERRDILKLLEQAAKECGDIYNSAQVKQMTGQANSPHRCGEFVLAPLGAPRQQVGTGRLVPLVISPHPALQAGRPICATISYAAISWRCSRKNKLS |
| group_7091 | MKHTITIDIGNDVSDIPTMESMTPEEYSSYVEEALFWVDHHDVLRSMVGEYPIATSAEQIELLITYLKDRADDLRRAGH |
| group_7092 | MSDEEVVMEDRFERWLKVSIGMARFEPHLLDLVQDMGQLDANLCSMDARFVRAHPDGQNALYDHYSIQSHKTQSYLWVLGAYEILRTLAQRVKDGQSDDPPDVAARIEAARDRFARVRIPLAKFEASRKYKAIDNPIAYPGLDLKYGIAWQLNESEVISRQELSDVFLEALEFVRAAKLGRQAKP |
| group_7179 | MRYMTIRKFASESGYSEDAIRSKIRDGIWRLGEIWYRAPDGRTLIDMEGYESWVETGGEFGRSPIRASKSRSCIGASGAAAHHAQALHH |
| group_7180 | MVYKEWMRVQIPPATFILKTPKGVFFDLKFSEY |
| group_3605_1 | MKIVSMHVQNYKTLENIVIPFAETFNSISGKNNAGKTSVITALRGVLKDKGKENWWFGNQDIEYSSSLTQWTKAGTPIVLTYILKFSEAEDPGMYHFVIKIASLQNFDNREFLLKIELNHTDSGRNAEVYINEKKT |
| group_3605_2 | MQKSISTKKKLDAFTSKEIYTKITGTSLALYYHSAEVGLSGFGGGGISRLTQDLMFTNEERADLSADQEKLKKKIKKLAGKNRTGLSDLLGKLEDKYDVELSIFEKYLSGHIPLEINLKDKSVDIPLSAWGTGTQNRTQVMMLILYANKIKEDMGGVNRITPIVIIEEPESFLHPSAQAEFGRIIRNLSRDLQIQIVTSTHSPYMLCQEHPESNILLDRKLYRKSLRQTEIVPVSEENWMSPFSNILGLANDSFSSWHAVIEAKKDCAIFVEGIIDKQYIEFIASLGVTGFSLPTGCEILPYEGKDALRNSILLKFVLEKFHKYLLTFDLDALNELDRPMRTLGLERDKHYHPIGISEPGKDCIEGLLPGEIPASVYSSNYSLVMKAQSQDPKDRKSAKNSLKALMLDAFKKRSDWTKNDLAGFKPLFSNITQAFKE |
| group_7174 | MKNDFKFNTHACSINDLNPKCRQLWSIGQQVAVRRLSVQTRAAYQQPPRYQLISDFSTRAARIAGNYARIYLELEPKGKPELKGRFYWTGLAAFASKQVMCALEYTTHTKMRAVPLMDLSLDLSKQFLGMGNFGFFKIFLFGIGFI |
| group_7200 | MSDEKKERSFLATLDLKDGTKLGFSKIAAGGALEEVDELCANSRLIRSIKIYFQPNDEGDYVMFFKGFAAPDKIPPKTNAPIEDALKAQVERQNKQLEERVESYSPSGADSDTYRASLSVSDDGTPPPENQITASQAPEQEPPIYLAVSEGGWVHKSEESGYAYLAGIADCGGSKFSIDRTDAQEVYLKSWRSKNFMQTYKREDFPDKAWFAWMTDCDGTDAVLKLKIIERYNADGSVKA |

Protein sequence based on 77-4C

Yellow highlight: not in regions of genome plasticity

Blue highlight: 2 coding sequences in 77-4C, BRIP65014-c, BRIP65018-d and ZUM3584

**Table S6** Protein sequence that corresponded to the difference in carbohydrate active enzymes (CAZYmes) family

| CAZymes family | KEGG orthology | KEGG Pathway | Protein sequence |
| --- | --- | --- | --- |
| GH19^a^ | putative chitinase | - | MPITAQQLLQILPNAGQRAGVFAPVLNTAMSKYQIVTPLRIAAFIAQVGHESGQLRYVRELGGSAYLSKYDTGKLAERLGNTPEADGDGQLYRGRGLIQVTGRANYEACAEALGLDLINHPELLEQPDHAAMSAAWFWDRANLNALADKGDFLTITKRINGGTNGLADRQALYARALEVLA |
| GH24^b^ | lysozyme | - | MSLRVKITAGLLLLCSGTLTAFLGTWEGNGQNVVYADKLASGLPTVCKGITKHTSPDPVLVGEYWSDARCAEVEGLVIAKGQLSLADCLTNQVIGQNTFDALSSHGHNFGVPTTCASRAVGLINAGRIAEGCKALAWASDGTTPVWAYVTGADGRKTFVRGLHNRRLAEMRLCLQ |
| GT2^a^ | - | - | MAPLRVACVIPTYNGRKDLERLLDSLAAQTASFDTLIVDSSSSDGTLELAQSRCANVLRIDSKDFNHGGTRQMMVDLHPDYDVYVFMTQDAYVEDINAIANLLLPFADPKVGAVCGRQLPHKDANLLAQHARLFNYPPTSQVKTLADAGTLGIKTPFMSNSFAAYRGEALRAIGGFPRHVILSEDMYVTAKMLIDGWKAAYEGSAVCRHSHNYSLREEFRRYFDIGVFQAREAWIYETFGGIGGEGMRYVKSELKFLGPRRILWWPVSFVRNALKLLAYKLSRQEKRLPRGVKKKLGMYARYWDSPYA |
| GT4^a^ | polysaccharide biosynthesis protein PslI | Biofilm formation | MRIGLDYRAAAGYPTSGIGRQNLALERAFREHPDVQLQLFGVAPYDHPVRRLMHAPRWAAPLNSVHRLPDRLRFEGQFLPAALRDAGIQIYMANINMGLPLGRKPADMRYVLQLHDLFQLTQHNSHGSRLKARIYRMTDYLSIAWSLKVADQIWVPSQFTANEAARMFPRIKAKLRVIPLLVEAFKGEPADIAQLRLPLRYWLCVGTREPRKNIKWFVDAWQTARMQFVDTPELVLVGGDDPLTEAQRQLPGLHVLSGLSDAELHAVYKNAERLWQPSKSEGFGLPVIEALNSGTPVAVASGSSLDEVAPPDSPRFSPVDSGNLIRLMGTLSQAPEEDPAIATDWTQRYQLKAFCERVHVALEELR |

^a^ Sequence based on isolate 77-4C

^b^ Sequence based on isolate KL004-k1

**Table S7** Regions of genome plasticity position in isolate 77-4C

. Coding sequences were predicted using Prokka [2]

| **RGP name** | **RGP start** | **RGP end** | **RGP length** | **Locus tag** | **Gene start** | **Gene end** | **COG category** | **KEGG number** | **Note** |
| --- | --- | --- | --- | --- | --- | --- | --- | --- | --- |
| RGP_22 | 24405 | 33629 | 9224 |  | 24405 | 24633 | - |  |  |
|  |  |  |  |  | 24681 | 25071 | - |  |  |
|  |  |  |  |  | 25213 | 25411 | - |  |  |
|  |  |  |  | LPB74_00115 | 25475 | 25841 | - |  |  |
|  |  |  |  | LPB74_00120 | 26166 | 26934 | T | K20074 |  |
|  |  |  |  | LPB74_00125 | 26930 | 27689 | KLT | K12132 |  |
|  |  |  |  | LPB74_00130 | 28093 | 29806 | L | K03657 |  |
|  |  |  |  | LPB74_00135 | 29802 | 31701 | L | K07459 |  |
|  |  |  |  | LPB74_00145 | 33002 | 33629 | K |  |  |
| RGP_30 | 43834 | 51427 | 7593 | LPB74_00205 | 43834 | 44749 | K |  |  |
|  |  |  |  | LPB74_00210 | 44860 | 45718 | G |  |  |
|  |  |  |  | LPB74_00225 | 46348 | 46561 | Q |  |  |
|  |  |  |  | LPB74_00230 | 46831 | 47188 | - |  |  |
|  |  |  |  | LPB74_00235 | 47204 | 47645 | S |  |  |
|  |  |  |  | LPB74_00240 | 47973 | 48339 | - |  |  |
|  |  |  |  | LPB74_00245 | 48468 | 48768 | - |  |  |
|  |  |  |  | LPB74_00250 | 48897 | 49788 | - |  |  |
|  |  |  |  | LPB74_00255 | 50403 | 51078 | S |  |  |
|  |  |  |  | LPB74_00260 | 51178 | 51427 | - |  |  |
| RGP_37 | 254111 | 258144 | 4033 | LPB74_01230 | 254111 | 254729 | S |  |  |
|  |  |  |  | LPB74_01235 | 254783 | 255401 | S |  |  |
|  |  |  |  | LPB74_01240 | 255455 | 256073 | S |  |  |
|  |  |  |  | LPB74_01245 | 256127 | 256745 | S |  |  |
|  |  |  |  | LPB74_01250 | 256755 | 258144 | S |  |  |
| RGP_13 | 617487 | 633773 | 16286 |  | 617487 | 619779 | S |  |  |
|  |  |  |  | LPB74_02710 | 619778 | 620630 | S |  |  |
|  |  |  |  |  | 620661 | 620961 | K |  |  |
|  |  |  |  | LPB74_02725 | 621321 | 621684 | - |  |  |
|  |  |  |  | LPB74_02730 | 621976 | 623056 | S |  |  |
|  |  |  |  | LPB74_02745 | 623750 | 624518 | - |  |  |
|  |  |  |  |  | 624840 | 625275 | V | K07448 |  |
|  |  |  |  | LPB74_02755 | 625509 | 625740 | L |  |  |
|  |  |  |  |  | 625749 | 628047 | L | K01153 |  |
|  |  |  |  |  | 628102 | 628642 | L | K07483 |  |
|  |  |  |  |  | 628848 | 629478 | L | K07497 |  |
|  |  |  |  |  | 629449 | 630400 | L | K01153 |  |
|  |  |  |  | LPB74_02775 | 630399 | 631800 | V | K01154 | Clade 2b-a OGs |
|  |  |  |  | LPB74_02780 | 631796 | 633773 | V | K03427 |  |
| RGP_43 | 706862 | 711189 | 4327 | LPB74_03100 | 706862 | 707267 | K |  |  |
|  |  |  |  |  | 708238 | 708940 | S |  |  |
|  |  |  |  | LPB74_03115 | 709197 | 710565 | S |  |  |
|  |  |  |  | LPB74_03120 | 710580 | 711189 | S |  |  |
| RGP_0 | 754281 | 847159 | 92878 | LPB74_03340 | 754281 | 755685 | L |  |  |
|  |  |  |  | LPB74_03345 | 755681 | 757607 | S |  |  |
|  |  |  |  | LPB74_03350 | 758035 | 758728 | E |  |  |
|  |  |  |  | LPB74_03355 | 758841 | 759276 | KT | K03503 |  |
|  |  |  |  | LPB74_03360 | 759262 | 760543 | L | K03502 |  |
|  |  |  |  |  | 760762 | 761152 | S |  |  |
|  |  |  |  | LPB74_03370 | 761203 | 762742 | S |  |  |
|  |  |  |  | LPB74_03375 | 762738 | 763113 | - |  |  |
|  |  |  |  |  | 763115 | 764483 | S |  |  |
|  |  |  |  | LPB74_03385 | 764503 | 765442 | S |  |  |
|  |  |  |  |  | 765438 | 765912 | S |  |  |
|  |  |  |  | LPB74_03395 | 766201 | 766453 | S |  |  |
|  |  |  |  |  | 766823 | 767087 | - |  |  |
|  |  |  |  | LPB74_03410 | 767207 | 767396 | - |  |  |
|  |  |  |  | LPB74_03415 | 767474 | 767726 | - |  |  |
|  |  |  |  | LPB74_03420 | 768000 | 769416 | L | K00558 |  |
|  |  |  |  | LPB74_03425 | 769755 | 770337 | - |  |  |
|  |  |  |  | LPB74_03430 | 770337 | 771027 | O |  |  |
|  |  |  |  | LPB74_03435 | 771023 | 771323 | - |  |  |
|  |  |  |  | LPB74_03440 | 771319 | 774328 | U |  |  |
|  |  |  |  | LPB74_03445 | 774327 | 774783 | S |  |  |
|  |  |  |  | LPB74_03450 | 774760 | 776245 | M |  |  |
|  |  |  |  | LPB74_03455 | 776234 | 777158 | NU |  |  |
|  |  |  |  | LPB74_03460 | 777154 | 777823 | S |  |  |
|  |  |  |  | LPB74_03465 | 777819 | 778212 | S |  |  |
|  |  |  |  | LPB74_03470 | 778227 | 778596 | S |  |  |
|  |  |  |  | LPB74_03475 | 778616 | 778856 | S |  |  |
|  |  |  |  | LPB74_03480 | 778852 | 779215 | S |  |  |
|  |  |  |  |  | 779294 | 779669 | - |  |  |
|  |  |  |  | LPB74_03490 | 780096 | 781389 | EGP | K08217 |  |
|  |  |  |  | LPB74_03500 | 782332 | 783433 | E | K00613 |  |
|  |  |  |  | LPB74_03505 | 783519 | 784776 | M |  |  |
|  |  |  |  | LPB74_03510 | 784828 | 786073 | M |  |  |
|  |  |  |  | LPB74_03515 | 786238 | 787675 | L |  |  |
|  |  |  |  | LPB74_03520 | 787684 | 788434 | S |  |  |
|  |  |  |  | LPB74_03525 | 788473 | 790633 | U |  |  |
|  |  |  |  | LPB74_03530 | 790644 | 791151 | KT |  |  |
|  |  |  |  | LPB74_03535 | 791147 | 791705 | M |  |  |
|  |  |  |  | LPB74_03540 | 791689 | 792436 | O |  |  |
|  |  |  |  | LPB74_03545 | 792444 | 793155 | - |  |  |
|  |  |  |  | LPB74_03550 | 793389 | 793839 | - |  |  |
|  |  |  |  | LPB74_03555 | 794178 | 796443 | L |  |  |
|  |  |  |  | LPB74_03560 | 796549 | 798001 | L |  |  |
|  |  |  |  | LPB74_03565 | 798029 | 798629 | - |  |  |
|  |  |  |  | LPB74_03570 | 798695 | 799220 | - |  |  |
|  |  |  |  | LPB74_03575 | 799308 | 799920 | S |  |  |
|  |  |  |  | LPB74_03580 | 800015 | 800351 | - |  |  |
|  |  |  |  | LPB74_03585 | 800439 | 800757 | - |  |  |
|  |  |  |  | LPB74_03590 | 800935 | 801916 | L | K07481 |  |
|  |  |  |  | LPB74_03595 | 802021 | 802177 | - |  |  |
|  |  |  |  | LPB74_03600 | 802247 | 802820 | S |  |  |
|  |  |  |  | LPB74_03605 | 802916 | 803147 | - |  |  |
|  |  |  |  | LPB74_03610 | 803452 | 803821 | - |  |  |
|  |  |  |  | LPB74_03615 | 804005 | 804539 | - |  |  |
|  |  |  |  | LPB74_03620 | 804886 | 805153 | - |  |  |
|  |  |  |  | LPB74_03625 | 805974 | 807996 | L | K03169 |  |
|  |  |  |  |  | 808006 | 808150 | - |  |  |
|  |  |  |  |  | 808805 | 809351 | - |  |  |
|  |  |  |  | LPB74_03640 | 809347 | 810307 | L |  |  |
|  |  |  |  | LPB74_03645 | 810331 | 810556 | - |  |  |
|  |  |  |  | LPB74_03650 | 810574 | 811015 | S |  |  |
|  |  |  |  | LPB74_03655 | 811046 | 812453 | NU |  |  |
|  |  |  |  | LPB74_03660 | 812449 | 813382 | NU |  |  |
|  |  |  |  | LPB74_03665 | 813384 | 813909 | S |  |  |
|  |  |  |  | LPB74_03670 | 813947 | 815039 | NU |  |  |
|  |  |  |  | LPB74_03675 | 815028 | 816660 | NU |  |  |
|  |  |  |  | LPB74_03680 | 816656 | 817259 | - |  |  |
|  |  |  |  | LPB74_03685 | 817248 | 818607 | S |  |  |
|  |  |  |  | LPB74_03690 | 818608 | 820345 | NU |  |  |
|  |  |  |  | LPB74_03695 | 820341 | 821475 | S |  |  |
|  |  |  |  | LPB74_03700 | 821679 | 821928 | - |  |  |
|  |  |  |  | LPB74_03705 | 822027 | 823980 | KL |  |  |
|  |  |  |  | LPB74_03710 | 823982 | 825869 | - |  |  |
|  |  |  |  |  | 826144 | 826240 | - |  |  |
|  |  |  |  | LPB74_03720 | 826236 | 826479 | - |  |  |
|  |  |  |  | LPB74_03725 | 826677 | 827130 | L | K03111 |  |
|  |  |  |  | LPB74_03730 | 827131 | 827665 | S |  |  |
|  |  |  |  | LPB74_03735 | 827712 | 828492 | S |  |  |
|  |  |  |  | LPB74_03740 | 828484 | 829084 | - |  |  |
|  |  |  |  | LPB74_03745 | 829080 | 830451 | - |  |  |
|  |  |  |  | LPB74_03750 | 830447 | 831185 | S |  |  |
|  |  |  |  | LPB74_03755 | 831221 | 832970 | K |  |  |
|  |  |  |  |  | 832972 | 833236 | L |  |  |
|  |  |  |  | LPB74_03765 | 833225 | 833471 | - |  |  |
|  |  |  |  | LPB74_03770 | 833467 | 833875 | - |  |  |
|  |  |  |  | LPB74_03775 | 833876 | 834218 | - |  |  |
|  |  |  |  | LPB74_03780 | 834214 | 835561 | L | K02314 |  |
|  |  |  |  | LPB74_03785 | 835560 | 836262 | S |  |  |
|  |  |  |  | LPB74_03790 | 836258 | 837014 | - |  |  |
|  |  |  |  | LPB74_03795 | 837015 | 837876 | D | K12055 |  |
|  |  |  |  |  | 838879 | 839086 | K |  |  |
|  |  |  |  |  | 839184 | 839805 | K |  |  |
|  |  |  |  | LPB74_03820 | 839928 | 840087 | - |  |  |
|  |  |  |  | LPB74_03825 | 840332 | 841154 | K | K20330 |  |
|  |  |  |  | LPB74_03830 | 841299 | 841788 | S |  |  |
|  |  |  |  | LPB74_03835 | 842142 | 842769 | E |  |  |
|  |  |  |  | LPB74_03840 | 843291 | 844473 | S |  |  |
|  |  |  |  | LPB74_03845 | 844692 | 845988 | G | K00692 | GH68 protein |
|  |  |  |  |  | 845999 | 846185 | S |  |  |
|  |  |  |  |  | 846694 | 847159 | S |  |  |
| RGP_12 | 864854 | 881617 | 16763 |  | 864854 | 867875 | M |  |  |
|  |  |  |  |  | 868179 | 869490 | I | K06076 |  |
|  |  |  |  | LPB74_03960 | 869774 | 870275 | - |  |  |
|  |  |  |  | LPB74_03965 | 870331 | 870838 | - |  |  |
|  |  |  |  |  | 871063 | 872713 | T |  |  |
|  |  |  |  | LPB74_03975 | 873078 | 873303 | - |  |  |
|  |  |  |  | LPB74_03980 | 873660 | 874917 | M | K13408 |  |
|  |  |  |  | LPB74_03985 | 874913 | 877064 | V | K13409 |  |
|  |  |  |  | LPB74_03990 | 877063 | 878425 | MU | K12340 | outer membrane protein |
|  |  |  |  | LPB74_03995 | 878440 | 879064 | K |  |  |
|  |  |  |  | LPB74_04000 | 879313 | 880927 | T |  |  |
|  |  |  |  | LPB74_04005 | 880949 | 881144 | - |  |  |
|  |  |  |  | LPB74_04010 | 881145 | 881337 | - |  |  |
|  |  |  |  | LPB74_04015 | 881401 | 881617 | - |  |  |
| RGP_9 | 1039133 | 1065334 | 26201 | LPB74_04740 | 1039133 | 1040033 | M |  | GT2 protein |
|  |  |  |  | LPB74_04745 | 1040960 | 1043117 | S |  |  |
|  |  |  |  |  | 1043537 | 1043690 | - |  |  |
|  |  |  |  | LPB74_04750 | 1043852 | 1045259 | M |  |  |
|  |  |  |  | LPB74_04755 | 1045884 | 1047939 | M |  | GT2 protein |
|  |  |  |  | LPB74_04760 | 1048035 | 1048440 | S |  |  |
|  |  |  |  |  | 1048447 | 1049398 | S |  |  |
|  |  |  |  |  | 1049384 | 1050329 | M | K20534 | GT2 protein |
|  |  |  |  | LPB74_04780 | 1050331 | 1051435 | E |  |  |
|  |  |  |  |  | 1051449 | 1052904 | S |  |  |
|  |  |  |  | LPB74_04790 | 1053389 | 1058105 | M |  | GT2 protein |
|  |  |  |  | LPB74_04795 | 1058097 | 1059462 | GM | K09691 |  |
|  |  |  |  | LPB74_04800 | 1059451 | 1060279 | GM | K09690 |  |
|  |  |  |  | LPB74_04805 | 1060278 | 1060824 | M | K01790 |  |
|  |  |  |  |  | 1060820 | 1060976 | - |  |  |
|  |  |  |  | LPB74_04810 | 1061515 | 1062427 | S | K12991 |  |
|  |  |  |  | LPB74_04815 | 1062468 | 1063359 | H | K00973 |  |
|  |  |  |  |  | 1063355 | 1064264 | M | K00067 |  |
|  |  |  |  | LPB74_04825 | 1064260 | 1065334 | M | K01710 |  |
| RGP_26 | 1313573 | 1320045 | 6472 | LPB74_06020 | 1313573 | 1313861 | S |  |  |
|  |  |  |  | LPB74_06025 | 1313902 | 1314856 | I |  | CE10 protein |
|  |  |  |  | LPB74_06030 | 1314904 | 1315924 | G | K00615 |  |
|  |  |  |  | LPB74_06035 | 1315920 | 1316769 | G | K00615 |  |
|  |  |  |  | LPB74_06040 | 1316787 | 1318089 | G |  |  |
|  |  |  |  | LPB74_06045 | 1318189 | 1318939 | IQ |  |  |
|  |  |  |  | LPB74_06050 | 1319127 | 1320045 | K |  |  |
| RGP_11 | 1398293 | 1412608 | 14315 |  | 1398293 | 1398521 | L |  |  |
|  |  |  |  | LPB74_06440 | 1398998 | 1400051 | - |  |  |
|  |  |  |  |  | 1400069 | 1400711 | - |  |  |
|  |  |  |  | LPB74_06445 | 1400854 | 1403194 | L | K03546 |  |
|  |  |  |  | LPB74_06450 | 1403168 | 1403834 | - |  |  |
|  |  |  |  | LPB74_06455 | 1403830 | 1405060 | - |  |  |
|  |  |  |  |  | 1405084 | 1405333 | - |  |  |
|  |  |  |  | LPB74_06460 | 1405340 | 1405535 | - |  |  |
|  |  |  |  | LPB74_06465 | 1405531 | 1406569 | - |  |  |
|  |  |  |  |  | 1406730 | 1406829 | - |  |  |
|  |  |  |  | LPB74_06470 | 1407024 | 1407240 | - |  |  |
|  |  |  |  | LPB74_06475 | 1407280 | 1408270 | E | K01273 |  |
|  |  |  |  |  | 1408441 | 1409389 | V |  |  |
|  |  |  |  | LPB74_06485 | 1409686 | 1410172 | - |  |  |
|  |  |  |  | LPB74_06490 | 1410631 | 1412608 | S |  |  |
| RGP_1 | 1583065 | 1667698 | 84633 | LPB74_07280 | 1583065 | 1584469 | L |  |  |
|  |  |  |  | LPB74_07285 | 1584465 | 1586364 | S |  |  |
|  |  |  |  | LPB74_07290 | 1586792 | 1587485 | E |  |  |
|  |  |  |  | LPB74_07295 | 1587598 | 1588033 | KT | K03503 |  |
|  |  |  |  | LPB74_07300 | 1588019 | 1589300 | L | K03502 |  |
|  |  |  |  | LPB74_07310 | 1589955 | 1591494 | S |  |  |
|  |  |  |  | LPB74_07315 | 1591490 | 1591865 | - |  |  |
|  |  |  |  |  | 1591867 | 1593235 | S |  |  |
|  |  |  |  | LPB74_07325 | 1593255 | 1594194 | S |  |  |
|  |  |  |  | LPB74_07330 | 1594190 | 1594664 | S |  |  |
|  |  |  |  | LPB74_07345 | 1596217 | 1596850 | - |  |  |
|  |  |  |  | LPB74_07350 | 1597094 | 1597805 | O |  |  |
|  |  |  |  | LPB74_07355 | 1597801 | 1598101 | - |  |  |
|  |  |  |  | LPB74_07360 | 1598097 | 1601106 | U |  |  |
|  |  |  |  | LPB74_07365 | 1601105 | 1601561 | S |  |  |
|  |  |  |  | LPB74_07370 | 1601538 | 1603023 | M |  |  |
|  |  |  |  | LPB74_07375 | 1603012 | 1603936 | NU |  |  |
|  |  |  |  | LPB74_07380 | 1603932 | 1604601 | S |  |  |
|  |  |  |  | LPB74_07385 | 1604597 | 1604990 | S |  |  |
|  |  |  |  | LPB74_07390 | 1605005 | 1605374 | S |  |  |
|  |  |  |  | LPB74_07395 | 1605395 | 1605635 | S |  |  |
|  |  |  |  | LPB74_07400 | 1605631 | 1605994 | S |  |  |
|  |  |  |  | LPB74_07405 | 1606244 | 1607192 | S |  |  |
|  |  |  |  | LPB74_07410 | 1607249 | 1607564 | - |  |  |
|  |  |  |  | LPB74_07415 | 1607731 | 1608163 | - |  |  |
|  |  |  |  | LPB74_07420 | 1608186 | 1609644 | L |  |  |
|  |  |  |  | LPB74_07425 | 1609653 | 1610403 | S |  |  |
|  |  |  |  | LPB74_07430 | 1610441 | 1612601 | U |  |  |
|  |  |  |  | LPB74_07435 | 1612612 | 1613119 | KT |  |  |
|  |  |  |  | LPB74_07440 | 1613115 | 1613673 | M |  |  |
|  |  |  |  | LPB74_07445 | 1613657 | 1614404 | O |  |  |
|  |  |  |  | LPB74_07450 | 1614412 | 1615123 | - |  |  |
|  |  |  |  | LPB74_07455 | 1615357 | 1615807 | - |  |  |
|  |  |  |  | LPB74_07460 | 1616145 | 1618410 | L |  |  |
|  |  |  |  | LPB74_07465 | 1618516 | 1619968 | L |  |  |
|  |  |  |  | LPB74_07470 | 1619997 | 1620597 | - |  |  |
|  |  |  |  | LPB74_07475 | 1620722 | 1621334 | S |  |  |
|  |  |  |  | LPB74_07480 | 1621431 | 1621767 | - |  |  |
|  |  |  |  |  | 1621855 | 1622173 | - |  |  |
|  |  |  |  |  | 1622243 | 1622399 | - |  |  |
|  |  |  |  | LPB74_07485 | 1622468 | 1623035 | S |  |  |
|  |  |  |  | LPB74_07490 | 1623131 | 1623362 | - |  |  |
|  |  |  |  | LPB74_07495 | 1623665 | 1624034 | - |  |  |
|  |  |  |  |  | 1624217 | 1624889 | - |  |  |
|  |  |  |  | LPB74_07505 | 1625099 | 1625366 | - |  |  |
|  |  |  |  | LPB74_07510 | 1626186 | 1628208 | L | K03169 |  |
|  |  |  |  |  | 1629248 | 1629875 | K |  |  |
|  |  |  |  | LPB74_07520 | 1629908 | 1631309 | Q | K00808 |  |
|  |  |  |  | LPB74_07525 | 1631310 | 1632396 | - |  |  |
|  |  |  |  | LPB74_07530 | 1632414 | 1633335 | EG |  |  |
|  |  |  |  |  | 1633417 | 1633576 | - |  |  |
|  |  |  |  | LPB74_07535 | 1633607 | 1634291 | - |  |  |
|  |  |  |  |  | 1634292 | 1635243 | L |  |  |
|  |  |  |  | LPB74_07545 | 1635983 | 1636424 | S |  |  |
|  |  |  |  | LPB74_07550 | 1636454 | 1637825 | NU |  |  |
|  |  |  |  | LPB74_07555 | 1637821 | 1638754 | NU |  |  |
|  |  |  |  | LPB74_07560 | 1638756 | 1639281 | S |  |  |
|  |  |  |  | LPB74_07565 | 1639317 | 1640409 | NU |  |  |
|  |  |  |  | LPB74_07570 | 1640398 | 1642030 | NU |  |  |
|  |  |  |  | LPB74_07575 | 1642026 | 1642635 | - |  |  |
|  |  |  |  | LPB74_07580 | 1642624 | 1643983 | S |  |  |
|  |  |  |  | LPB74_07585 | 1643984 | 1645721 | NU |  |  |
|  |  |  |  | LPB74_07590 | 1645717 | 1646794 | S |  |  |
|  |  |  |  |  | 1647097 | 1647865 | - | K13439 | AvrRpt2 (T3E) |
|  |  |  |  | LPB74_07600 | 1647976 | 1649929 | KL |  |  |
|  |  |  |  | LPB74_07605 | 1649931 | 1650273 | - |  |  |
|  |  |  |  | LPB74_07620 | 1651525 | 1651768 | - |  |  |
|  |  |  |  | LPB74_07625 | 1651966 | 1652422 | L | K03111 |  |
|  |  |  |  | LPB74_07630 | 1652423 | 1653008 | S |  |  |
|  |  |  |  | LPB74_07635 | 1653004 | 1653784 | S |  |  |
|  |  |  |  | LPB74_07640 | 1653848 | 1654484 | - |  |  |
|  |  |  |  |  | 1654480 | 1655842 | - |  |  |
|  |  |  |  | LPB74_07650 | 1655847 | 1656585 | S |  |  |
|  |  |  |  | LPB74_07655 | 1656621 | 1658367 | K |  |  |
|  |  |  |  |  | 1658369 | 1658633 | L |  |  |
|  |  |  |  | LPB74_07665 | 1658622 | 1658868 | - |  |  |
|  |  |  |  | LPB74_07670 | 1658864 | 1659272 | - |  |  |
|  |  |  |  | LPB74_07675 | 1659273 | 1659615 | - |  |  |
|  |  |  |  | LPB74_07680 | 1659611 | 1660958 | L | K02314 |  |
|  |  |  |  | LPB74_07685 | 1660957 | 1661659 | S |  |  |
|  |  |  |  | LPB74_07690 | 1661655 | 1662411 | - |  |  |
|  |  |  |  | LPB74_07695 | 1662412 | 1663273 | D | K12055 |  |
|  |  |  |  | LPB74_07700 | 1664267 | 1666181 | NT | K03406 |  |
|  |  |  |  | LPB74_07705 | 1666249 | 1666933 | - |  |  |
|  |  |  |  | LPB74_07710 | 1667026 | 1667698 | K | K10914 |  |
| RGP_36 | 1681030 | 1685460 | 4430 | LPB74_07790 | 1681030 | 1681624 | - |  |  |
|  |  |  |  | LPB74_07795 | 1682219 | 1682519 | D | K19092 |  |
|  |  |  |  | LPB74_07800 | 1682496 | 1682745 | K |  |  |
|  |  |  |  | LPB74_07805 | 1683076 | 1684198 | C | K10680 |  |
|  |  |  |  | LPB74_07810 | 1684530 | 1685460 | K |  |  |
| RGP_35 | 1830179 | 1850281 | 20102 |  | 1830179 | 1830893 | K |  | sylA |
|  |  |  |  | LPB74_08510 | 1831044 | 1832097 | I |  | sylB |
|  |  |  |  |  | 1832318 | 1836287 | Q |  | sylC |
|  |  |  |  |  | 1836406 | 1848973 | Q |  | sylD |
|  |  |  |  |  | 1849045 | 1850281 | EGP | K08151 | sylE |
| RGP_42 | 2074746 | 2083582 | 8836 | LPB74_09440 | 2074746 | 2075886 | S |  |  |
|  |  |  |  |  | 2075882 | 2076416 | - |  |  |
|  |  |  |  | LPB74_09450 | 2076840 | 2078226 | L |  |  |
|  |  |  |  | LPB74_09455 | 2078500 | 2083582 | L |  |  |
| RGP_19 | 2119041 | 2128010 | 8969 |  | 2119041 | 2119383 | - |  |  |
|  |  |  |  | LPB74_09620 | 2119414 | 2120230 | G |  |  |
|  |  |  |  | LPB74_09625 | 2120373 | 2121483 | I |  |  |
|  |  |  |  | LPB74_09630 | 2121498 | 2122428 | H |  |  |
|  |  |  |  | LPB74_09635 | 2122471 | 2123695 | EGP |  |  |
|  |  |  |  | LPB74_09640 | 2123669 | 2124329 | - |  |  |
|  |  |  |  | LPB74_09645 | 2124358 | 2125639 | H | K00836 |  |
|  |  |  |  | LPB74_09650 | 2125638 | 2126349 | H | K06137 |  |
|  |  |  |  | LPB74_09655 | 2126356 | 2126803 | Q |  |  |
|  |  |  |  | LPB74_09660 | 2127086 | 2128010 | K |  |  |
| RGP_34 | 2556869 | 2562817 | 5948 | LPB74_11365 | 2556869 | 2558297 | - |  |  |
|  |  |  |  |  | 2558350 | 2559376 | S |  |  |
|  |  |  |  | LPB74_11375 | 2559315 | 2560056 | S |  |  |
|  |  |  |  | LPB74_11380 | 2560052 | 2561369 | C |  |  |
|  |  |  |  | LPB74_11385 | 2561365 | 2562817 | H |  |  |
| RGP_25 | 2596829 | 2606864 | 10035 | LPB74_11565 | 2596829 | 2599370 | V |  |  |
|  |  |  |  | LPB74_11570 | 2599379 | 2601251 | - |  |  |
|  |  |  |  | LPB74_11575 | 2602026 | 2603868 | S | K11904 | type VI secreted protein |
|  |  |  |  | LPB74_11580 | 2603903 | 2604491 | KLT |  |  |
|  |  |  |  | LPB74_11585 | 2604487 | 2605720 | M |  |  |
|  |  |  |  | LPB74_11590 | 2605716 | 2606298 | S |  |  |
|  |  |  |  | LPB74_11595 | 2606405 | 2606864 | K |  |  |
| RGP_41 | 2634073 | 2640029 | 5956 | LPB74_11725 | 2634073 | 2634316 | - |  |  |
|  |  |  |  |  | 2634432 | 2635293 | S |  |  |
|  |  |  |  | LPB74_11735 | 2635467 | 2635899 | L |  |  |
|  |  |  |  | LPB74_11740 | 2635898 | 2636636 | S |  |  |
|  |  |  |  | LPB74_11745 | 2636658 | 2637036 | T |  |  |
|  |  |  |  |  | 2637032 | 2640029 | T | K11354 |  |
| RGP_17 | 2881088 | 2891762 | 10674 | LPB74_12675 | 2881088 | 2881772 | K |  |  |
|  |  |  |  | LPB74_12680 | 2881933 | 2882359 | S |  |  |
|  |  |  |  | LPB74_12685 | 2882387 | 2883269 | S |  |  |
|  |  |  |  | LPB74_12690 | 2883279 | 2884908 | G | K00854 |  |
|  |  |  |  | LPB74_12695 | 2884904 | 2885588 | G | K01628 |  |
|  |  |  |  | LPB74_12700 | 2885584 | 2887066 | H | K01845 |  |
|  |  |  |  | LPB74_12705 | 2887076 | 2887697 | G |  |  |
|  |  |  |  | LPB74_12710 | 2887719 | 2888895 | EGP |  |  |
|  |  |  |  | LPB74_12715 | 2888942 | 2890196 | EGP |  |  |
|  |  |  |  | LPB74_12720 | 2890218 | 2890494 | - |  |  |
|  |  |  |  | LPB74_12725 | 2890541 | 2891762 | M |  |  |
| RGP_33 | 2922835 | 2929744 | 6909 | LPB74_12840 | 2922835 | 2923576 | K |  |  |
|  |  |  |  | LPB74_12845 | 2923660 | 2924995 | G | K13021 |  |
|  |  |  |  | LPB74_12850 | 2925073 | 2926051 | EH | K00058 |  |
|  |  |  |  | LPB74_12855 | 2926289 | 2926967 | H |  |  |
|  |  |  |  | LPB74_12860 | 2927202 | 2928225 | C |  |  |
|  |  |  |  | LPB74_12865 | 2928654 | 2929509 | K |  | salA |
|  |  |  |  |  | 2929615 | 2929744 | - |  |  |
| RGP_5 | 3072874 | 3109642 | 36768 | LPB74_13000 | 3072874 | 3073558 | K |  |  |
|  |  |  |  | LPB74_13005 | 3073847 | 3074933 | S | K18201 |  |
|  |  |  |  | LPB74_13010 | 3074959 | 3076315 | E |  |  |
|  |  |  |  | LPB74_13015 | 3076506 | 3077175 | S | K01560 |  |
|  |  |  |  | LPB74_13020 | 3077215 | 3078541 | E |  |  |
|  |  |  |  | LPB74_13025 | 3078567 | 3079638 | E | K00641 |  |
|  |  |  |  | LPB74_13030 | 3080140 | 3080860 | - |  | HopBA1 (T3E) |
|  |  |  |  | LPB74_13035 | 3081020 | 3082001 | L | K07481 |  |
|  |  |  |  |  | 3082130 | 3082295 | - |  |  |
|  |  |  |  | LPB74_13040 | 3082515 | 3082980 | O |  |  |
|  |  |  |  | LPB74_13045 | 3083115 | 3084498 | EG | K03299 |  |
|  |  |  |  |  | 3084578 | 3084974 | K | K03710 |  |
|  |  |  |  | LPB74_13055 | 3085352 | 3085817 | O |  |  |
|  |  |  |  |  | 3086474 | 3087917 | - |  |  |
|  |  |  |  | LPB74_13065 | 3087926 | 3088733 | - |  |  |
|  |  |  |  | LPB74_13070 | 3088734 | 3090315 | - |  |  |
|  |  |  |  | LPB74_13075 | 3090326 | 3091388 | - |  |  |
|  |  |  |  |  | 3091562 | 3091730 | L |  |  |
|  |  |  |  | LPB74_13085 | 3092065 | 3092938 | K |  |  |
|  |  |  |  | LPB74_13090 | 3093116 | 3096842 | D |  |  |
|  |  |  |  | LPB74_13095 | 3096838 | 3097501 | - |  |  |
|  |  |  |  | LPB74_13100 | 3097497 | 3098739 | C |  |  |
|  |  |  |  | LPB74_13105 | 3098735 | 3099605 | L |  |  |
|  |  |  |  | LPB74_13110 | 3099696 | 3100155 | S |  |  |
|  |  |  |  |  | 3100350 | 3101781 | S |  |  |
|  |  |  |  |  | 3102018 | 3102408 | E |  |  |
|  |  |  |  | LPB74_13130 | 3103448 | 3104645 | - |  |  |
|  |  |  |  | LPB74_13135 | 3105018 | 3106104 | S |  |  |
|  |  |  |  | LPB74_13140 | 3106222 | 3106717 | - |  |  |
|  |  |  |  |  | 3107415 | 3107562 | - |  |  |
|  |  |  |  |  | 3107601 | 3108051 | - |  |  |
|  |  |  |  | LPB74_13155 | 3108805 | 3109642 | S |  |  |
| RGP_40 | 3138756 | 3143711 | 4955 | LPB74_13280 | 3138756 | 3139899 | G |  | GH1 protein |
|  |  |  |  | LPB74_13285 | 3139901 | 3141083 | M | K01854 |  |
|  |  |  |  | LPB74_13290 | 3141070 | 3142255 | M |  |  |
|  |  |  |  | LPB74_13295 | 3142682 | 3143711 | M | K01784 |  |
| RGP_4 | 3206251 | 3233716 | 27465 |  | 3206251 | 3206848 | - |  |  |
|  |  |  |  | LPB74_13590 | 3207122 | 3207929 | C |  |  |
|  |  |  |  | LPB74_13595 | 3208110 | 3208524 | - |  |  |
|  |  |  |  |  | 3208520 | 3209111 | - |  |  |
|  |  |  |  |  | 3209157 | 3210018 | K |  |  |
|  |  |  |  |  | 3210160 | 3210394 | - |  |  |
|  |  |  |  |  | 3210593 | 3211217 | L |  |  |
|  |  |  |  | LPB74_13625 | 3211332 | 3211611 | - |  |  |
|  |  |  |  | LPB74_13630 | 3211661 | 3211847 | - |  |  |
|  |  |  |  | LPB74_13635 | 3211836 | 3212163 | S | K19092 |  |
|  |  |  |  | LPB74_13640 | 3212363 | 3218102 | NU |  | HopR1 (T3E) |
|  |  |  |  | LPB74_13645 | 3218648 | 3218933 | S |  |  |
|  |  |  |  | LPB74_13650 | 3219178 | 3219739 | L |  |  |
|  |  |  |  |  | 3219722 | 3221381 | L |  |  |
|  |  |  |  | LPB74_13660 | 3221370 | 3222342 | U |  |  |
|  |  |  |  |  | 3222582 | 3224319 | S |  | HopW1 (T3E) |
|  |  |  |  |  | 3224490 | 3224907 | S |  |  |
|  |  |  |  |  | 3225463 | 3225619 | - |  |  |
|  |  |  |  | LPB74_13680 | 3226144 | 3226408 | S |  |  |
|  |  |  |  |  | 3226944 | 3227097 | L |  |  |
|  |  |  |  |  | 3227164 | 3227713 | - |  |  |
|  |  |  |  | LPB74_13695 | 3227791 | 3229432 | NT | K03406 |  |
|  |  |  |  | LPB74_13710 | 3230783 | 3231767 | S |  |  |
|  |  |  |  | LPB74_13715 | 3231794 | 3232826 | E | K00549 |  |
|  |  |  |  |  | 3232822 | 3233104 | - |  |  |
|  |  |  |  |  | 3232984 | 3233716 | S |  | AA10 protein |
| RGP_8 | 3259068 | 3280784 | 21716 | LPB74_13850 | 3259068 | 3259503 | J |  |  |
|  |  |  |  | LPB74_13855 | 3259572 | 3261156 | E | K00274 |  |
|  |  |  |  | LPB74_13860 | 3261178 | 3262108 | ET | K02030 |  |
|  |  |  |  | LPB74_13865 | 3262136 | 3262922 | E | K02028 |  |
|  |  |  |  | LPB74_13870 | 3262914 | 3263904 | P | K02029 |  |
|  |  |  |  | LPB74_13875 | 3263946 | 3266394 | P | K02014 |  |
|  |  |  |  | LPB74_13880 | 3267098 | 3269093 | K | K21405 |  |
|  |  |  |  | LPB74_13885 | 3269287 | 3270310 | S | K00010 |  |
|  |  |  |  | LPB74_13890 | 3270306 | 3271095 | G | K01816 |  |
|  |  |  |  |  | 3271187 | 3271574 | G | K17213 |  |
|  |  |  |  |  | 3271596 | 3272115 | G | K17213 |  |
|  |  |  |  | LPB74_13900 | 3272504 | 3273173 | C | K13483 |  |
|  |  |  |  | LPB74_13905 | 3273169 | 3274120 | C | K11178 |  |
|  |  |  |  | LPB74_13910 | 3274170 | 3276381 | C | K11177 |  |
|  |  |  |  | LPB74_13915 | 3276415 | 3277330 | K |  |  |
|  |  |  |  | LPB74_13920 | 3277326 | 3278031 | S | K01607 |  |
|  |  |  |  | LPB74_13925 | 3278417 | 3279323 | C |  |  |
|  |  |  |  | LPB74_13930 | 3279455 | 3280166 | K |  |  |
|  |  |  |  | LPB74_13935 | 3280298 | 3280784 | - |  |  |
| RGP_29 | 3305169 | 3312868 | 7699 | LPB74_14040 | 3305169 | 3305469 | - |  |  |
|  |  |  |  |  | 3305450 | 3306953 | C |  |  |
|  |  |  |  | LPB74_14050 | 3306952 | 3308188 | S |  |  |
|  |  |  |  | LPB74_14055 | 3308184 | 3308793 | M |  |  |
|  |  |  |  |  | 3309207 | 3310404 | - |  |  |
|  |  |  |  | LPB74_14070 | 3310891 | 3312868 | L |  |  |
| RGP_6 | 3467326 | 3513455 | 3513455 |  | 3467326 | 3467593 | - |  |  |
|  |  |  |  |  | 3468105 | 3468399 | S |  |  |
|  |  |  |  |  | 3469048 | 3469159 | S |  | Clade 2b-a OGs |
|  |  |  |  | LPB74_14815 | 3469594 | 3470179 | - |  |  |
|  |  |  |  |  | 3470175 | 3470367 | GM |  |  |
|  |  |  |  | LPB74_14820 | 3470740 | 3471208 | - |  |  |
|  |  |  |  |  | 3471197 | 3472478 | U |  |  |
|  |  |  |  |  | 3472913 | 3473870 | U | K15125 |  |
|  |  |  |  |  | 3474599 | 3476855 | U | K15125 |  |
|  |  |  |  |  | 3476827 | 3477583 | U |  |  |
|  |  |  |  | LPB74_14840 | 3477770 | 3478301 | S |  |  |
|  |  |  |  | LPB74_14855 | 3479617 | 3479893 | S |  |  |
|  |  |  |  | LPB74_14860 | 3480375 | 3480987 | - |  |  |
|  |  |  |  | LPB74_14865 | 3481470 | 3482001 | - |  |  |
|  |  |  |  | LPB74_14870 | 3482003 | 3485798 | U | K15125 |  |
|  |  |  |  | LPB74_14885 | 3486791 | 3487004 | U |  |  |
|  |  |  |  | LPB74_14890 | 3486978 | 3487428 | U |  |  |
|  |  |  |  | LPB74_14895 | 3488198 | 3488711 | - |  |  |
|  |  |  |  |  | 3488710 | 3493822 | U | K15125 |  |
|  |  |  |  |  | 3495389 | 3495575 | L | K07483 |  |
|  |  |  |  | LPB74_14920 | 3495650 | 3496292 | - |  |  |
|  |  |  |  | LPB74_14925 | 3496301 | 3513455 | U | K15125 |  |
| RGP_18 | 4055323 | 4072428 | 17105 | LPB74_17330 | 4055323 | 4056073 | IQ | K00059 |  |
|  |  |  |  |  | 4056278 | 4057154 | K |  |  |
|  |  |  |  | LPB74_17340 | 4057170 | 4058145 | S |  |  |
|  |  |  |  |  | 4058141 | 4058237 | - |  |  |
|  |  |  |  | LPB74_17345 | 4058247 | 4058991 | IQ |  |  |
|  |  |  |  | LPB74_17350 | 4059129 | 4060023 | K |  |  |
|  |  |  |  | LPB74_17355 | 4060060 | 4060624 | K | K16137 |  |
|  |  |  |  |  | 4060724 | 4060826 | - |  |  |
|  |  |  |  | LPB74_17360 | 4060822 | 4061524 | H |  |  |
|  |  |  |  | LPB74_17365 | 4061863 | 4064275 | M | K05802 |  |
|  |  |  |  |  | 4064398 | 4064956 | - |  | Clade 2b-a OGs |
|  |  |  |  | LPB74_17375 | 4065287 | 4065527 | - |  | Clade 2b-a OGs |
|  |  |  |  |  | 4065767 | 4066343 | S |  | Clade 2b-a OGs |
|  |  |  |  | LPB74_17390 | 4066513 | 4067524 | D |  | Clade 2b-a OGs |
|  |  |  |  | LPB74_17395 | 4067739 | 4068849 | V |  |  |
|  |  |  |  | LPB74_17400 | 4069040 | 4070876 | L |  |  |
|  |  |  |  | LPB74_17405 | 4070866 | 4071370 | - |  |  |
|  |  |  |  | LPB74_17410 | 4071357 | 4072428 | - |  |  |
| RGP_21 | 4257524 | 4286393 | 28869 | LPB74_18320 | 4257524 | 4277543 | Q |  |  |
|  |  |  |  | LPB74_18325 | 4278205 | 4278802 | C |  |  |
|  |  |  |  | LPB74_18330 | 4278835 | 4279432 | K |  |  |
|  |  |  |  | LPB74_18335 | 4279579 | 4280791 | EGP | K07552 |  |
|  |  |  |  | LPB74_18340 | 4281063 | 4281300 | - |  |  |
|  |  |  |  | LPB74_18345 | 4281560 | 4282244 | K | K07666 |  |
|  |  |  |  | LPB74_18350 | 4282240 | 4283689 | T | K07645 |  |
|  |  |  |  | LPB74_18355 | 4283571 | 4284843 | V | K19147 |  |
|  |  |  |  |  | 4284977 | 4286393 | V | K07452 |  |
| RGP_2 | 4312948 | 4356341 | 43393 | LPB74_18450 | 4312948 | 4313353 | S |  |  |
|  |  |  |  | LPB74_18460 | 4314074 | 4314818 | - |  |  |
|  |  |  |  | LPB74_18465 | 4314839 | 4316084 | - |  |  |
|  |  |  |  |  | 4316080 | 4316299 | - |  |  |
|  |  |  |  | LPB74_18475 | 4316316 | 4316847 | S |  |  |
|  |  |  |  | LPB74_18480 | 4316843 | 4317389 | S | K03791 | GH19 protein |
|  |  |  |  | LPB74_18485 | 4317453 | 4318863 | - |  |  |
|  |  |  |  | LPB74_18490 | 4318886 | 4319573 | - |  |  |
|  |  |  |  |  | 4319572 | 4319893 | - |  |  |
|  |  |  |  | LPB74_18500 | 4319889 | 4323642 | S |  |  |
|  |  |  |  | LPB74_18505 | 4323697 | 4324285 | S |  |  |
|  |  |  |  | LPB74_18510 | 4324281 | 4325064 | M |  |  |
|  |  |  |  | LPB74_18515 | 4325066 | 4325765 | S |  |  |
|  |  |  |  | LPB74_18520 | 4325894 | 4326962 | - |  |  |
|  |  |  |  | LPB74_18525 | 4327141 | 4327486 | S |  |  |
|  |  |  |  | LPB74_18530 | 4327485 | 4330059 | D |  |  |
|  |  |  |  | LPB74_18535 | 4330106 | 4330253 | - |  |  |
|  |  |  |  | LPB74_18540 | 4330309 | 4330747 | S |  |  |
|  |  |  |  | LPB74_18545 | 4330756 | 4331254 | S |  |  |
|  |  |  |  | LPB74_18550 | 4331318 | 4331687 | S |  |  |
|  |  |  |  | LPB74_18555 | 4331686 | 4332172 | S |  |  |
|  |  |  |  | LPB74_18560 | 4332164 | 4332503 | S |  |  |
|  |  |  |  | LPB74_18565 | 4332499 | 4332832 | S |  |  |
|  |  |  |  |  | 4332828 | 4333167 | - |  |  |
|  |  |  |  | LPB74_18575 | 4333169 | 4334339 | S |  |  |
|  |  |  |  | LPB74_18580 | 4334342 | 4335005 | S | K06904 |  |
|  |  |  |  | LPB74_18585 | 4335022 | 4336294 | S |  |  |
|  |  |  |  | LPB74_18590 | 4336293 | 4338015 | K |  |  |
|  |  |  |  | LPB74_18595 | 4338015 | 4338501 | L |  |  |
|  |  |  |  | LPB74_18600 | 4338668 | 4339064 | V | K07451 |  |
|  |  |  |  | LPB74_18605 | 4339054 | 4339255 | - |  | HopBK1 (T3E) |
|  |  |  |  |  | 4339315 | 4339528 | - |  |  |
|  |  |  |  | LPB74_18610 | 4339560 | 4339920 | - |  |  |
|  |  |  |  | LPB74_18615 | 4339919 | 4340291 | - |  |  |
|  |  |  |  | LPB74_18620 | 4340598 | 4340982 | S |  |  |
|  |  |  |  | LPB74_18625 | 4340984 | 4341305 | S |  |  |
|  |  |  |  | LPB74_18630 | 4341310 | 4342588 | L |  |  |
|  |  |  |  | LPB74_18635 | 4342584 | 4342896 | - |  |  |
|  |  |  |  | LPB74_18640 | 4342895 | 4343228 | - |  |  |
|  |  |  |  | LPB74_18645 | 4343224 | 4343656 | S |  |  |
|  |  |  |  | LPB74_18650 | 4343652 | 4343949 | S |  |  |
|  |  |  |  |  | 4343945 | 4344434 | S |  |  |
|  |  |  |  | LPB74_18660 | 4344613 | 4345387 | K |  |  |
|  |  |  |  |  | 4345609 | 4345801 | - |  |  |
|  |  |  |  |  | 4346291 | 4346552 | K |  |  |
|  |  |  |  | LPB74_18675 | 4346562 | 4346898 | - |  |  |
|  |  |  |  | LPB74_18680 | 4347220 | 4347622 | T |  |  |
|  |  |  |  |  | 4347950 | 4348460 | - |  |  |
|  |  |  |  | LPB74_18685 | 4348597 | 4348936 | - |  |  |
|  |  |  |  | LPB74_18690 | 4348935 | 4349526 | - |  |  |
|  |  |  |  | LPB74_18695 | 4349522 | 4349777 | - |  |  |
|  |  |  |  |  | 4349833 | 4350982 | L |  |  |
|  |  |  |  | LPB74_18705 | 4351036 | 4351819 | S |  |  |
|  |  |  |  | LPB74_18710 | 4351886 | 4352426 | - |  |  |
|  |  |  |  |  | 4352422 | 4352581 | - |  |  |
|  |  |  |  | LPB74_18715 | 4352640 | 4353429 | - |  |  |
|  |  |  |  | LPB74_18720 | 4353425 | 4354097 | - |  |  |
|  |  |  |  | LPB74_18725 | 4354099 | 4354312 | - |  |  |
|  |  |  |  | LPB74_18730 | 4354435 | 4354933 | - |  |  |
|  |  |  |  | LPB74_18735 | 4355156 | 4356341 | L | K14059 |  |
| RGP_16 | 4399961 | 4411365 | 11404 | LPB74_18965 | 4399961 | 4400639 | S |  |  |
|  |  |  |  | LPB74_18970 | 4400628 | 4402110 | I |  |  |
|  |  |  |  | LPB74_18975 | 4402106 | 4402796 | K |  |  |
|  |  |  |  | LPB74_18980 | 4402782 | 4403592 | S |  |  |
|  |  |  |  | LPB74_18985 | 4403588 | 4404230 | NU |  |  |
|  |  |  |  | LPB74_18990 | 4404240 | 4404903 | T | K07666 |  |
|  |  |  |  | LPB74_18995 | 4404899 | 4406267 | T | K07645 |  |
|  |  |  |  |  | 4406306 | 4407557 | T | K02484 |  |
|  |  |  |  | LPB74_19005 | 4407553 | 4408243 | K | K02483 |  |
|  |  |  |  | LPB74_19010 | 4408459 | 4410832 | M | K07277 |  |
|  |  |  |  | LPB74_19015 | 4410951 | 4411365 | - |  |  |
| RGP_15 | 4416616 | 4432280 | 15664 | LPB74_19045 | 4416616 | 4417531 | - |  |  |
|  |  |  |  |  | 4417592 | 4417727 | - |  |  |
|  |  |  |  | LPB74_19050 | 4417801 | 4418023 | M |  |  |
|  |  |  |  |  | 4418034 | 4418256 | M |  |  |
|  |  |  |  | LPB74_19060 | 4418629 | 4420384 | - |  | Clade 2b-a OGs |
|  |  |  |  |  | 4420670 | 4422830 | S |  | Clade 2b-a OGs |
|  |  |  |  | LPB74_19070 | 4422989 | 4423757 | - |  | Clade 2b-a OGs |
|  |  |  |  |  | 4424473 | 4424752 | L | K07483 |  |
|  |  |  |  | LPB74_19080 | 4424896 | 4425988 | L |  | Clade 2b-a OGs |
|  |  |  |  | LPB74_19085 | 4425989 | 4427213 | - |  | Clade 2b-a OGs |
|  |  |  |  |  | 4427354 | 4428344 | Q | K18058 | Clade 2b-a OGs |
|  |  |  |  | LPB74_19095 | 4428411 | 4429860 | - |  | Clade 2b-a OGs |
|  |  |  |  | LPB74_19100 | 4430060 | 4430531 | M | K16053 |  |
|  |  |  |  | LPB74_19105 | 4430938 | 4431793 | - |  | HopAF1 (T3E) |
|  |  |  |  |  | 4432028 | 4432280 | L | K07483 |  |
| RGP_32 | 4437691 | 4444867 | 7176 | LPB74_19130 | 4437691 | 4437943 | - |  |  |
|  |  |  |  | LPB74_19135 | 4438520 | 4439642 | S | K03924 | Clade 2b-a OGs |
|  |  |  |  | LPB74_19140 | 4439638 | 4440085 | - |  | Clade 2b-a OGs |
|  |  |  |  |  | 4440084 | 4441920 | - |  | Clade 2b-a OGs |
|  |  |  |  | LPB74_19150 | 4441906 | 4444867 | - |  | Clade 2b-a OGs |
| RGP_24 | 4749054 | 4757132 | 8078 | LPB74_20460 | 4749054 | 4751103 | D |  |  |
|  |  |  |  | LPB74_20465 | 4751092 | 4752781 | S |  |  |
|  |  |  |  | LPB74_20470 | 4752777 | 4753047 | S |  |  |
|  |  |  |  | LPB74_20475 | 4753095 | 4754652 | S |  |  |
|  |  |  |  | LPB74_20485 | 4755206 | 4756187 | L | K07481 |  |
|  |  |  |  |  | 4756218 | 4756707 | - |  |  |
|  |  |  |  | LPB74_20495 | 4756802 | 4757132 | - |  |  |
| RGP_20 | 4883533 | 4889776 | 6243 | LPB74_21105 | 4883533 | 4884037 | S | K11903 | type VI secretion system protein |
|  |  |  |  |  | 4884481 | 4884907 | S |  |  |
|  |  |  |  | LPB74_21120 | 4885085 | 4885304 | - |  |  |
|  |  |  |  |  | 4885775 | 4886045 | S |  | Clade 2b-a OGs |
|  |  |  |  |  | 4886007 | 4887111 | L | K14059 |  |
|  |  |  |  | LPB74_21135 | 4887189 | 4887600 | L |  | Clade 2b-a OGs |
|  |  |  |  | LPB74_21140 | 4887565 | 4888879 | L | K07459 | Clade 2b-a OGs |
|  |  |  |  |  | 4888910 | 4889012 | - |  | Clade 2b-a OGs |
|  |  |  |  | LPB74_21145 | 4889311 | 4889776 | K |  |  |
| RGP_14 | 5008477 | 5046609 | 38132 | LPB74_21650 | 5008477 | 5008675 | - |  |  |
|  |  |  |  | LPB74_21655 | 5009235 | 5010114 | H |  |  |
|  |  |  |  | LPB74_21665 | 5010751 | 5012308 | S | K07028 |  |
|  |  |  |  | LPB74_21670 | 5012408 | 5013008 | E |  |  |
|  |  |  |  | LPB74_21675 | 5013146 | 5014064 | I | K00648 |  |
|  |  |  |  | LPB74_21680 | 5014132 | 5017300 | I | K15329 |  |
|  |  |  |  | LPB74_21685 | 5017304 | 5024087 | Q |  |  |
|  |  |  |  | LPB74_21690 | 5024142 | 5031324 | Q |  |  |
|  |  |  |  | LPB74_21695 | 5031334 | 5041030 | Q | K13612 |  |
|  |  |  |  |  | 5041050 | 5043027 | E | K01953 |  |
|  |  |  |  | LPB74_21705 | 5043262 | 5043508 | Q |  |  |
|  |  |  |  | LPB74_21710 | 5043582 | 5044596 | Q | K03119 |  |
|  |  |  |  | LPB74_21720 | 5045622 | 5046609 | K | K03566 |  |
| RGP_39 | 5157106 | 5163487 | 6381 |  | 5157106 | 5157448 | L |  |  |
|  |  |  |  | LPB74_22220 | 5157707 | 5158169 | S | K09796 |  |
|  |  |  |  | LPB74_22225 | 5158562 | 5161643 | - |  |  |
|  |  |  |  |  | 5161677 | 5162217 | L | K07483 |  |
|  |  |  |  |  | 5162423 | 5163053 | L | K07497 |  |
|  |  |  |  |  | 5163244 | 5163487 | - |  |  |
| RGP_28 | 5224279 | 5230146 | 5867 |  | 5224279 | 5224429 | V |  |  |
|  |  |  |  |  | 5224681 | 5225401 | U | K03832 |  |
|  |  |  |  | LPB74_22565 | 5225484 | 5226213 | U | K03561 |  |
|  |  |  |  | LPB74_22570 | 5226212 | 5226638 | U | K03559 |  |
|  |  |  |  | LPB74_22575 | 5226752 | 5228873 | P | K02014 |  |
|  |  |  |  | LPB74_22580 | 5228880 | 5230146 | S | K01093 |  |
| RGP_27 | 5300331 | 5305230 | 4899 | LPB74_23000 | 5300331 | 5300877 | S | K03791 | GH19 protein |
|  |  |  |  | LPB74_23005 | 5301107 | 5301605 | - |  |  |
|  |  |  |  |  | 5301592 | 5301769 | - |  |  |
|  |  |  |  | LPB74_23010 | 5301881 | 5302940 | - |  |  |
|  |  |  |  | LPB74_23015 | 5303195 | 5303762 | S |  |  |
|  |  |  |  | LPB74_23020 | 5303769 | 5305230 | G |  |  |
| RGP_7 | 5359949 | 5386166 | 26217 | LPB74_23290 | 5359949 | 5361065 | L |  |  |
|  |  |  |  | LPB74_23295 | 5360979 | 5361954 | - |  |  |
|  |  |  |  |  | 5362042 | 5362279 | - |  |  |
|  |  |  |  | LPB74_23300 | 5362278 | 5363157 | L |  |  |
|  |  |  |  | LPB74_23305 | 5363143 | 5363896 | - |  |  |
|  |  |  |  | LPB74_23310 | 5364214 | 5364787 | - |  |  |
|  |  |  |  | LPB74_23315 | 5365006 | 5366599 | S |  |  |
|  |  |  |  | LPB74_23320 | 5366884 | 5367205 | - |  |  |
|  |  |  |  |  | 5367207 | 5368701 | U | K07344 |  |
|  |  |  |  | LPB74_23330 | 5368948 | 5369680 | U | K20266 |  |
|  |  |  |  |  | 5369676 | 5369910 | - |  |  |
|  |  |  |  |  | 5370470 | 5370716 | - |  |  |
|  |  |  |  | LPB74_23335 | 5370805 | 5371414 | - |  |  |
|  |  |  |  | LPB74_23340 | 5371402 | 5372470 | S |  |  |
|  |  |  |  | LPB74_23345 | 5372456 | 5373023 | E |  |  |
|  |  |  |  | LPB74_23350 | 5373031 | 5373625 | - |  |  |
|  |  |  |  | LPB74_23355 | 5374220 | 5374727 | O | K07304 |  |
|  |  |  |  | LPB74_23360 | 5375167 | 5375983 | P | K21993 |  |
|  |  |  |  | LPB74_23365 | 5376159 | 5376735 | E |  |  |
|  |  |  |  | LPB74_23370 | 5376784 | 5377621 | K |  |  |
|  |  |  |  | LPB74_23375 | 5377919 | 5378513 | - |  |  |
|  |  |  |  | LPB74_23380 | 5378567 | 5378891 | S |  |  |
|  |  |  |  | LPB74_23385 | 5378883 | 5379375 | - |  |  |
|  |  |  |  | LPB74_23390 | 5379469 | 5380102 | H | K06133 |  |
|  |  |  |  | LPB74_23395 | 5380098 | 5384139 | Q |  |  |
|  |  |  |  | LPB74_23400 | 5384391 | 5384895 | K |  |  |
|  |  |  |  | LPB74_23405 | 5385035 | 5386166 | S |  |  |
| RGP_31 | 5549137 | 5557496 | 8359 | LPB74_24105 | 5549137 | 5552029 | S | K11904 | type VI secreted protein |
|  |  |  |  |  | 5552080 | 5554048 | I |  |  |
|  |  |  |  |  | 5554134 | 5555103 | S | K07126 |  |
|  |  |  |  | LPB74_24120 | 5555237 | 5556293 | S | K07126 |  |
|  |  |  |  | LPB74_24125 | 5556440 | 5557496 | S | K07126 |  |
| RGP_23 | 5678390 | 5684966 | 6576 | LPB74_24675 | 5678390 | 5678582 | - |  |  |
|  |  |  |  |  | 5678646 | 5678775 | - |  |  |
|  |  |  |  | LPB74_24680 | 5679670 | 5680996 | H |  |  |
|  |  |  |  |  | 5681046 | 5682195 | GM |  |  |
|  |  |  |  | LPB74_24690 | 5682198 | 5682879 | - |  |  |
|  |  |  |  | LPB74_24695 | 5683434 | 5683707 | - |  |  |
|  |  |  |  | LPB74_24700 | 5683985 | 5684966 | L | K07481 |  |
| RGP_3 | 5717582 | 5758588 | 41006 | LPB74_24835 | 5717582 | 5718065 | S | K11897 |  |
|  |  |  |  |  | 5718061 | 5719897 | S | K11896 |  |
|  |  |  |  | LPB74_24845 | 5719860 | 5720931 | S | K11895 |  |
|  |  |  |  | LPB74_24850 | 5720927 | 5723531 | O | K11907 | type VI secretion system protein |
|  |  |  |  | LPB74_24855 | 5723610 | 5724321 | S | K11906 | type VI secretion system protein |
|  |  |  |  | LPB74_24860 | 5724317 | 5725661 | S | K11893 |  |
|  |  |  |  | LPB74_24865 | 5725657 | 5726371 | S | K11892 | type VI secretion system protein |
|  |  |  |  | LPB74_24870 | 5726398 | 5730292 | N | K11891 | type VI secretion system protein |
|  |  |  |  | LPB74_24875 | 5730326 | 5731247 | S | K11890 |  |
|  |  |  |  | LPB74_24880 | 5731246 | 5733742 | N | K11891 | type VI secretion system protein |
|  |  |  |  | LPB74_24885 | 5733888 | 5734407 | S | K11903 | type VI secretion system protein |
|  |  |  |  | LPB74_24890 | 5734511 | 5735600 | S | K11902 |  |
|  |  |  |  | LPB74_24895 | 5735633 | 5735888 | S |  |  |
|  |  |  |  | LPB74_24900 | 5735898 | 5737056 | S |  |  |
|  |  |  |  | LPB74_24905 | 5737052 | 5738045 | - |  |  |
|  |  |  |  | LPB74_24910 | 5738028 | 5738895 | - |  |  |
|  |  |  |  | LPB74_24915 | 5738942 | 5741843 | S | K11904 | type VI secreted protein |
|  |  |  |  | LPB74_24920 | 5741846 | 5743814 | I |  |  |
|  |  |  |  |  | 5743817 | 5744852 | S | K07126 |  |
|  |  |  |  | LPB74_24935 | 5745498 | 5748255 | S | K11904 | type VI secreted protein |
|  |  |  |  |  | 5748251 | 5748692 | - |  | Clade 2b-a OGs |
|  |  |  |  | LPB74_24945 | 5749236 | 5750091 | - |  |  |
|  |  |  |  | LPB74_24950 | 5750381 | 5750810 | S |  |  |
|  |  |  |  |  | 5750954 | 5751962 | I | K00648 |  |
|  |  |  |  | LPB74_24960 | 5751958 | 5752975 | M |  |  |
|  |  |  |  | LPB74_24965 | 5752967 | 5753774 | S |  |  |
|  |  |  |  | LPB74_24970 | 5753770 | 5755054 | H |  |  |
|  |  |  |  | LPB74_24975 | 5755050 | 5755671 | I |  |  |
|  |  |  |  | LPB74_24980 | 5755667 | 5756801 | I |  |  |
|  |  |  |  | LPB74_24985 | 5756793 | 5757888 | I | K00508 |  |
|  |  |  |  |  | 5757922 | 5758588 | - |  |  |
| RGP_38 | 5875397 | 5884438 | 9041 | LPB74_25500 | 5875397 | 5876279 | P | K07217 |  |
|  |  |  |  | LPB74_25505 | 5876315 | 5876513 | - |  |  |
|  |  |  |  | LPB74_25510 | 5876618 | 5877116 | S |  |  |
|  |  |  |  | LPB74_25515 | 5877167 | 5877371 | S |  |  |
|  |  |  |  | LPB74_25520 | 5877510 | 5878962 | S |  |  |
|  |  |  |  | LPB74_25525 | 5878958 | 5879903 | J |  |  |
|  |  |  |  | LPB74_25530 | 5879995 | 5881849 | S | K11904 | type VI secreted protein |
|  |  |  |  |  | 5882042 | 5883056 | S |  |  |
|  |  |  |  | LPB74_25540 | 5883052 | 5883607 | - |  |  |
|  |  |  |  |  | 5884093 | 5884438 | P |  |  |
| RGP_10 | 5893599 | 5912617 | 19018 |  | 5893599 | 5893746 | - |  |  |
|  |  |  |  | LPB74_25600 | 5893872 | 5894304 | - |  |  |
|  |  |  |  |  | 5894346 | 5896587 | C | K04034 |  |
|  |  |  |  | LPB74_25610 | 5896647 | 5897697 | E |  |  |
|  |  |  |  | LPB74_25615 | 5897705 | 5898992 | Q |  |  |
|  |  |  |  |  | 5898995 | 5900660 | Q |  |  |
|  |  |  |  | LPB74_25625 | 5900809 | 5901763 | E | K21949 |  |
|  |  |  |  |  | 5901719 | 5907134 | Q |  |  |
|  |  |  |  |  | 5907275 | 5907905 | L | K07497 |  |
|  |  |  |  |  | 5908111 | 5908651 | L | K07483 |  |
|  |  |  |  | LPB74_25640 | 5908611 | 5909292 | Q |  |  |
|  |  |  |  | LPB74_25645 | 5909325 | 5910108 | S | K25154 |  |
|  |  |  |  | LPB74_25650 | 5910107 | 5910899 | S | K25155 |  |
|  |  |  |  |  | 5910895 | 5911921 | S | K25156 |  |
|  |  |  |  |  | 5912122 | 5912260 | O |  |  |
|  |  |  |  |  | 5912335 | 5912617 | L |  |  |

**Table S8** *Pseudomonas syringae* strains used in this study

. Isolates from this study are indicated with bold text.

| **Strain** | **Phylogroup** | **Country** | **Common name** | **Host species** | **Host family** | **Accession** |
| --- | --- | --- | --- | --- | --- | --- |
| 03-19A | 2a | USA | cantaloupe | *Cucumis melo* | *Cucurbitaceae* | MUHN00000000 |
| 13-139B | 2a | USA | watermelon | *Citrullus lanatus* | *Cucurbitaceae* | MVAT00000000 |
| 13-429 | 2a | USA | watermelon | *Citrullus lanatus* | *Cucurbitaceae* | MVAY00000000 |
| 200-1 | 2a | Georgia | squash | *Cucurbita pepo* | *Cucurbitaceae* | MVAZ00000000 |
| 31R1 | 2a | NA | corn | *Zea mays* | *Poaceae* | GCA_900105295.1 |
| A2 | 2a | NA | pear | *Pyrus calleryana* | *Rosaceae* | LGKU00000000 |
| BRIP39023 | 2a | Australia | barley | *Hordeum vulgare* | *Poaceae* | AMZX00000000 |
| BS2121 | 2a | USA | squash | *Cucurbita pepo* | *Cucurbitaceae* | MVAV00000000 |
| CFBP1754PT | 2a | Canada | apple | *Malus sylvestris* | *Rosaceae* | JYHI00000000 |
| ICMP11293 | 2a | New Zealand | kiwifruit | *Actinidia deliciosa* | *Actinidiaceae* | LKEP00000000 |
| NFACC10-1 | 2a | NA | switchgrass | *Panicum virgatum* | *Poaceae* | FPJB00000000 |
| P66 | 2a | Thailand | zucchini | *Cucurbita pepo* | *Cucurbitaceae* | WJSF00000000 |
| P73 | 2a | USA | zucchini | *Cucurbita pepo* | *Cucurbitaceae* | WJPS00000000 |
| P77 | 2a | France | zucchini | *Cucurbita pepo* | *Cucurbitaceae* | WJPT00000000 |
| P87 | 2a | China | zucchini | *Cucurbita pepo* | *Cucurbitaceae* | WJPX00000000 |
| P89 | 2a | France | zucchini | *Cucurbita pepo* | *Cucurbitaceae* | WJPY00000000 |
| Pc58T | 2a | Poland | sour cherry | *Prunus cerasus* | *Rosaceae* | PCPL58T |
| UMAF0158 | 2a | Spain | mango | *Mangifera indica* | *Anacardiaceae* | ASM128136v1 |
| ZUM3984 | 2a | China | squash | *Cucurbita pepo* | *Cucurbitaceae* | MVAX00000000 |
| 1845 | 2b | Russia | sunflower | *Helianthus annuus* | *Asteraceae* | LYUP00000000 |
| 2507 | 2b | Russia | wheat | *Triticum aestivum* | *Poaceae* | LYUO00000000 |
| 41A | 2b | France | armenian plum | *Prunus armeniaca* | *Rosaceae* | JYHJ00000000 |
| Alf3 | 2b | USA | alfalfa | *Medicago sativa* | *Fabaceae* | JPNN00000000 |
| B64 | 2b | USA | wheat | *Triticum aestivum* | *Poaceae* | ANZF00000000 |
| BRIP34881 | 2b | Australia | barley | *Hordeum vulgare* | *Poaceae* | AMXL00000000 |
| BS0292 | 2b | NA | sugar beet | *Beta vulgaris* | *Amaranthaceae* | FOVV00000000 |
| BS3827 | 2b | NA | sugar beet | *Beta vulgaris* | *Amaranthaceae* | FOQB00000000 |
| BS3829 | 2b | NA | sugar beet | *Beta vulgaris* | *Amaranthaceae* | FOPR00000000 |
| CRAFRU11 | 2b | Italy | hazelnut | *Corylus avellana* | *Betulaceae* | ATSU00000000 |
| HS191 | 2b | Australia | millet | *Panicum miliaceum* | *Poaceae* | RBNV00000000 |
| ICMP11168 | 2b | New Zealand | kiwifruit | *Actinidia deliciosa* | *Actinidiaceae* | LKGV00000000 |
| ICMP3023T | 2b | UK | liliac | *Syringa vulgaris* | *Oleaceae* | LJRK00000000 |
| ICMP3947PT | 2b | NA | corn | *Zea sp.* | *Poaceae* | LJQQ00000000 |
| ICMP4394PT | 2b | New Zealand | wheat | *Triticum aestivum* | *Poaceae* | LJPO00000000 |
| ICMP459PT | 2b | USA | sugar beet | *Beta vulgaris* | *Amaranthaceae* | LJRP00000000 |
| MB03 | 2b | China | poplar | *Populus lasiocarpa* | *Salicaceae* | LAGV00000000 |
| P108 | 2b | Chile | zucchini | *Cucurbita pepo* | *Cucurbitaceae* | VLIA00000000 |
| Pla1188_1 | 2b | USA | zucchini | *Cucurbita pepo* | *Cucurbitaceae* | RBPG00000000 |
| PP1 | 2b | Japan | pear | *Pisum sativum* | *Fabaceae* | ASM45244v3 |
| PsyCC440 | 2b | France | cantaloupe | *Cucumis melo* | *Cucurbitaceae* | AVEC00000000 |
| SM | 2b | USA | wheat | *Triticum aestivum* | *Poaceae* | APWT01000000 |
| 13-140A | 2ba | USA | watermelon | *Citrullus lanatus* | *Cucurbitaceae* | MUHL00000000 |
| 13-509A | 2ba | Florida | squash | *Cucurbita pepo* | *Cucurbitaceae* | MUHP00000000 |
| 13-C2 | 2ba | Florida | watermelon | *Citrullus lanatus* | *Cucurbitaceae* | MUHO00000000 |
| 14-32 | 2ba | Florida | watermelon | *Citrullus lanatus* | *Cucurbitaceae* | MUHM00000000 |
| 14-410 | 2ba | USA | watermelon | *Citrullus lanatus* | *Cucurbitaceae* | MUHQ00000000 |
| 14-Gil | 2ba | Florida | watermelon | *Citrullus lanatus* | *Cucurbitaceae* | MVAU00000000 |
| **77-4C** | **2ba** | **Australia** | **zucchini** | ***Cucurbita pepo*** | ***Cucurbitaceae*** | **CP087568** |
| **BRIP64883-a** | **2ba** | **Australia** | **zucchini** | ***Cucurbita pepo*** | ***Cucurbitaceae*** | **JAJISZ000000000** |
| **BRIP65014-a** | **2ba** | **Australia** | **zucchini** | ***Cucurbita pepo*** | ***Cucurbitaceae*** | **JAJISY000000000** |
| **BRIP65014-b** | **2ba** | **Australia** | **zucchini** | ***Cucurbita pepo*** | ***Cucurbitaceae*** | **JAJISX000000000** |
| **BRIP65014-c** | **2ba** | **Australia** | **zucchini** | ***Cucurbita pepo*** | ***Cucurbitaceae*** | **JAJISW000000000** |
| **BRIP65014-d** | **2ba** | **Australia** | **zucchini** | ***Cucurbita pepo*** | ***Cucurbitaceae*** | **JAJISV000000000** |
| **BRIP65018-b** | **2ba** | **Australia** | **zucchini** | ***Cucurbita pepo*** | ***Cucurbitaceae*** | **JAJISU000000000** |
| **BRIP65018-d** | **2ba** | **Australia** | **zucchini** | ***Cucurbita pepo*** | ***Cucurbitaceae*** | **JAJIST000000000** |
| **BRIP65019-a** | **2ba** | **Australia** | **zucchini** | ***Cucurbita pepo*** | ***Cucurbitaceae*** | **JAJISS000000000** |
| CC457 | 2ba | France | cantaloupe | *Cucumis melo* | *Cucurbitaceae* | AVEB00000000 |
| **KFR003-1** | **2ba** | **Australia** | **zucchini** | ***Cucurbita pepo*** | ***Cucurbitaceae*** | **JAJITB000000000** |
| **KL004-k1** | **2ba** | **Australia** | **zucchini** | ***Cucurbita pepo*** | ***Cucurbitaceae*** | **JAJITA000000000** |
| P12831 | 2ba | France | zucchini | *Cucurbita pepo* | *Cucurbitaceae* | WJPZ00000000 |
| P12855 | 2ba | Chile | zucchini | *Cucurbita pepo* | *Cucurbitaceae* | VLHZ00000000 |
| P12857 | 2ba | France | zucchini | *Cucurbita pepo* | *Cucurbitaceae* | VLIB00000000 |
| P139 | 2ba | China | zucchini | *Cucurbita pepo* | *Cucurbitaceae* | WLIH00000000 |
| P22 | 2ba | USA | zucchini | *Cucurbita pepo* | *Cucurbitaceae* | WJPQ00000000 |
| P79 | 2ba | France | zucchini | *Cucurbita pepo* | *Cucurbitaceae* | WJPV00000000 |
| P84 | 2ba | China | zucchini | *Cucurbita pepo* | *Cucurbitaceae* | WJPW00000000 |
| P99 | 2ba | France | zucchini | *Cucurbita pepo* | *Cucurbitaceae* | WJSE00000000 |
| PS711 | 2ba | Serbia | squash | *Cucurbita pepo* | *Cucurbitaceae* | RQXZ01000000 |
| ZUM3584 | 2ba | Italy | squash | *Cucurbita pepo* | *Cucurbitaceae* | MVBA01000000 |
| 2339 | 2d | Hungary | sweet cherry | *Prunus avium* | *Rosaceae* | LIHU00000000 |
| 2340 | 2d | Hungary | pear | *Pyrus sp.* | *Rosaceae* | LIHT00000000 |
| ATCC10853PT | 2d | USA | maple | *Acer sp* | *Aceraceae* | LGAR00000000 |
| B301D | 2d | UK | comice pear | *Pyrus communis* | *Rosaceae* | ASM98848v1 |
| B728a | 2d | USA | bean | *Phaseolus vulgaris* | *Fabaceae* | ASM1224v1 |
| HRI-W7872 | 2d | UK | plum | *Prunus domestica* | *Rosaceae* | LIHS00000000 |
| HRI-W7924 | 2d | UK | cherry | *Prunus cerasus* | *Rosaceae* | LIHR00000000 |
| ICMP13102 | 2d | France | kiwifruit | *Actinidia deliciosa* | *Actinidiaceae* | LKEO00000000 |
| PD2774 | 2d | USA | kiwifruit | *Actinidia chinensis* | *Actinidiaceae* | LKEL00000000 |
| PsyCC94 | 2d | France | cantaloupe | *Cucumis melo* | *Cucurbitaceae* | AVEA00000000 |
| USA011 | 2d | USA | fresh water | Not available | Not available | AVDX00000000 |
| CRAFRU12 | Pav | Italy | hazelnut | *Corylus avellana* | *Betulaceae* | ATSV00000000 |
| NCPPB4273T | Pav | Italy | hazelnut | *Corylus avellana* | *Betulaceae* | AWQP00000000 |
| Pav013 | Pav | Italy | hazelnut | *Corylus avellana* | *Betulaceae* | AKCJ01000000 |
| Pav037 | Pav | Italy | hazelnut | *Corylus avellana* | *Betulaceae* | AKCK01000000 |

**Table S9** Genes included in the phytotoxin database manually created for this study

| **Phytotoxin** | **Genbank accession** | **Gene** |
| --- | --- | --- |
| Coronatine  [3] | NP_794429.1 | *cfl* |
|  | NP_794430.1 | *cfa-1* |
|  | NP_794431.1 | *cfa-2* |
|  | NP_794432.1 | *cfa-3* |
|  | NP_794433.1 | *cfa-4* |
|  | NP_794434.1 | *cfa-5* |
|  | NP_794435.1 | *cfa-6* |
|  | NP_794436.1 | *cfa-7* |
|  | NP_794438.1 | *cfa-8* |
|  | NP_794439.1 | *cfa-9* |
|  | NP_794453.1 | *cmaA* |
|  | NP_794454.1 | *cmaB* |
|  | NP_794455.1 | *cmaC* |
|  | NP_794451.1 | *cmaD* |
|  | NP_794452.1 | *cmaE* |
|  | NP_794456.1 | *cmaT* |
|  | NP_794458.1 | *cmaU* |
| Mangotoxin  [4, 5] | AFI41896.1 | *mboA* |
|  | AFI41897.1 | *mboB* |
|  | AFI41898.1 | *mboC* |
|  | AFI41899.1 | *mboD* |
|  | AFI41900.1 | *mboE* |
|  | AFI41901.1 | *mboF* |
|  | ABG00046.1 | *mgoA* |
|  | ABG00044.1 | *mgoB* |
|  | ABG00045.1 | *mgoC* |
|  | ABG00047.1 | *mgoD* |
| Phaseolotoxin  [6] | AAZ99797.1 | *argK* |
|  | AAZ99798.1 | *ptx2* |
|  | AAZ99799.1 | *ptx3* |
|  | AAZ99800.1 | *ptx4* |
|  | AAZ99801.1 | *ptx5* |
|  | AAZ99802.1 | *ptx6* |
|  | AAZ99803.1 | *ptx7* |
|  | AAZ99804.1 | *ptx8* |
|  | AAZ99805.1 | *ptx9* |
|  | AAZ99806.1 | *desA* |
|  | AAZ99818.1 | *ptx11* |
|  | AAZ99819.1 | *ptx12* |
|  | AAZ99807.1 | *ptx13* |
|  | AAZ99808.1 | *ptx14* |
|  | AAZ99809.1 | *ptx15* |
|  | AAZ99810.1 | *ptx16* |
|  | AAZ99811.1 | *amtA* |
|  | AAZ99812.1 | *ptx18* |
|  | AAZ99813.1 | *ptx19* |
|  | AAZ99814.1 | *ptx20* |
|  | AAZ99815.1 | *ptx21* |
|  | AAZ99816.1 | *ptx22* |
| Syringolin  [7, 8] | ELS42861.1 | *salA* |
|  | ELS43791.1 | *sylE* |
|  | ELS43792.1 | *sylD* |
|  | ELS43793.1 | *sylC* |
|  | ELS43794.1 | *sylB* |
|  | ELS43795.1 | *sylA* |
| Syringomycin  [9-11] | AKF46132.1 | *syrB1* |
|  | AKF46131.1 | *syrB2* |
|  | AKF46130.1 | *syrC* |
|  | AKF46129.1 | *syrE* |
| Syringopeptin  [9, 11] | AKF46135.1 | *sypA* |
|  | AKF46136.1 | *sypB* |
|  | AKF46137.1 | *sypC* |
|  | AKF46140.1 | *dat-4* |
| Tabtoxin  [12] | AAL99263.1 | *tabP* |
|  | AAM13981.1 | *tabD* |
|  | P31852.2 | *tabB* |
|  | AAB41802.1 | *tabA* |
|  | AAB25380.1 | *tblA* |
|  | AAM75344.1 | *tabC* |
|  | AAM77668.1 | *tblS* |
|  | AAO63153.1 | *tblC* |
|  | AAU95210.1 | *tblD* |
|  | AAP13070.1 | *tblE* |
|  | AAP13071.1 | *tblF* |
|  | AAP13072.1 | *tblR* |

**Table S10** Genes in the siderophore database manually created for this study

| Siderophore | Locus tag | Gene |
| --- | --- | --- |
| Achromobactin  [13] | Psyr_2580 | *Psyr_2580* |
|  | Psyr_2581 | *Psyr_2581* |
|  | Psyr_2582 | *Psyr_2582* |
|  | Psyr_2583 | *acsF* |
|  | Psyr_2584 | *acsD* |
|  | Psyr_2585 | *acsE* |
|  | Psyr_2586 | *yhcA* |
|  | Psyr_2587 | *acsC* |
|  | Psyr_2588 | *acsB* |
|  | Psyr_2589 | *acsA* |
|  | Psyr_2590 | *cbrA* |
|  | Psyr_2591 | *cbrB* |
|  | Psyr_2592 | *cbrC* |
|  | Psyr_2593 | *cbrD* |
|  | Psyr_2594 | *Psyr_2594* |
|  | Psyr_2595 | *Psyr_2595* |
| Pyoverdine  [14] | PSPPH_1909 | *pvdS* |
|  | PSPPH_1910 | *pvdG* |
|  | PSPPH_1911 | *pvdL* |
|  | PSPPH_1912 | *pvdH* |
|  | PSPPH_1913 | *PSPPH_1913* |
|  | PSPPH_1914 | *PSPPH_1914* |
|  | PSPPH_1915 | *PSPPH_1915* |
|  | PSPPH_1916 | *PSPPH_1916* |
|  | PSPPH_1917 | *PSPPH_1917* |
|  | PSPPH_1918 | *PSPPH_1918* |
|  | PSPPH_1919 | *PSPPH_1919* |
|  | PSPPH_1920 | *PSPPH_1920* |
|  | PSPPH_1921 | *PSPPH_1921* |
|  | PSPPH_1922 | *PSPPH_1922* |
|  | PSPPH_1923 | *pvd-I* |
|  | PSPPH_1924 | *pvd-II* |
|  | PSPPH_1925 | *pvd-III* |
|  | PSPPH_1926 | *pvd-IV* |
|  | PSPPH_1927 | *fpvA_1* |
|  | PSPPH_1928 | *fpvA_2* |
|  | PSPPH_1929 | *pvdE* |
|  | PSPPH_1930 | *pvdO* |
|  | PSPPH_1931 | *pvdN* |
|  | PSPPH_1932 | *pvdM* |
|  | PSPPH_1933 | *pvdP* |
|  | PSPPH_1934 | *PSPPH_1934* |
|  | PSPPH_1935 | *PSPPH_1935* |
|  | PSPPH_1936 | *PSPPH_1936* |
|  | PSPPH_1937 | *pvdQ* |
| Yersiniabactin  [15] | PSPPH_2893 | *PSPPH_2893* |
|  | PSPPH_2894 | *PSPPH_2894* |
|  | PSPPH_2895 | *PSPPH_2895* |
|  | PSPPH_2896 | *PSPPH_2896* |
|  | PSPPH_2897 | *PSPPH_2897* |
|  | PSPPH_2898 | *PSPPH_2898* |
|  | PSPPH_2899 | *irp1* |
|  | PSPPH_2900 | *irp3* |
|  | PSPPH_2901 | *irp4* |
|  | PSPPH_2902 | *irp5* |
|  | PSPPH_2903 | *pchB* |
|  | PSPPH_2904 | *pchA* |

**Table S11** Ice nucleation protein sequences

| **Locus tag** | **Protein sequence** |
| --- | --- |
| PsyrB_07820 (active)  [16] | MNLDKALVLRTCANNMADHCGLIWPASGTVESKYWQSTRRHENGLVGLLWGAGTSAFLSVHADARWIVCEVAVADIISLDEPGMVKFPRAEVVHVGDRISASHFISARQADPASTPPPTSMTTPPPTPAAAHVTLPVAASVTLPVAEQASHEVFDVALVIAAAPSVNTLPVTTPQNLQTATYGSTLSGDNHSRLIAGYGSNETAGNHSDLIAGYGSTGTAGSDSSLVAGYGSTQTAGGDSALTAGYGSTQTAREGSNLTAGYGSTGTAGSDSSLIAGYGSTQTSGEDSSLTAGYGSTQTAQEGSNLTAGYGSTGTAGSDSSLIAGYGSTQTSGGDSSLTAGYGSTQTAQEGSNLTSGYGSTGTAGADSSLIAGYGSTQTSGSDSALTAGYGSTQTAQQGSNLTAGYGSTGTAGSDSSLIAGYGSTQTSGSDSSLTAGYGSTQTAQEGSNLTAGYGSTGTAGVDSSLIAGYGSTQTSGSDSALTAGYGSTQTAQEGSNLTAGYGSTGTAGADSSLIAGYGSTQTSGSDSALTAGYGRRKAAIYGGLRQHWHGWCRSSLIAGYGSTQTSGSDSALTAGYGSTQTAQEGSNLTAGYGSTGTAGADSSLIAGYGSTQTSGSESSLTAGYGSTQTAREGSTLTAGYGSTGTAGADSSLIAGYGSTQTSGSESSLTAGYGSTQTAQQGSVLTSGYGSTQTAGAASNLTTGYGSTGTAGHESFIIAGYGSTQTAGHKSILTAGYGSTQTARDGSDLIAGYGSTGTSGSSSSLIAGYGSTQTASYKSMLTAGYGSTQTAREHSDLVAGYGSTSTAGSNSSLIAGYGSTQTAGFKSILTAGYGSTQTAQERSDLVAGYGSTSTAGYSSSLIAGYGSTQTAGYGSTLTTGYGSTQTAQENSSLTTGYGSTSTAGYSSSLIAGYGSTQTAGYESTLTAGYGSTQTAQERSDLVTGYGSTSTAGYASSLIAGYGSTQTAGYESTLTAGYGSTQTAQENSSLTTGYGSTSTAGFASSLIAGYGSTQTAGYKSTLTAGYGSTQTAEYGSSLTAGYGSTATAGQDSSLIAGYGSSLTSGIRSFLTAGYGSTLIAGLRSVLIAGYGSSLTSGIRSTLTAGYGSNQIASYGSSLIAGHESIQVAGNKSMLIAGKGSSQTAGFRSTLIAGAGSVQLAGDRSRLIAGADSNQTAGDRSKLLAGNNSYLTAGDRSKLTGGHDCTLMAGDQSRLTAGKNSILTAGARSKLIGSEGSTLSAGEDSTLIFRLWDGKRYRQLVAKTGENGVEADIPYYVNEDDDIVDKPDEEDDWIEVE |
| PsyrH_18495 (inactive)  [17] | MNLDKALVLRTCANNMADHCGLIWPASGTVESKYWQSTRRHENGLVGLLWGAGTSAFLSVHADARWIVCEVAVADIISLEEPGMVKFPRAEVVHVGDRISASHFISARQADPASTPTPTPTPMTAPTPTPATANVTLPVAEQASHEVFDVALVSAAAPPINTLPVTTPQNLQTATYGSTLSGDNHSRLIAGYGSNETAGNHSDLIAGYGSTGTAGSDSSLVAGYGSTQTAGGDSALTAGYGSTQTAREGSNLTAGYGSTGTAGSDSSLIAGYGSTQTSGEDSSLTAGYGSTQTAQEGSNLTAGYGSTGTAGSDSSLIAGYGSTQTSGGDSSLTAGYGSTQTAQEGSNLTAGYGSTGTAGSDSSLTAGYGSTQTAQENSSLTTGYGSTSTAGFASSLIAGYGSTQTAGYKSTLTAGYGSTQTAEYGSSLTAGYGSTATAGQDSSLIAGYGSSLTSGIRSFLTAGYGSTLIAGLRSVLIAGYGSSLTSGIRSTLTAGYGSNQIASYGSSLIAGHESIQVAGNKSMLIAGKGSSQTAGFRSTLIAGAGSVQLAGDRSRLIAGADSNQTAGDRSKLLAGNNSYLTAGDRSKLTGGHDCTLMAGDQSRLTAGKNSVLTAGARSKLIGSEGSTLSAGEDSTLIFRLWDGKRYRQLVARTGENGVEADIPYYVNEDDDIVDKPDEDDDWIEVK |


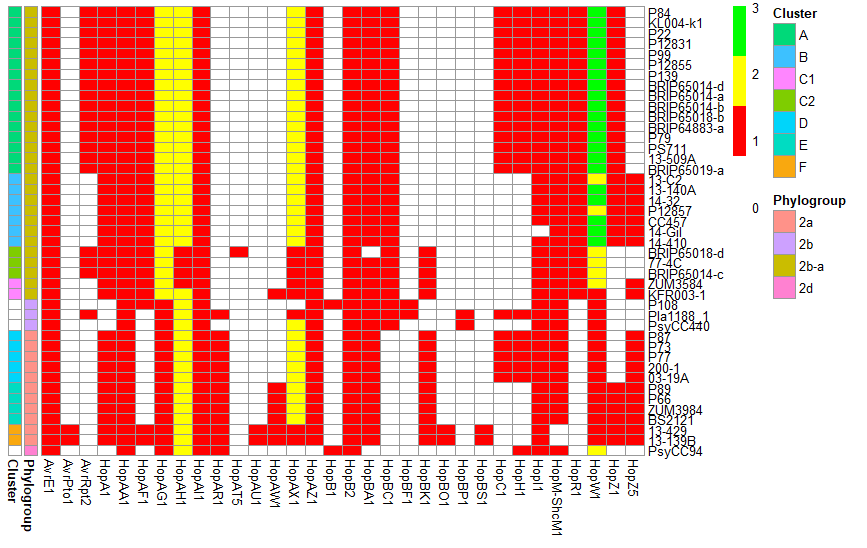


**Figure S1** Type III effector profile of *Pseudomonas syringae* phylogroup 2 isolated from *Cucurbitaceae*

. Colour indicates copies of effector in the genome as shown in the key. Cluster and phylogroup are also indicated by colours described in the key.


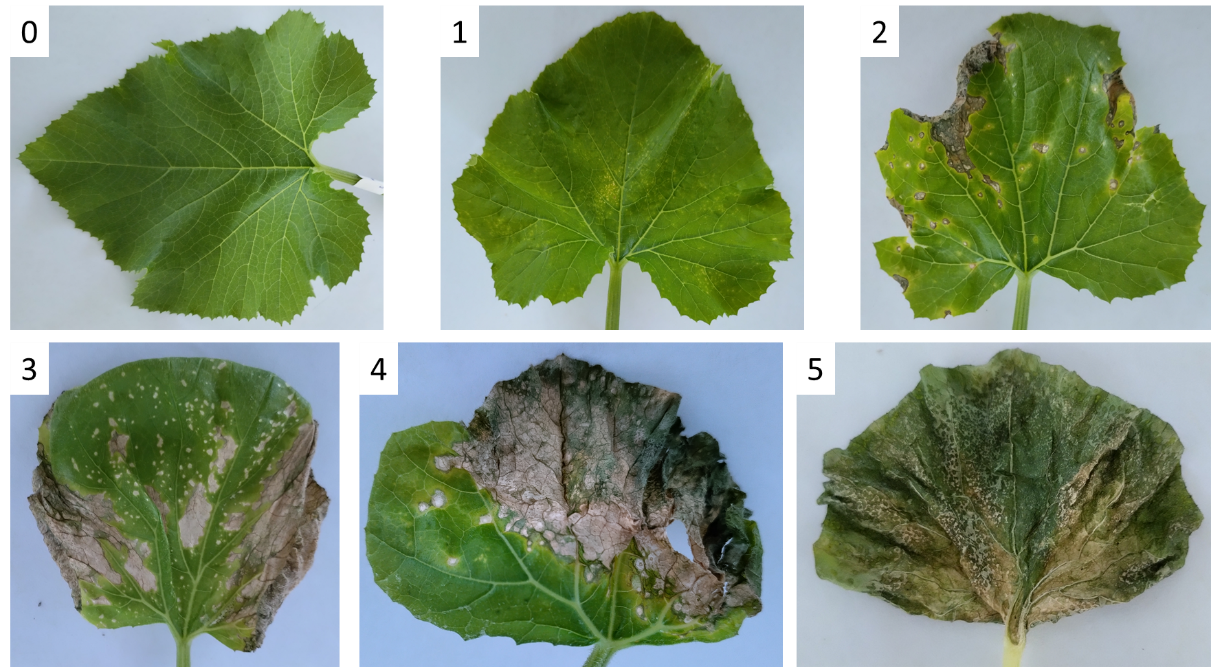


**Figure S2** Disease severity rating scale

. 0 = no symptom, 1 = leaf spot, 2 = necrotic lesions covering <25% of the leaf surface, 3 = necrotic lesions covering from 25 to 49% of the leaf surface, 4 = necrotic lesions covering from 50 to 74% and 5 = necrotic lesions covering ≥75% of the leaf surface.

**Figure S3** Representative of leaf symptoms in *Cucurbitaceae* hosts with spray inoculation of isolates 77-4C, KFR003-1 and KL004-k1

| **Host \ Isolate** | **77-4C** | **KFR003-1** | **KL004-k1** | **Control** |
| --- | --- | --- | --- | --- |
| Zucchini - Eva | 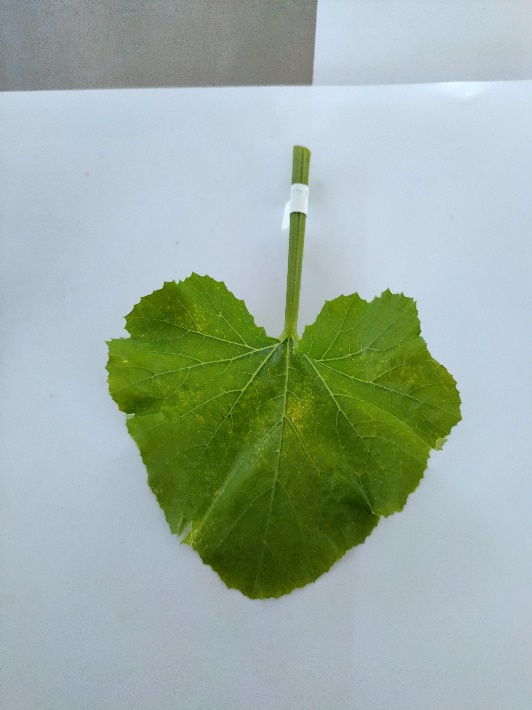 | 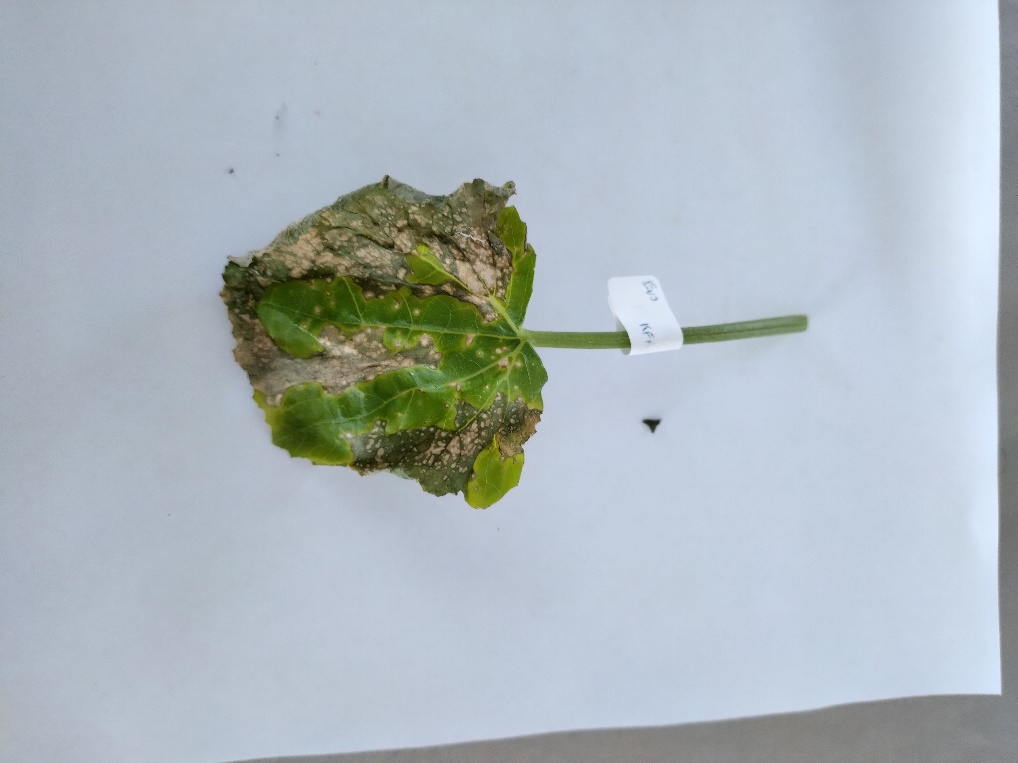 | 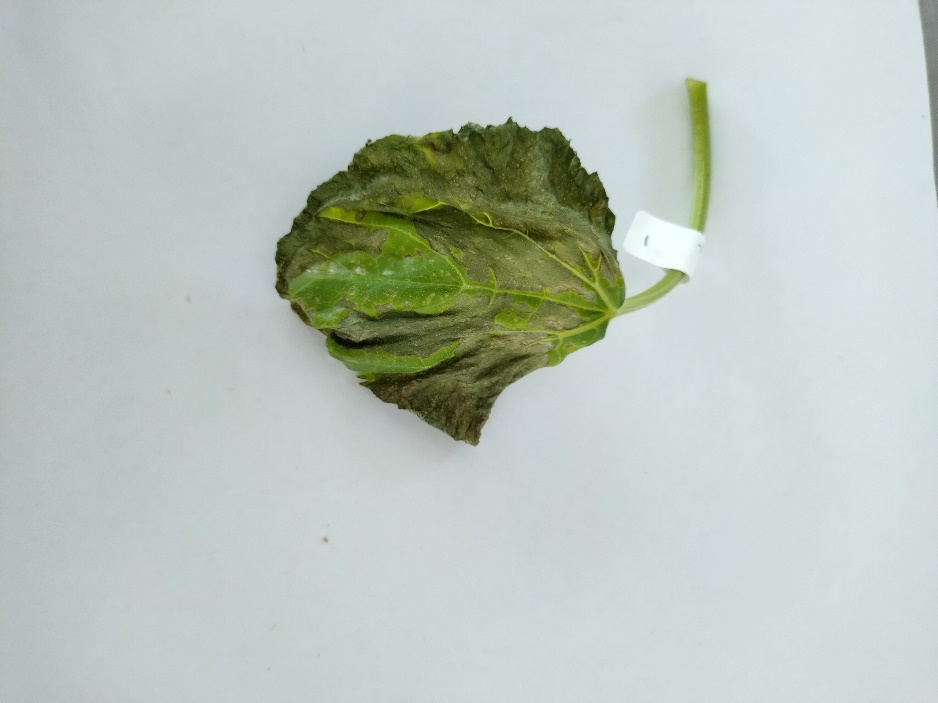 | 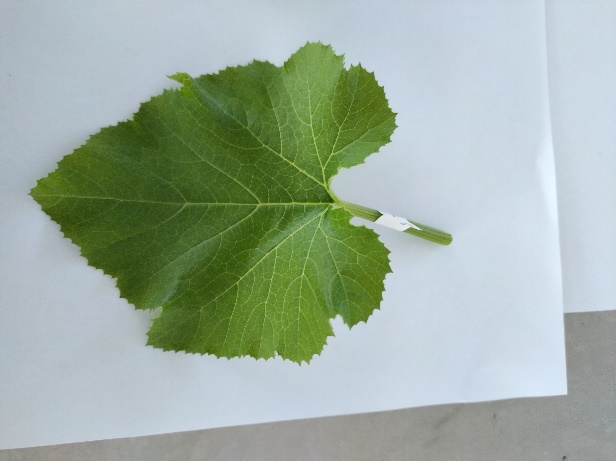 |
| Zucchini - Rosa | 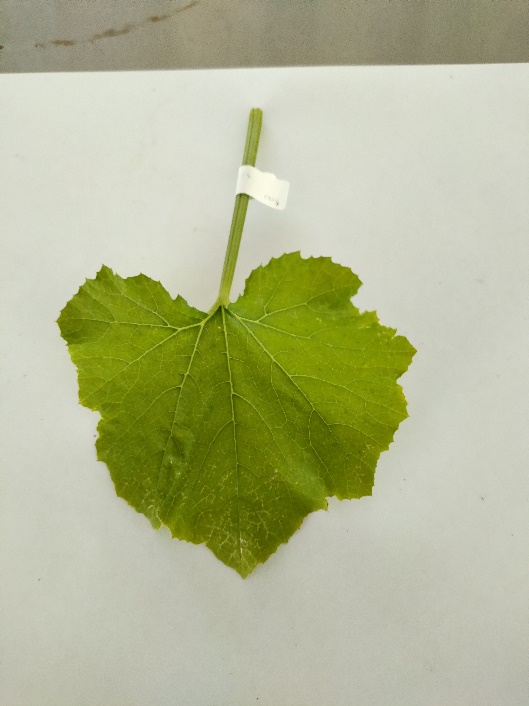 | 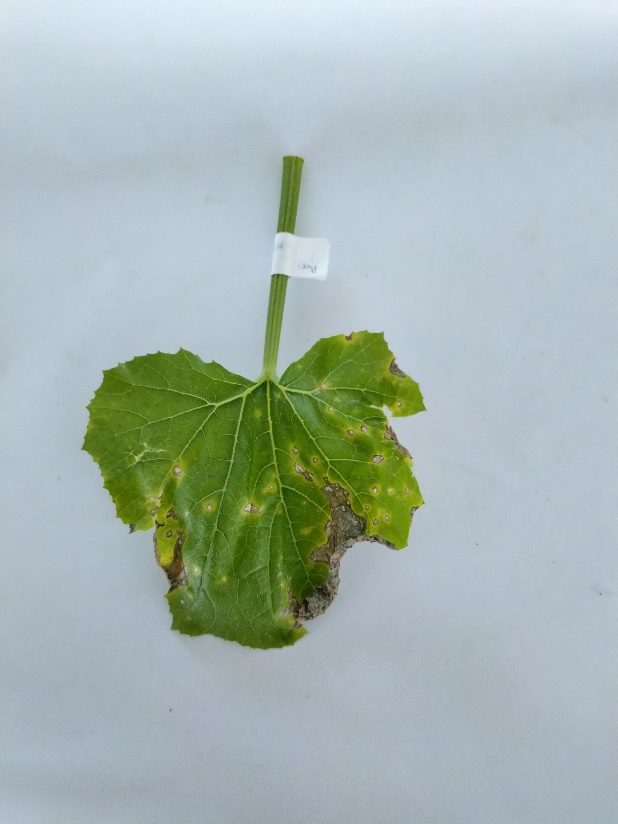 | 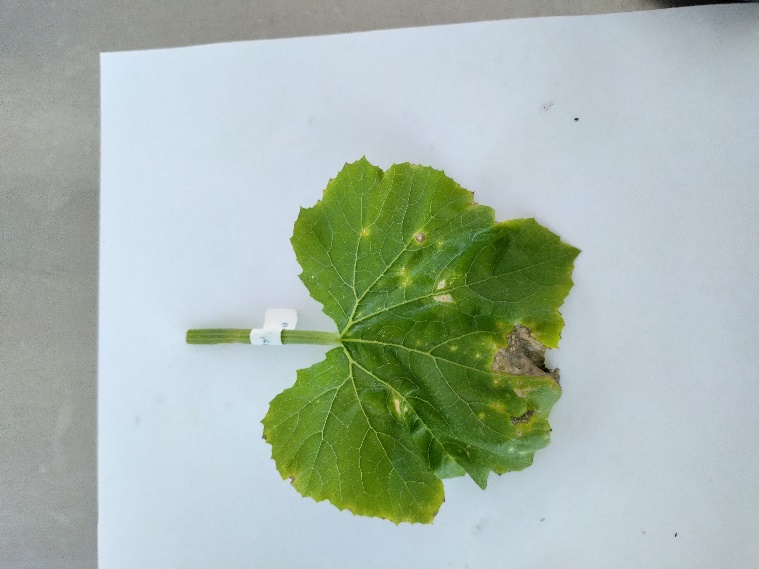 | 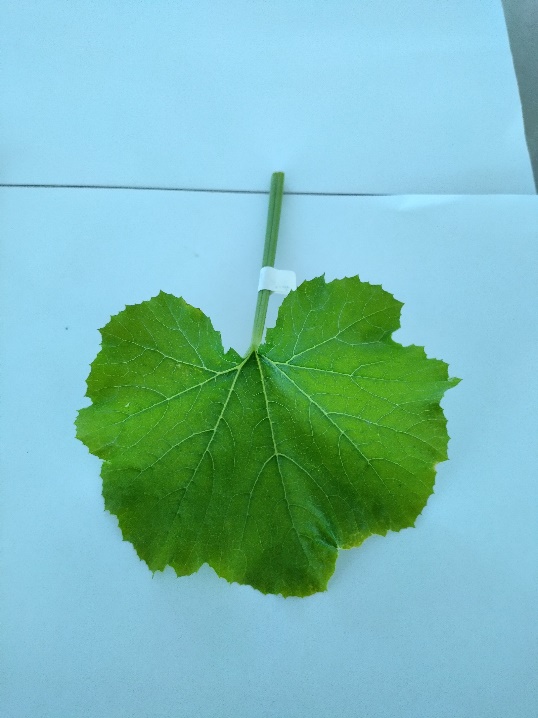 |
| Pumpkin | 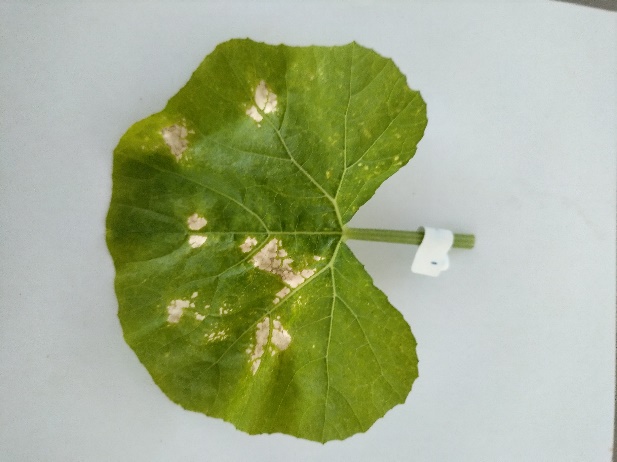 | 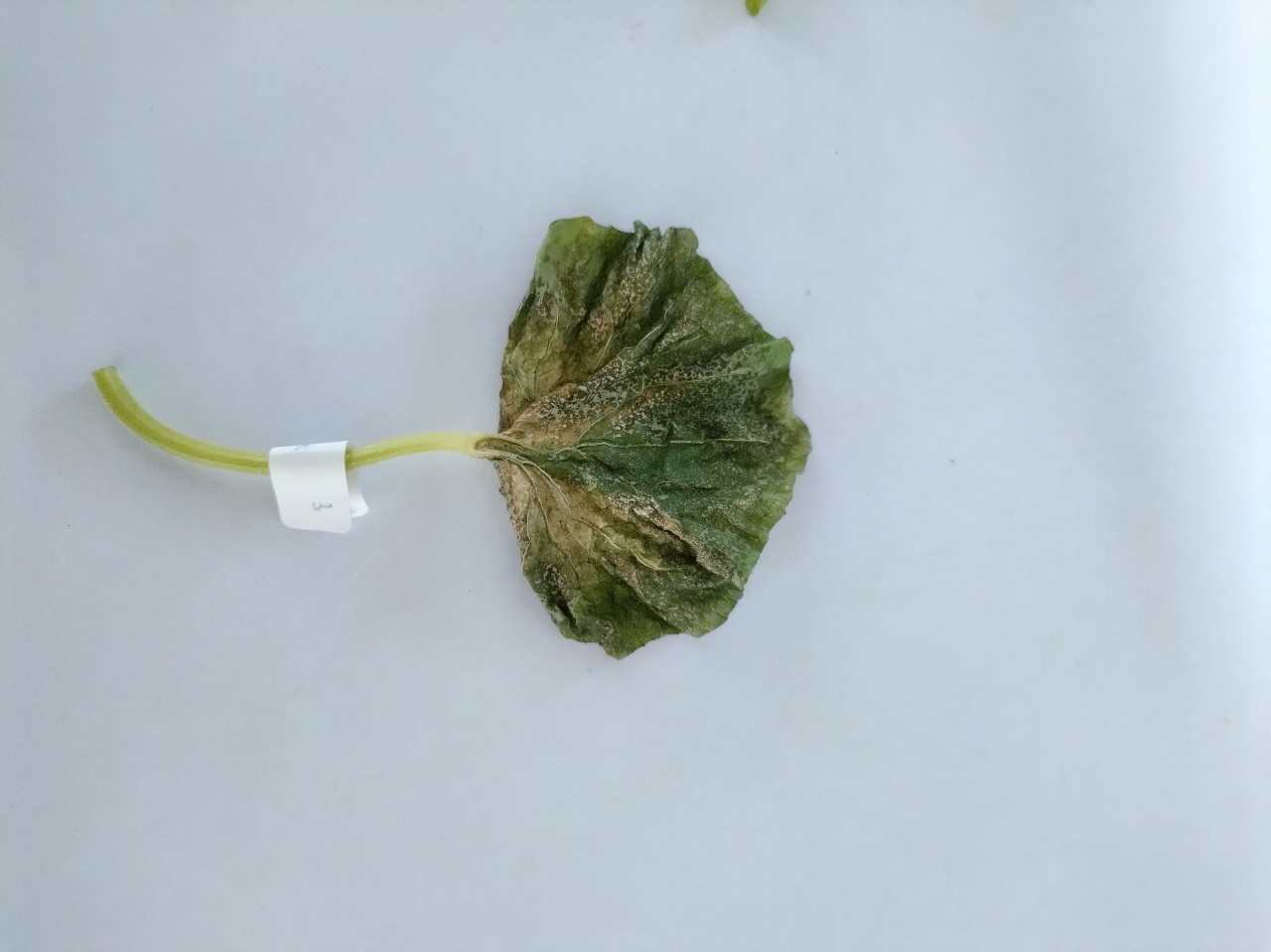 | 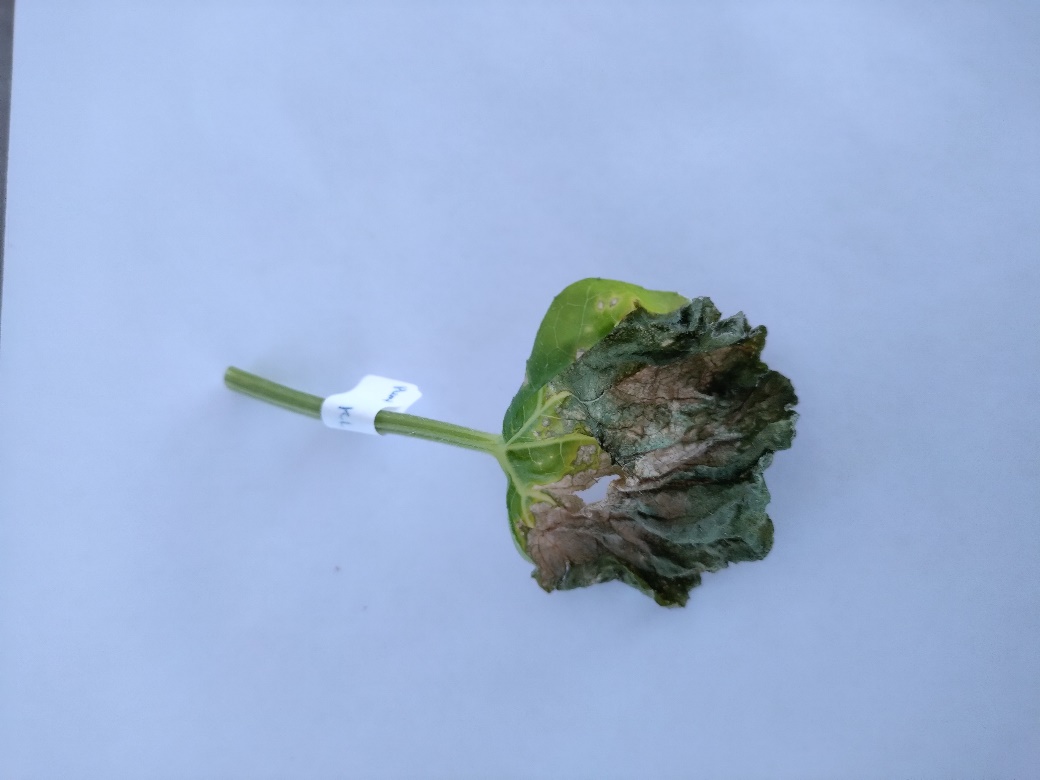 | 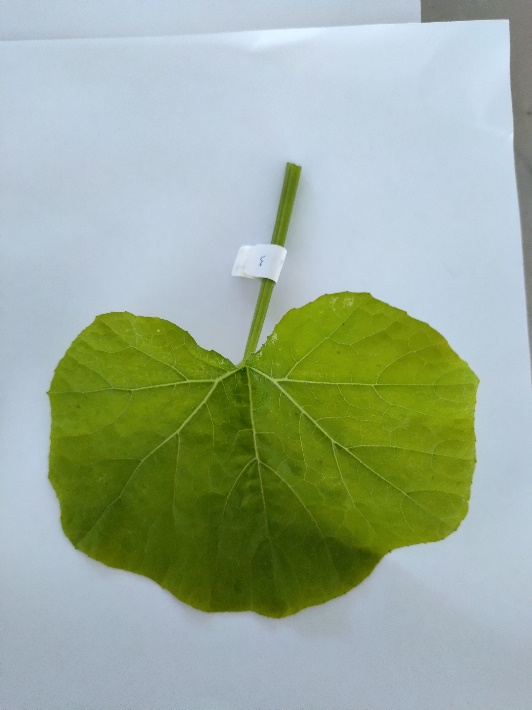 |
| quash | 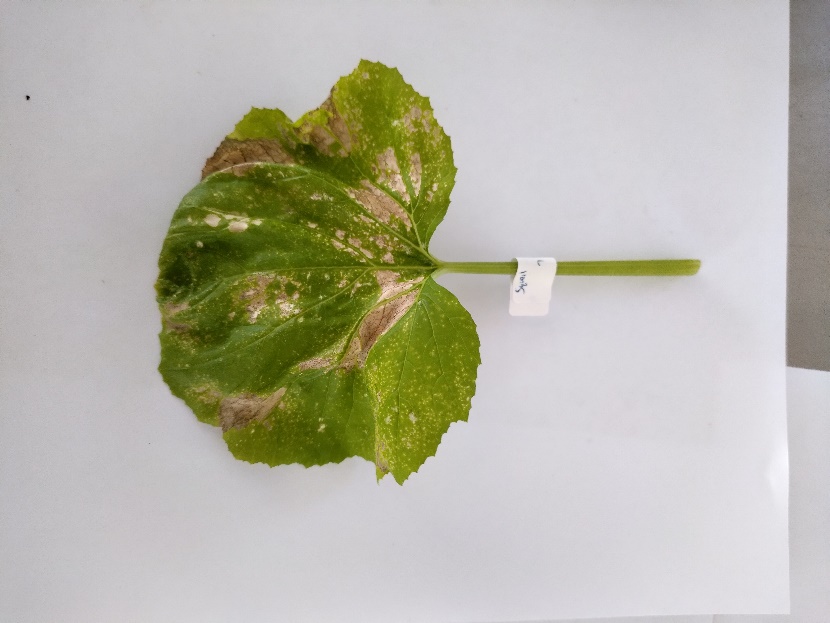 | 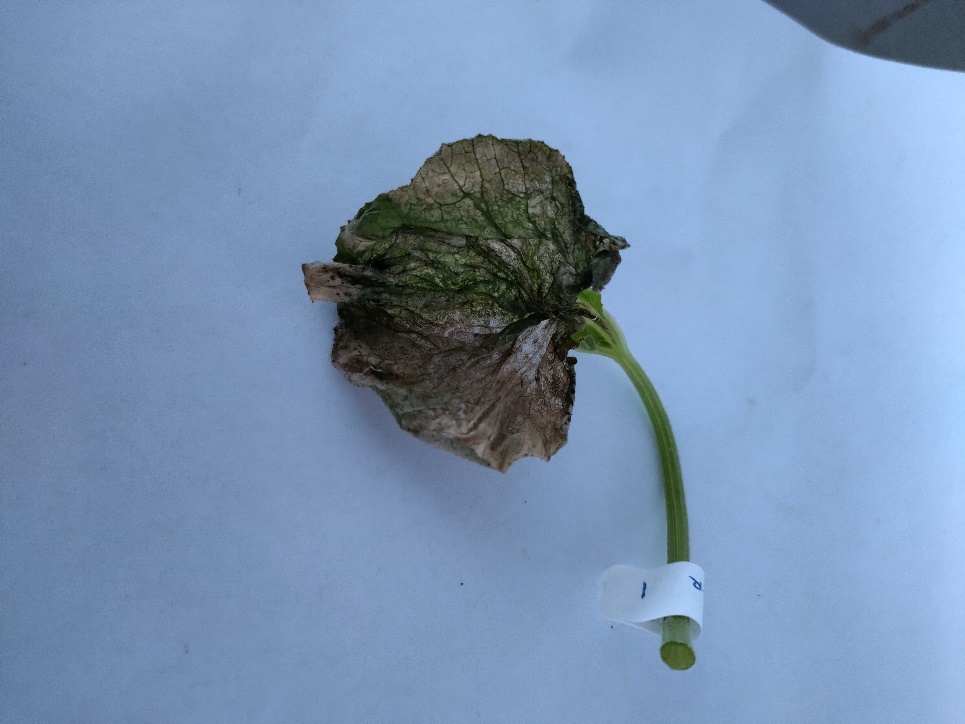 | 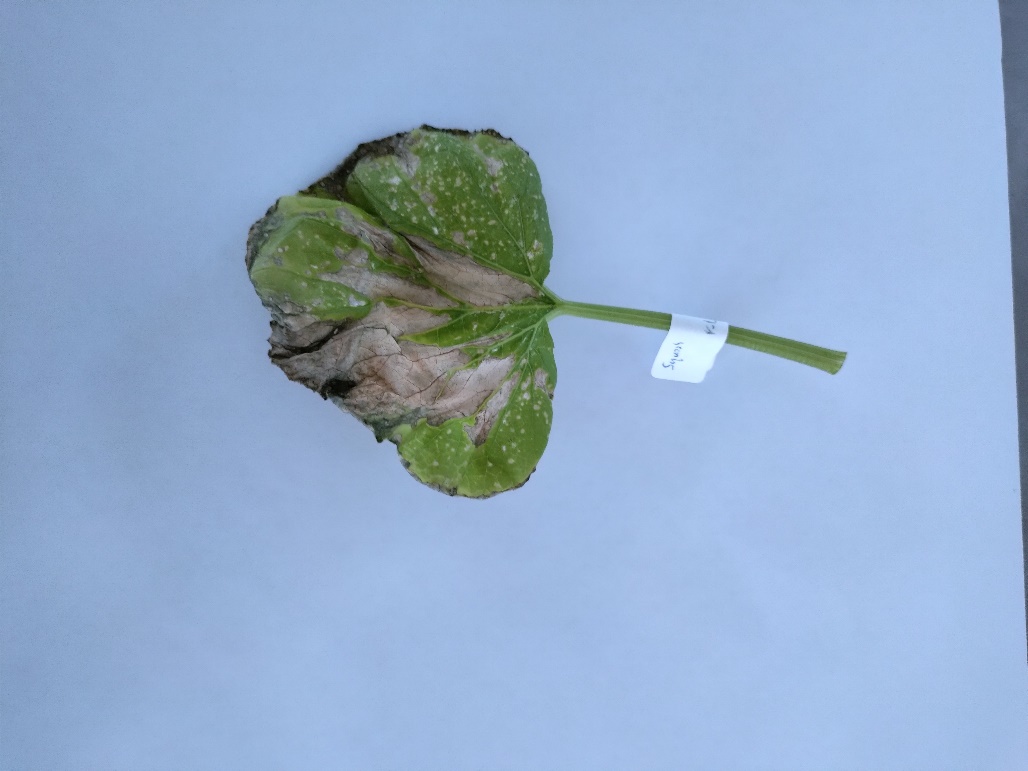 | 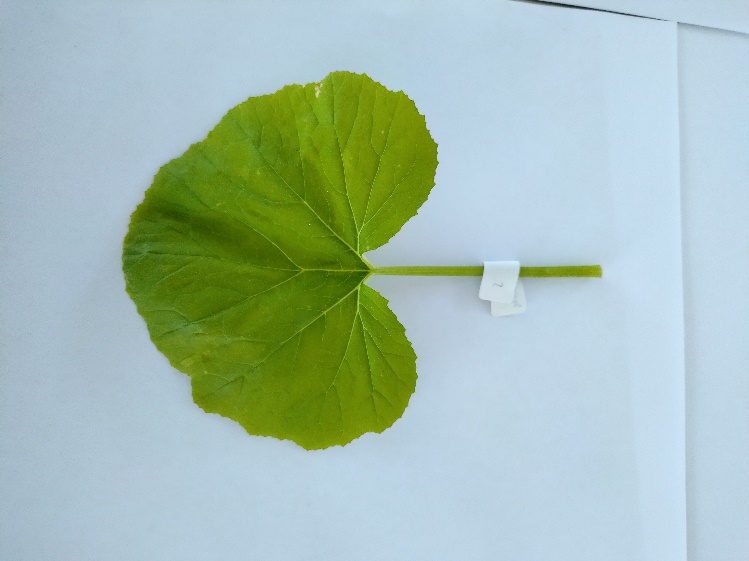 |
| Rockmelon | 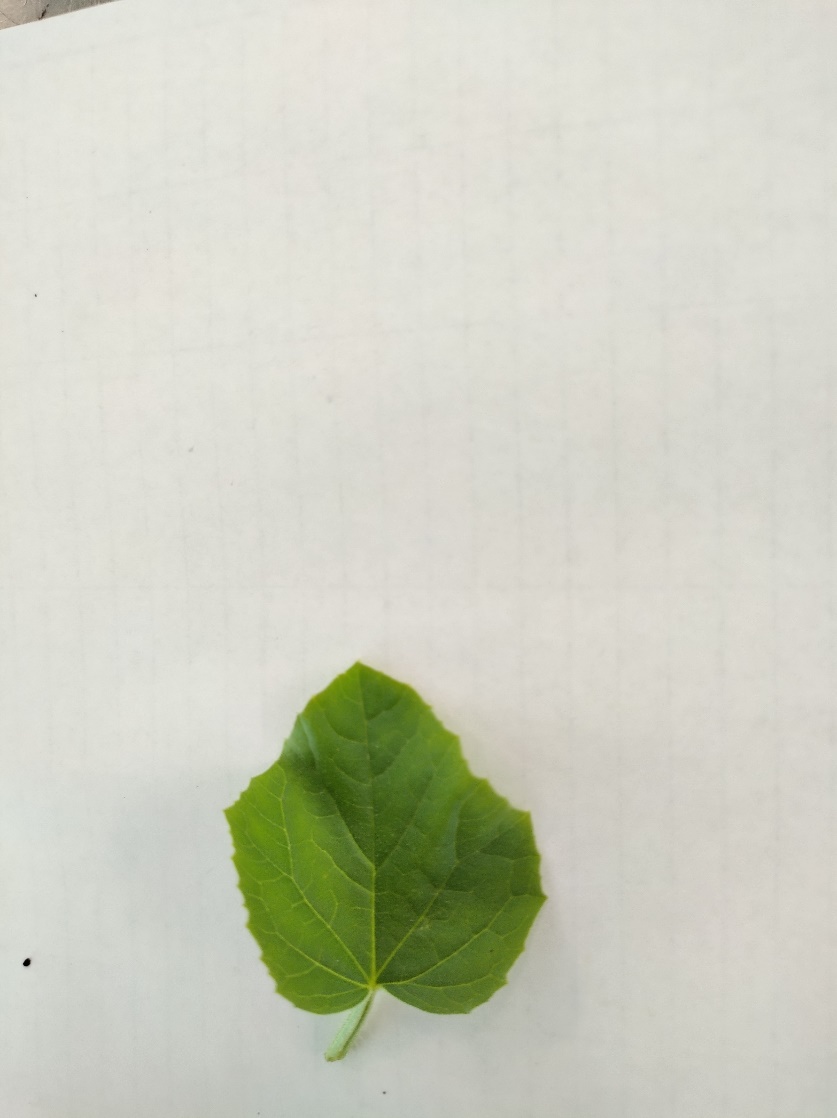 | 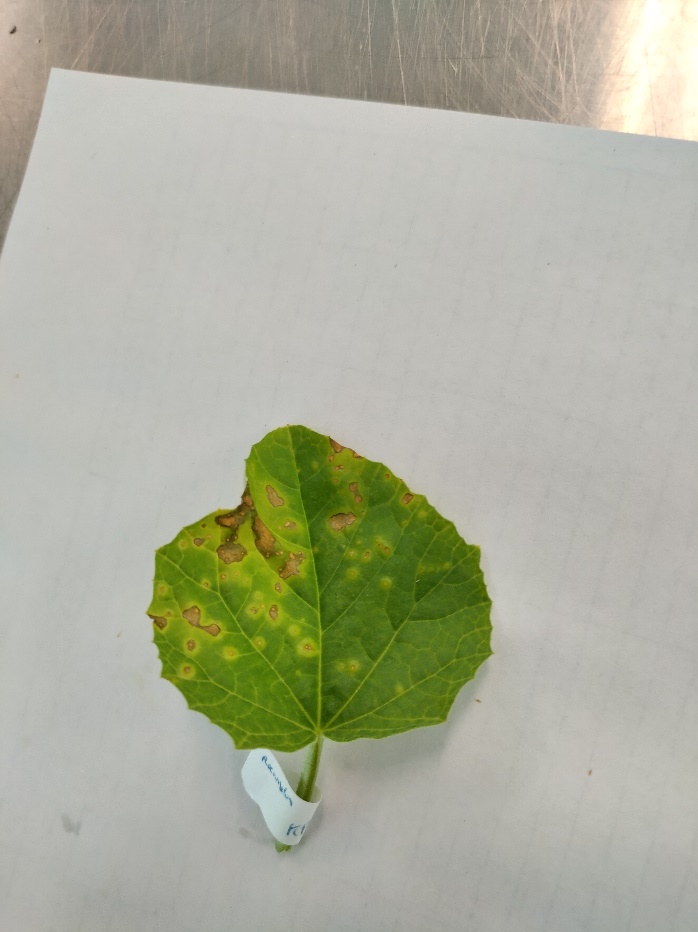 | 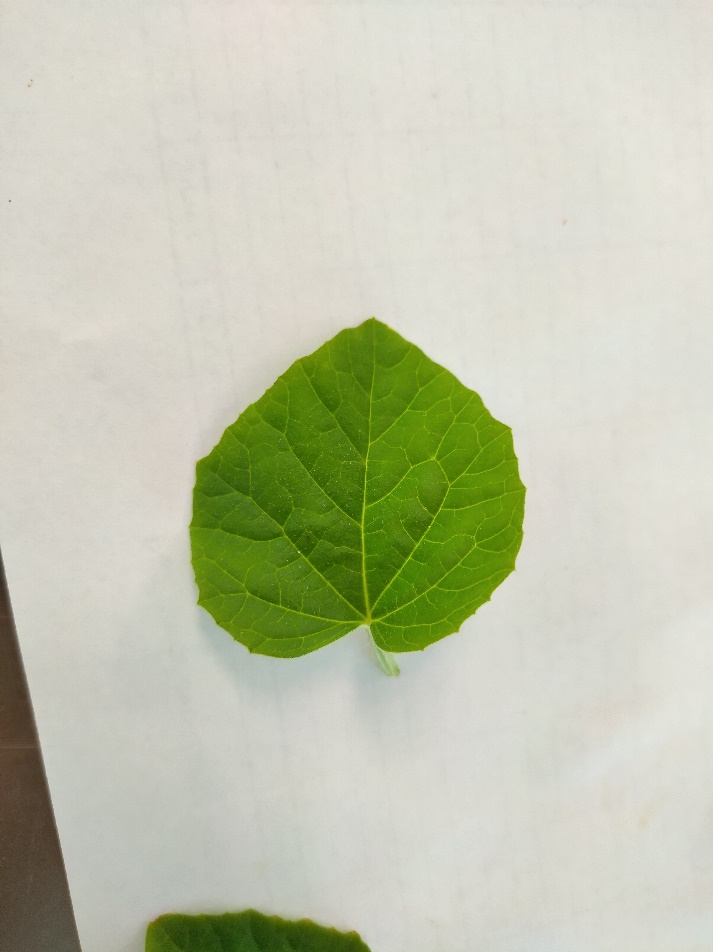 | 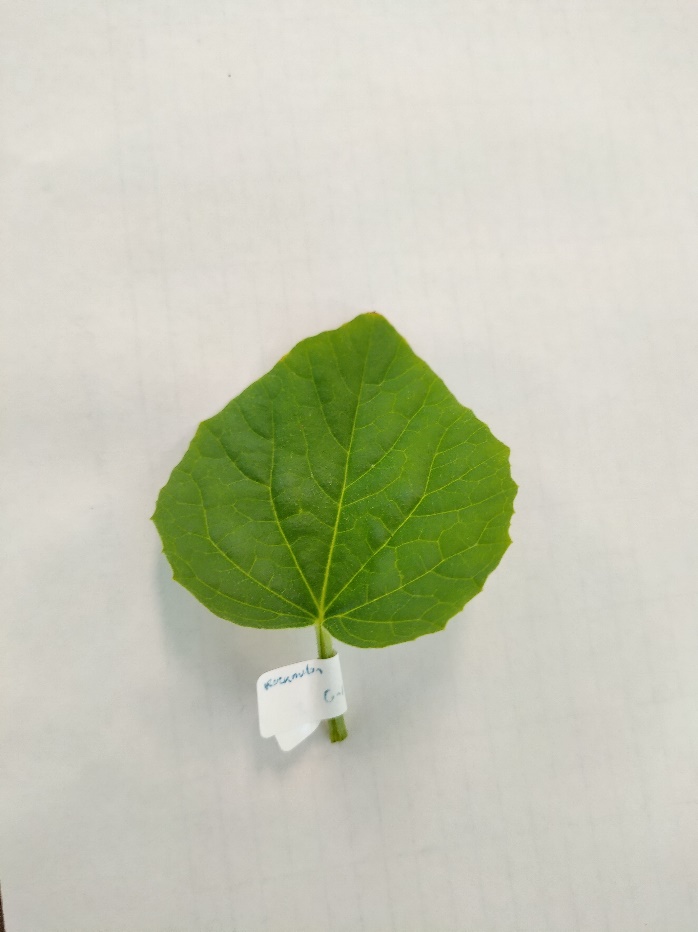 |
| Watermelon | 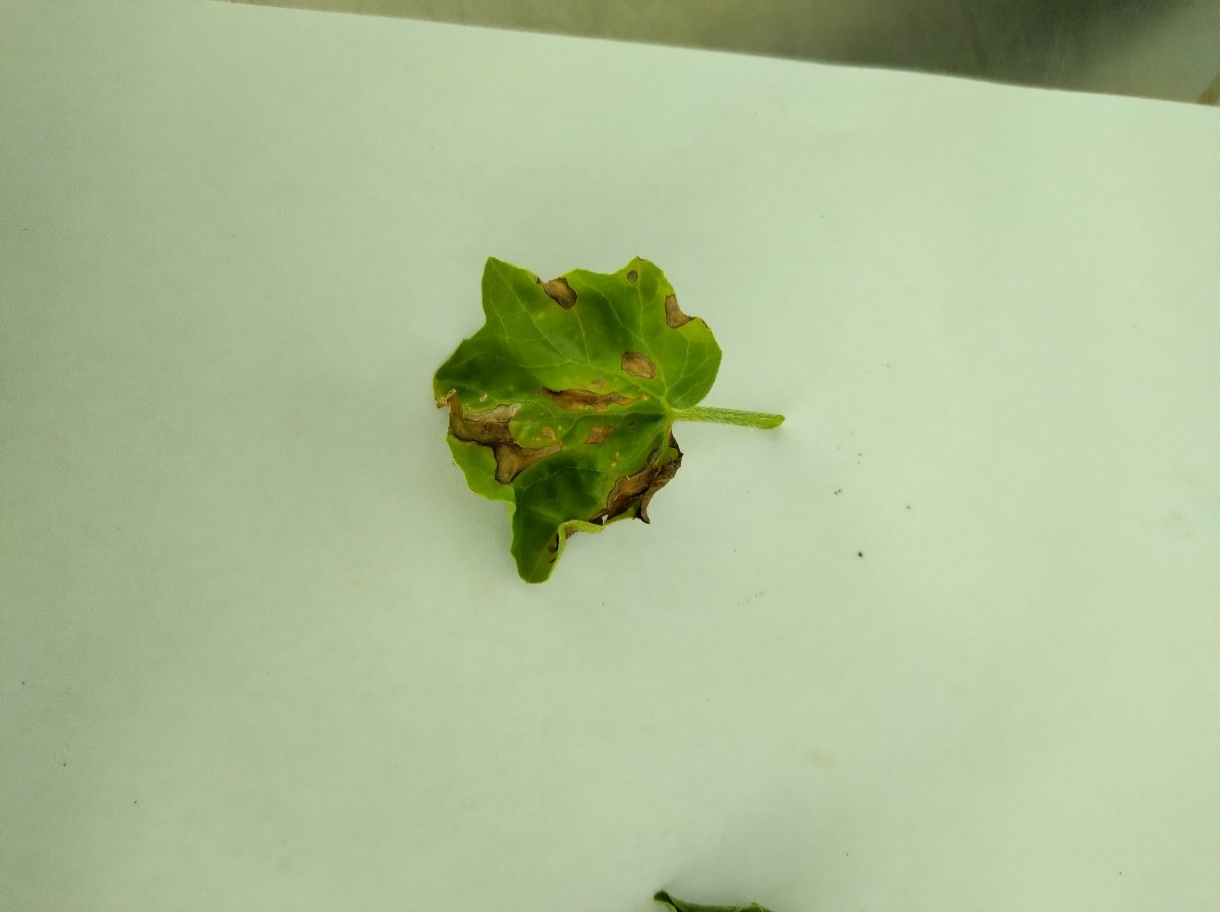 | 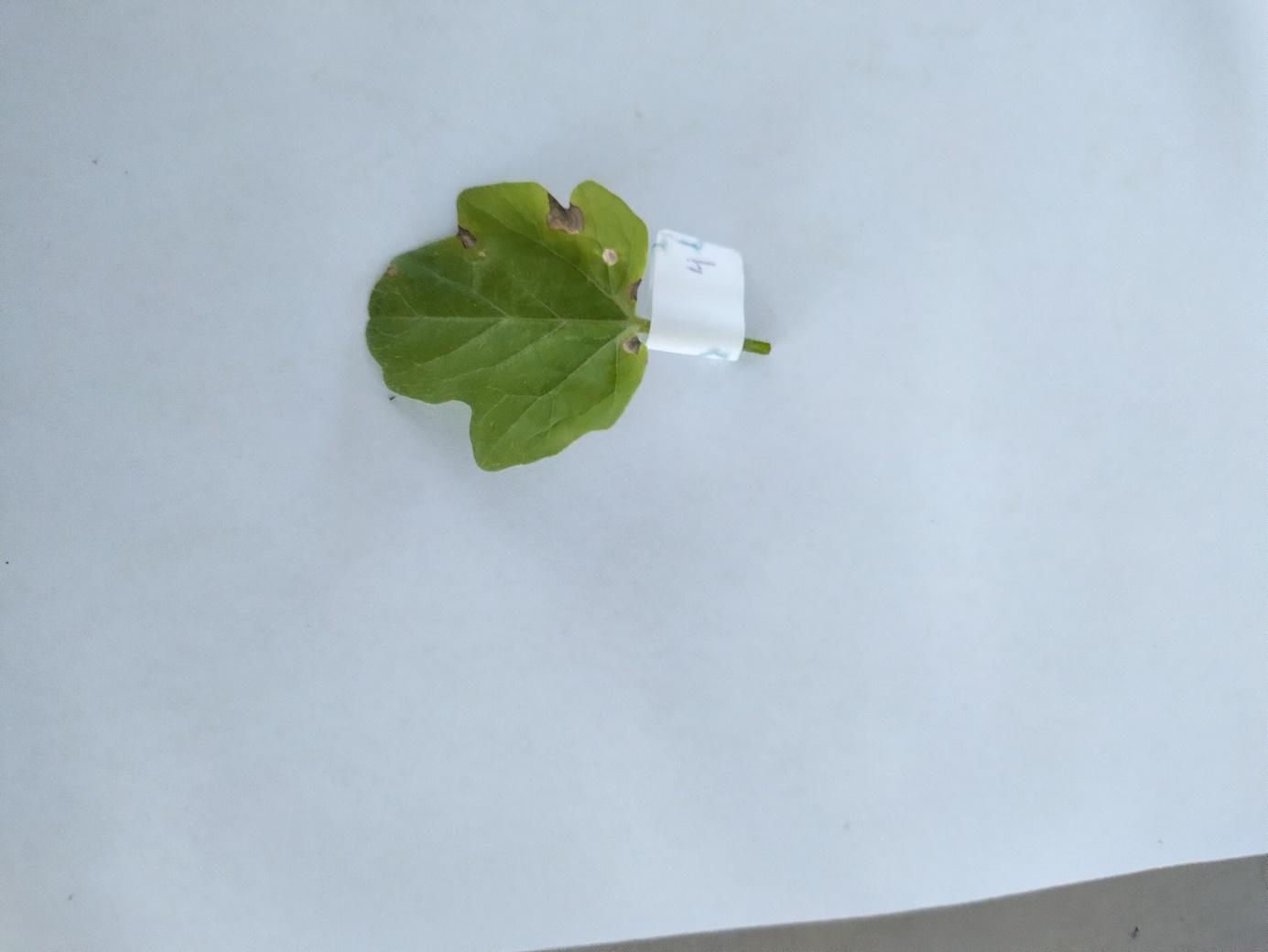 | 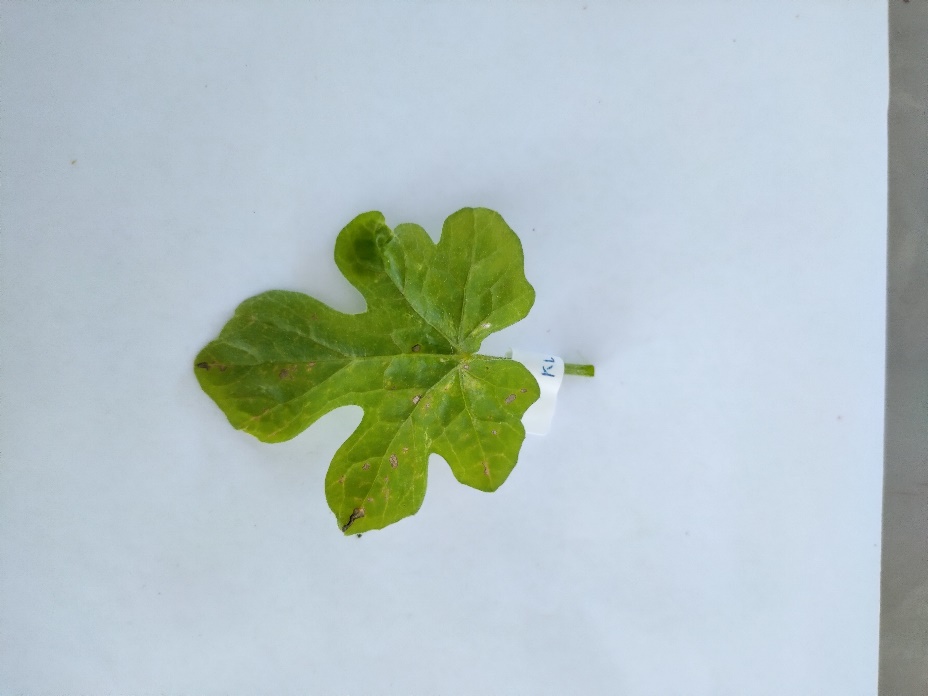 | 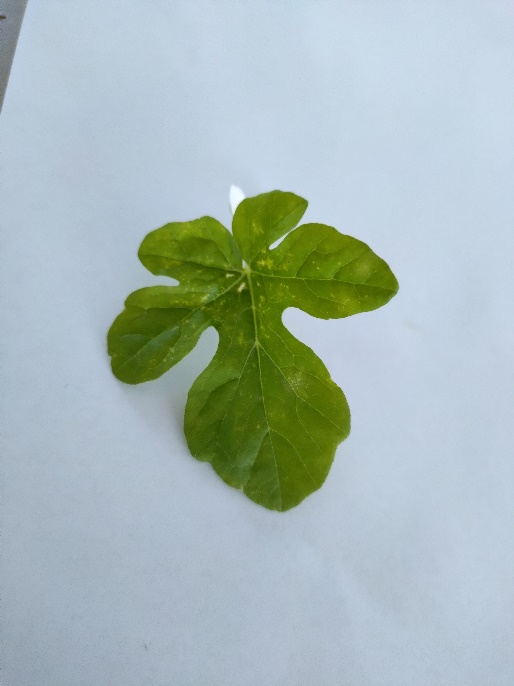 |

**
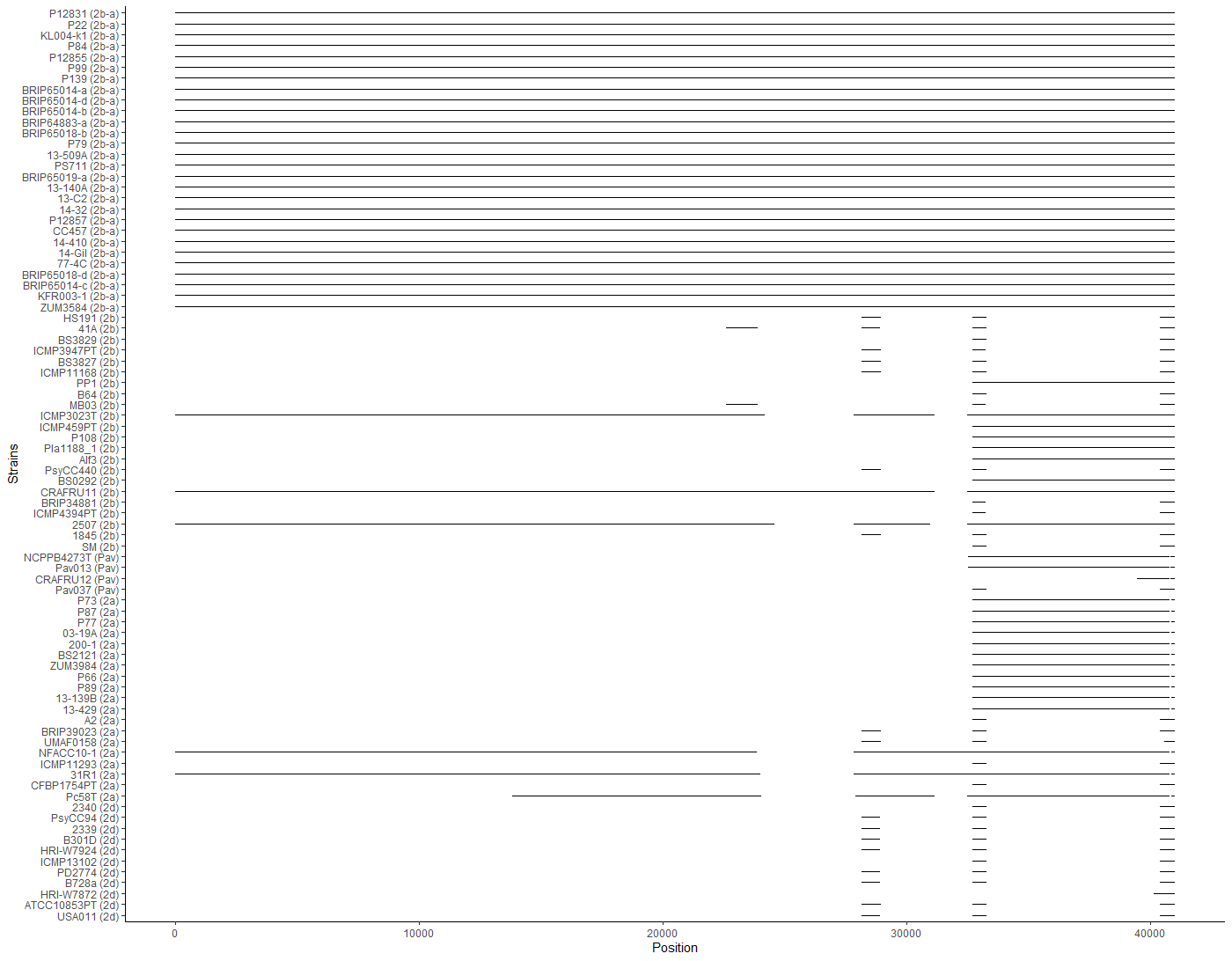
**

**Figure S4** Comparison of RGP_3 DNA sequence in phylogroup 2. The lines indicate the presence of the region in the corresponding strain.


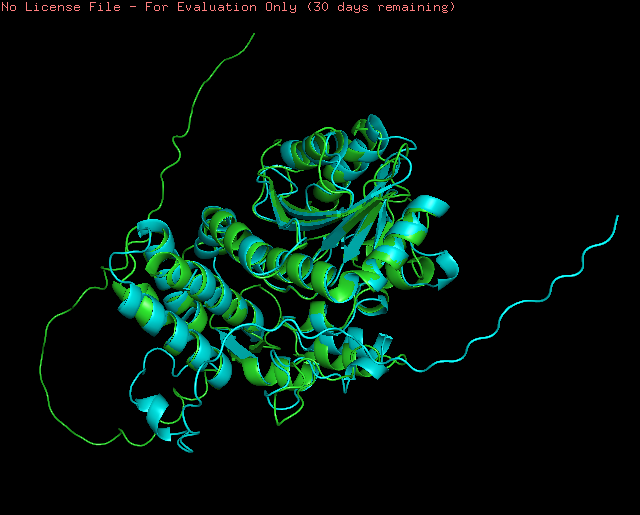


**Figure S5** Structural comparison of KL004-k1 hopZ1 (green) and KFR003-1 hopZ5 (blue). These protein structures were predicted using AlphaFold and compared using PyMOL.

**
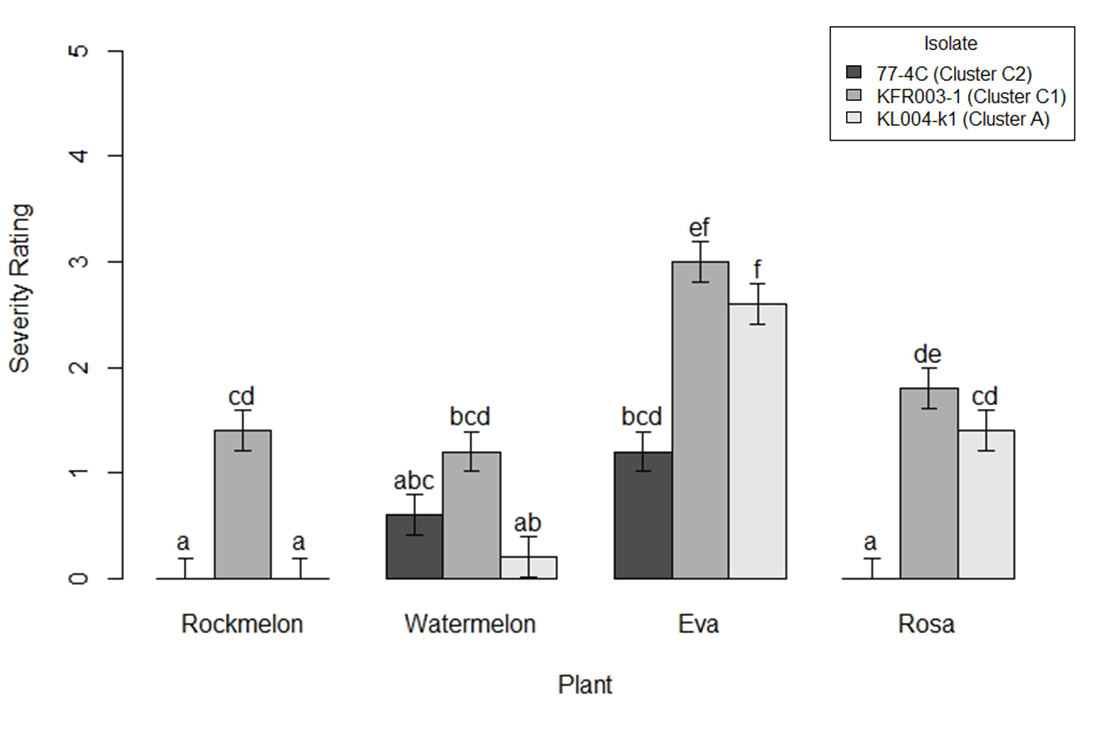
**

**Figure S6** Mean disease severity rating of isolates 77-4C, KFR003-1 and KL004-k1 on cucurbit hosts

. Isolates are identified by colour as shown in the key. ‘Eva’ and ‘Rosa’ are varieties of zucchini. The experiment was conducted in a growth cabinet. Five replicates were used for each isolate on each plant variety. Disease severity was rated from 0-5 which 0 = no symptom, 1 = leaf spots without necrotic lesions, 2 to 5 represented <25%, 25 to 49%, 50 to 74% and ≥75%, necrotic lesions covering the leaf surface, respectively. The vertical lines represent one standard error mean (0.376) calculated with GenStat. Significant differences are shown with a letter above the bar (LSD = 1.07, α = 0.05).

**References:**

1. Newberry EA, Ebrahim M, Timilsina S, Zlatković N, Obradović A, Bull CT, et al. Inference of convergent gene acquisition among *Pseudomonas syringae* strains isolated from watermelon, cantaloupe, and squash. Frontiers in Microbiology. 2019;10(270); doi: https://doi.org/10.3389/fmicb.2019.00270.

2. Seemann T. Prokka: Rapid prokaryotic genome annotation. Bioinformatics. 2014;30(14):2068-9; doi: https://doi.org/10.1093/bioinformatics/btu153.

3. Smirnova AV, Wang L, Rohde B, Budde I, Weingart H, Ullrich MS. Control of temperature-responsive synthesis of the phytotoxin coronatine in *Pseudomonas syringae* by the unconventional two-component system CorRPS. Journal of Molecular Microbiology and Biotechnology. 2002;4(3):191-6.

4. Arrebola E, Carrión VJ, Cazorla FM, Pérez-García A, Murillo J, de Vicente A. Characterisation of the mgo operon in *Pseudomonas syringae* pv. *syringae* UMAF0158 that is required for mangotoxin production. BMC Microbiology. 2012;12(1):10; doi: https://doi.org/10.1186/1471-2180-12-10.

5. Carrión VJ, Arrebola E, Cazorla FM, Murillo J, de Vicente A. The mbo operon is specific and essential for biosynthesis of mangotoxin in *Pseudomonas syringae*. PLOS One. 2012;7(5):e36709; doi: https://doi.org/10.1371/journal.pone.0036709.

6. Aguilera S, López-López K, Nieto Y, Garcidueñas-Piña R, Hernández-Guzmán G, Hernández-Flores JL, et al. Functional characterization of the gene cluster from *Pseudomonas syringae* pv. *phaseolicola* NPS3121 involved in synthesis of phaseolotoxin. J Bacteriol. 2007;189(7):2834-43; doi: https://doi.org/10.1128/JB.01845-06.

7. Ramel C, Baechler N, Hildbrand M, Meyer M, Schädeli D, Dudler R. Regulation of biosynthesis of syringolin A, a *Pseudomonas syringae v*irulence factor targeting the host proteasome. Molecular Plant-Microbe Interactions. 2012;25(9):1198-208; doi: https://doi.org/10.1094/MPMI-03-12-0070-R.

8. Dudnik A, Dudler R. Virulence determinants of *Pseudomonas syringae* strains isolated from grasses in the context of a small type III effector repertoire. BMC Microbiology. 2014;14(1):304; doi: https://doi.org/10.1186/s12866-014-0304-5.

9. Scholz-Schroeder BK, Soule JD, Lu S-E, Grgurina I, Gross DC. A physical map of the syringomycin and syringopeptin gene clusters localized to an approximately 145-kb DNA region of *Pseudomonas syringae* pv. *syringae s*train B301D. Molecular Plant-Microbe Interactions. 2001;14(12):1426-35; doi: https://doi.org/10.1094/MPMI.2001.14.12.1426.

10. Lu SE, Scholz-Schroeder BK, Gross DC. Characterization of the *salA*, *syrF*, and *syrG* regulatory genes located at the right border of the syringomycin gene cluster of *Pseudomonas syringae* pv. *syringae*. Molecular Plant-Microbe Interactions. 2002;15(1):43-53; doi: https://doi.org/10.1094/mpmi.2002.15.1.43.

11. Wang N, Lu S-E, Wang J, Chen ZJ, Gross DC. The expression of genes encoding lipodepsipeptide phytotoxins by *Pseudomonas syringae* pv. *syringae* is coordinated in response to plant signal molecules. Molecular Plant-Microbe Interactions. 2006;19(3):257-69; doi: https://doi.org/10.1094/MPMI-19-0257.

12. Kinscherf TG, Willis DK. The biosynthetic gene cluster for the β-lactam antibiotic tabtoxin in *Pseudomonas syringae*. The Journal of Antibiotics. 2005;58(12):817-21; doi: https://doi.org/10.1038/ja.2005.109.

13. Berti AD, Thomas MG. Analysis of achromobactin biosynthesis by *Pseudomonas syringae* pv. *syringae* B728a. J Bacteriol. 2009;191(14):4594-604; doi: https://doi.org/10.1128/JB.00457-09.

14. Owen JG, Ackerley DF. Characterization of pyoverdine and achromobactin in *Pseudomonas syringae* pv. *phaseolicola* 1448a. BMC Microbiology. 2011;11(1):218; doi: https://doi.org/10.1186/1471-2180-11-218.

15. Bultreys A, Gheysen I, de Hoffmann E. Yersiniabactin production by *Pseudomonas syringae* and *Escherichia coli*, and description of a second yersiniabactin locus evolutionary group. Applied and Environmental Microbiology. 2006;72(6):3814-25; doi: https://doi.org/10.1128/AEM.00119-06.

16. Gross DCC, Y. S.; Proebsting, E. L., Jr.; Radamaker, G. K.; Spotts, R. A. Ecotypes and pathogenicity of ice-nucleation-active *Pseudomonas syringae* isolated from deciduous fruit tree orchards. Phytopathology. 1984;74:241-8; doi: https://doi.org/10.1094/Phyto-74-241.

17. Ravindran A, Jalan N, Yuan JS, Wang N, Gross DC. Comparative genomics of *Pseudomonas syringae* pv. *syringae* strains B301D and HS191 and insights into intrapathovar traits associated with plant pathogenesis. Microbiologyopen. 2015;4(4):553-73; doi: https://doi.org/10.1002/mbo3.261.
